# Panitumumab plus 5-fluorouracil and folinic acid or 5-fluorouracil and folinic acid alone as maintenance therapy in RAS wild-type metastatic colorectal cancer (PanaMa, AIO KRK 0212): final efficacy analysis of a randomised, open-label, phase 2 trial

**DOI:** 10.1016/j.eclinm.2024.103004

**Published:** 2024-12-16

**Authors:** Arndt Stahler, Meinolf Karthaus, Stefan Fruehauf, Ullrich Graeven, Lothar Müller, Ludwig Fischer von Weikersthal, Karel Caca, Eray Goekkurt, Alexej Ballhausen, Greta Sommerhäuser, Annabel H.S. Alig, Swantje Held, Armin Jarosch, David Horst, Anke Reinacher-Schick, Stefan Kasper, Volker Heinemann, Sebastian Stintzing, Tanja Trarbach, Dominik P. Modest

**Affiliations:** a Charité – Universitätsmedizin Berlin, Corporate Member of Freie Universität Berlin and Humboldt-Universität zu Berlin, Department of Hematology, Oncology and Cancer Immunology, Berlin, Germany; b Department of Hematology and Oncology, Munich Hospital Neuperlach, Munich, Germany; c Dr Hancken Hospital, Stade, Germany; d Kliniken Maria Hilf GmbH, Mönchengladbach, Germany; e Oncology Practice UnterEms, Leer, Germany; f Gesundheitszentrum St Marien, Amberg, Germany; g Department of Gastroenterology, Hematology and Oncology, Hospital Ludwigsburg, Ludwigsburg, Germany; h Practice of Hematology and Oncology (HOPE), Hamburg, Germany; i University Cancer Center Hamburg (UCCH), Hamburg, Germany; j ClinAssess GmbH, Leverkusen, Germany; k Charité – Universitätsmedizin Berlin, Corporate Member of Freie Universität Berlin and Humboldt-Universität zu Berlin, Department of Pathology, Berlin, Germany; l German Cancer Consortium (DKTK), German Cancer Research Centre (DKFZ), Heidelberg, Germany; m St Joseph Hospital, Bochum, Germany; n Department of Medical Oncology, West German Cancer Center, Westdeutsches Tumorzentrum, University Hospital of Essen, Essen, Germany; o Department of Medicine 2I and Comprehensive Cancer Center, University Hospital (LMU), Munich, Germany; p Reha-Zentrum am Meer, Bad Zwischenahn, Niedersachsen, Germany

**Keywords:** Metastatic colorectal cancer, Panitumumab, Maintenance, RAS wild-type, Re-induction

## Abstract

**Background:**

The PanaMa trial aimed to compare the efficacy of 5-fluorouracil and folinic acid (FU/FA) ± panitumumab maintenance in untreated *RAS* wild-type metastatic colorectal cancer (mCRC) patients.

**Methods:**

In this final phase 2 trial analysis, adult mCRC patients responding to six cycles of FU/FA, oxaliplatin and panitumumab were randomized (1:1, open-label) to maintenance of either FU/FA + panitumumab or FU/FA alone. The primary endpoint was superiority of progression-free survival of maintenance (PFS; time from random assignment to progression/death) in favour of FU/FA + panitumumab. Secondary endpoints included PFS of re-induction (PFS re-ind.), time to failure of strategy (TFS) and overall survival (OS). The trial is registered with ClinicalTrials.gov (NCT01991873).

**Findings:**

In 248 patients of the Full Analysis Set recruited between May 2014 and February 2021, with a median observation of 64.0 (range 12.5–86.3) months and 59.7 (range 3.7–97.3) months in the treatment arms, 230 events for PFS (92.7%) and 196 events for OS (79.0%) were recorded. Adding panitumumab to FU/FA resulted in significantly longer PFS (8.8 versus 5.8 months, HR = 0.73 (95% CI 0.56–0.94), *P* = 0.015), shorter PFS re-ind. (4.1 versus 7.4 months, HR = 1.93 (95% CI 1.33–2.82), *P* < 0.001), comparable TFS (17.1 versus 15.7 months, HR = 0.98 (95% CI 0.68–1.42), *P* = 0.92) and numerically longer OS (29.9 versus 24.7 months, HR = 0.85 (95% CI 0.64–1.12), *P* = 0.24). The most frequent adverse event (AE) grade ≥3 was rash (FU/FA + panitumumab: n = 15, 12.0%, FU/FA: n = 17, 6.9%). 141 patients (37.3%) experienced at least one serious AE One treatment-related death occurred (neutropenic sepsis, FU/FA).

**Interpretation:**

Panitumumab plus FU/FA might be considered a standard of care maintenance regimen since a potential re-induction therapy with panitumumab cannot be guaranteed at the time of maintenance treatment decision.

**Funding:**

10.13039/100002429Amgen.

**Research in context**

**Evidence before this study**

Literature databases (e.g. PubMed) were searched for evidence regarding the efficacy of maintenance regimens after oxaliplatin-based induction treatment of metastatic colorectal cancer (OPTIMOX1 and OPTIMOX2 trials), the use of targeted therapies as maintenance regimen (DREAM trial), and the efficacy of panitumumab when combined with FOLFOX in *RAS* wild-type metastatic colorectal cancer (PRIME trial). The statistical hypothesis was based on the median PFS observed in OPTIMOX2 and PRIME, respectively.

**Added value of this study**

In addition to previous maintenance studies with a combination of fluoropyrimidine and bevacizumab, the PanaMa study demonstrated a new and effective maintenance strategy for RAS wild-type mCRC patients with the addition of panitumumab to FU/FA. The efficacy of re-induction at the time of disease progression nevertheless depended on the previous panitumumab exposure.

**Implications of all the available evidence**

Although the addition of panitumumab to FU/FA improved the progression-free survival of maintenance consistently to other maintenance trials, the treatment strategy at disease progression is crucial to optimize the overall treatment efficacy. Further research should therefore focus on re-induction versus switch of treatment strategy following maintenance, especially after long-term exposure to panitumumab.

## Introduction

Maintenance treatment, mostly de-escalated cytotoxic regimens in combination with monoclonal antibodies after intensive induction treatment, was established in patients with metastatic colorectal cancer (mCRC) to maintain an initial response, to delay disease progression and to reduce toxicities.^1^, ^2^, ^3^ Common maintenance regimens comprise the application of fluoropyrimidines in combination with monoclonal antibodies directed against the vascular endothelial growth factor (*VEGF*; i.e. bevacizumab)^1^^,^^2^^,^^4^ or epidermal growth factor receptor (*EGFR*; i.e. cetuximab, panitumumab).^3^^,^^5^ The use of maintenance strategies is usually associated with prolonged progression-free survival (PFS) as compared to treatment pause. However, significant effects on the overall survival (OS) of patients are hardly ever observed.^1^, ^2^, ^3^^,^^5^^,^^6^ Whether this is due to the general design of the trials (i.e. the evaluation of maintenance per se) or due to underpowered trials, is an unanswered question.

The PanaMa trial is a randomized, open-label phase 2 trial which compared the maintenance regimens 5-fluorouracil and folinic acid (FU/FA) with or without panitumumab after successful (i.e. at least stable disease) FU/FA + oxaliplatin (mFOLFOX6) and panitumumab first-line induction treatment of patients with *RAS* wild-type mCRC. Patients who experienced disease progression during maintenance treatment had the option to receive induction treatment (re-induction) again. FU/FA plus panitumumab maintenance was associated significantly with longer PFS to FU/FA alone in the confirmatory analysis of the trial.^3^ Correspondingly, in the immature overall survival (OS) analysis, a numerical but not significant advantage was observed. Subgroup analyses suggested hyperselection of tumors with wild-type status for *KRAS*, *NRAS*, *BRAF* V600E, *AKT1, ERBB2, PIK3CA* exon 9/20, *PTEN* and *ALK1* mutations and also the gene-expression based consensus molecular subtypes (CMS) 2 and 4 as potential factors to identify patients deriving the greatest benefit from anti-EGFR-based maintenance therapy.^7^^,^^8^

The present analysis was conducted to provide final efficacy results for PFS, PFS of re-induction (PFS re-ind.), the time to failure of strategy (TFS) according to treatment arms, and also evaluate OS with a mature event rate in all patients and according to clinical and molecular subgroups, together with updated safety data.

## Methods

### Trial design and analysis set

Details regarding the PanaMa trial design, inclusion/exclusion criteria, randomisation procedure and treatment schedule were published previously.^3^ The full trial protocol is also available in the Supplementary Data.

Briefly, adult patients with histologically confirmed, untreated *RAS* wild-type metastatic colorectal cancer received intravenous induction treatment for six cycles with mFOLFOX6 (oxaliplatin 85 mg/m^2^ and FU/FA (folinic acid 400 mg/m^2^, 5-fluorouracil 2400 mg/m^2^ administered over 48 h) plus panitumumab (6 mg/kg body weight), followed by random assignment 1:1 in case of at least stable disease to either FU/FA plus panitumumab (Arm A) or FU/FA maintenance (Arm B) until unacceptable toxicity or disease progression. Re-induction/Re-escalation with mFOLFOX6 plus panitumumab was recommended at disease progression during maintenance therapy for all patients. The recruitment period was between May 2014 and February 2021 in 70 German centres.

### Randomisation and masking

1:1 random assignment was performed centrally by electronic case report form using permuted block randomization, sizes 4 and 6. Stratification factors included objective response to therapy after induction therapy, prior adjuvant treatment with oxaliplatin and planned full versus reduced dose of panitumumab during maintenance therapy. Masking was not performed as this was an open-label phase 2 trial.

### Trial registration

The trial was registered at clinicaltrials.gov (NCT01991873).

### Analysis sets of the trial population

The safety set consisted of all patients that received induction treatment. The full analysis set included all randomized patients who received at least one dose of maintenance treatment according to the study protocol. All analyses were performed with patients of the full analysis set only.

### Endpoints

The study design and corresponding endpoints are graphically displayed in Supplementary Figure S1. The primary endpoint was PFS of maintenance treatment, defined as time from randomization to first disease progression according to RECIST 1.1 criteria (locally assessed) or death from any cause. Secondary endpoints, among others, were PFS re-ind., defined as the time length between the date of objective disease progression during maintenance and the date of first disease progression (according to RECIST criteria 1.1) or death after start of re-induction (whichever occurs first); TFS, defined as the time from randomisation to second objective disease progression, or death from any cause, whichever first, patients without re-induction therapy were censored after maintenance therapy; OS of maintenance treatment, defined as time from randomization to death from any cause; objective response rates (ORR) of maintenance and re-induction treatment by RECIST 1.1 criteria; OS of re-induction treatment, defined as time from start of re-induction to death from any cause (exploratory endpoint which was not pre-specified in the study protocol). If patients did not experience progression or death, data were censored at the last date known event-free. The last database update on overall survival was performed in October 2023 followed by the final analysis in December 2023.

### Adverse events

Adverse events were assessed according to the grading of the National Cancer Institute Common Terminology Criteria for Adverse Events (version 4.03) from registration into the trial until the final study visit.

### Analysis and definition of hyperselection status and CMS classification

Methodological details of mRNA gene expression including calling of the CMS, DNA and immunohistochemical analyses were published previously.^7^^,^^8^

The hyperselection mutation (MUT) subgroup was defined by the presence of HER2/neu overexpression (3+) or at least one pathogenic point mutation of the following genes detected in a tumor: *KRAS, NRAS, BRAF* (V600E only), *AKT1, ERBB2, PIK3CA* exon 9/20, *PTEN*, *ALK1* and the wild-type (WT) subgroup by absence of HER2/neu overexpression or pathogenic mutations. CMS were assessed using the *predictedCMS* approach, resulting in optimized sensitivity.^9^

### Ethics

The PanaMa trial was conducted according to the Declaration of Helsinki and approved by the local ethics committees of all participating centres (no. 425/13(FF-MC)). Written informed consent was provided before participation.

### Statistics

The hypothesis and statistical design of the PanaMa trial were described previously. Briefly, a median PFS of 7.5 months in the FU/FA arm was assumed as a standard of care treatment efficacy and the trial aimed to improve this maintenance treatment interval to 10.0 months by adding panitumumab, corresponding to a HR of 0.75. 218 events for PFS with 80% power and a (one-sided) α-error of 0.10 were necessary to detect this difference. The two-sided significance level for secondary and exploratory end points was set to 0.05.

The median survival in months of the time-to-event endpoints including the 95% confidence interval (95% CI) was estimated by the Kaplan–Meier method. Statistical comparisons were made by the log-rank test. Cox regression analyses were performed for PFS and OS of maintenance treatment, PFS of re-induction treatment and time to failure of strategy. Multivariate analyses were performed with baseline characteristics and molecular characteristics to assess significant independent prognostic variables. ORR were compared by treatment arms using Fisher's exact test (alpha = 0.20). SPSS PASW 23.0 (SPSS, Chicago, IL) and R v3.6.1 software were used for statistical analyses.

### Role of the funding source

The legal funder (sponsor) of this study was the AIO Studien GmbH, Germany, which participated in designing the study. Amgen Inc, Thousand Oaks, CA, which provided financial support for this study, did not influence the study design, the collection, analysis, and interpretation of data nor the writing of the report. All parties agreed with publication of this report.

## Results

### Patients

**Fig. 1:**
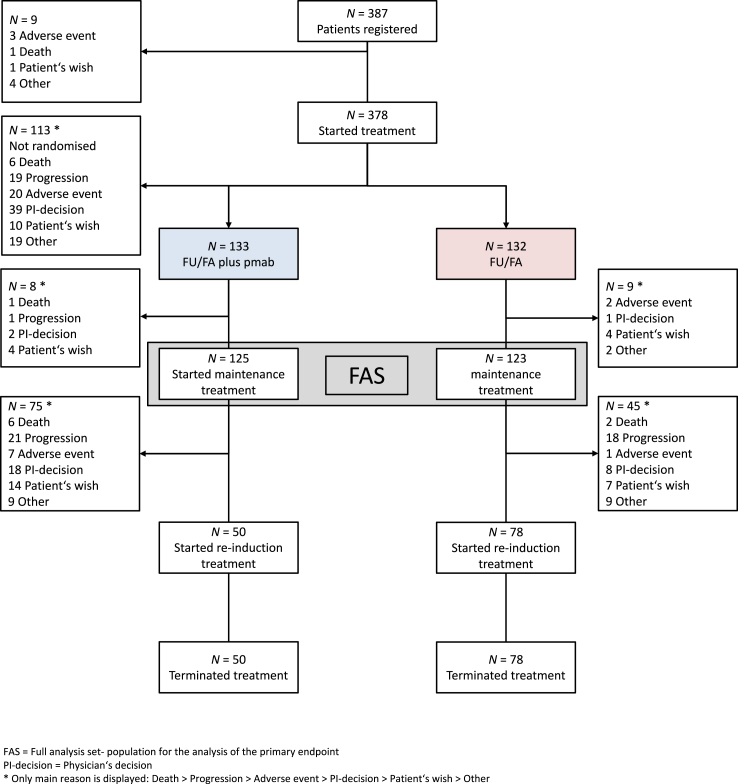
CONSORT diagram and subpopulations evaluable for secondary endpoints of the randomized phase 2 open-label PanaMa trial. FU/FA, 5-fluorouracil and folinic acid; pmab, panitumumab; FAS, Full Analysis Set; PI-decision, Physician's decision.

The full analysis set of the PanaMa trial consisted of *N* = 248 patients (FU/FA plus panitumumab: *N* = 125; FU/FA: *N* = 123). For PFS re-ind. and TFS, *N* = 128 (FU/FA plus panitumumab: *N* = 50; FU/FA: *N* = 78) and *N* = 248 (FU/FA plus panitumumab: *N* = 125; FU/FA: *N* = 123) patients, respectively, were evaluable (Fig. 1).

Hyperselection analyses were possible in *N* = 202 (81.5%) patients (WT: *N* = 162; MUT: *N* = 40), whereas CMS calling was successful in *N* = 196 patients (CMS1, *N* = 10; CMS2, *N* = 82; CMS3, *N* = 20; CMS4, *N* = 73; not evaluable, *N* = 11), as reported.^7^^,^^8^

The baseline characteristics of patients and tumors receiving maintenance treatment are summarized in Table 1 and of those receiving re-induction treatment in Supplementary Table S1. Baseline characteristics of patients who did not receive re-induction treatment, subsequent treatments and detailed justifications not to perform re-induction treatment by the respective investigator are listed in Supplementary Table S2.

**Table 1:** Updated baseline characteristics of patients included in the full analysis set and respective treatment arms of the PanaMa trial.

Variable	All randomized and treated patients (*N* = 248)	FU/FA plus panitumumab (*N* = 125)	FU/FA (*N* = 123)
**Sex, *N* (%)**			
Female	83 (33.5)	38 (30.4)	45 (36.6)
Male	165 (66.5)	87 (69.6)	78 (63.4)
**Age**			
Median in years (range)	65 (30–86)	66 (44–84)	65 (30–86)
**ECOG, *N* (%)**			
0	147 (59.3)	70 (56.0)	77 (62.6)
1	101 (40.7)	55 (44.0)	46 (37.4)
**Ethnicity, *N* (%)**			
Caucasian	247 (99.6)	125 (100.0)	122 (99.2)
Asian	1 (0.4)	0 (0.0)	1 (0.8)
**Body mass index**			
Median (range)	25.5 (16.5–46.8)	25.5 (17.3–46.8)	25.5 (16.5–43.3)
**Baseline CEA level**			
Median (ng/ml)	41.9	34.4	47.2
**Previous resection of primary tumor, *N* (%)**			
Yes	176 (71.0)	94 (75.2)	82 (66.7)
**Previous radiation, *N* (%)**			
Yes	28 (11.3)	14 (11.2)	14 (11.4)
**Prior adjuvant therapy, *N* (%)**			
All therapies	26 (10.5)	12 (9.6)	14 (11.4)
Oxaliplatin-based	12 (4.8)	8 (6.4)	4 (3.3)
**One prior cycle of FOLFOX, *N* (%)**			
Given	29 (11.7)	12 (9.6)	17 (13.8)
**Primary tumor location, *N* (%)**			
Left-sided	199 (80.2)	99 (79.2)	100 (81.3)
Right-sided	38 (15.3)	19 (15.2)	19 (15.4)
Both	10 (4.0)	6 (4.8)	4 (3.3)
Unclear	1 (0.4)	1 (0.8)	0 (0.0)
**Metastatic sites, *N* (%)**			
Liver	205 (82.7)	100 (80.0)	105 (85.4)
Liver-limited	102 (41.1)	53 (42.4)	49 (39.8)
Lung	62 (25.0)	28 (22.4)	34 (27.6)
Lymph nodes	80 (32.2)	45 (36.0)	35 (28.5)
Peritoneum	38 (15.3)	13 (10.4)	25 (20.3)
Other	32 (12.9)	10 (8.0)	22 (17.9)
**No. of organs involved, *N* (%)**			
1	132 (53.2)	70 (56.0)	62 (50.4)
>1	116 (46.8)	55 (44.0)	61 (49.6)
**Onset of metastatic disease, *N* (%)**			
Synchronous	200 (80.6)	101 (80.8)	99 (80.5)
Metachronous	48 (19.4)	24 (19.2)	24 (19.5)

FU/FA, 5-fluorouracil and folinic acid; ECOG, Eastern Cooperative Oncology Group; CEA, carcinoembryonic antigen; FOLFOX, 5-fluorouracil, folinic acid, oxaliplatin; no., number.

### Efficacy analyses by treatment arms in randomized and treated patients

With a median follow-up of 64.0 months (FU/FA plus panitumumab) and 59.7 months (FU/FA), 230 events for PFS (92.7%) and 196 events for OS (79.0%) were reported, respectively. The median treatment duration of FU/FA plus panitumumab and FU/FA was 5.49 and 4.63 months for maintenance and 1.87 and 3.94 months for re-induction. With regard to the whole study treatment duration, 10.41 and 11.79 months were observed in the Full Analysis Set and 13.82 and 13.93 months in patients who received re-induction.

**Fig. 2:**
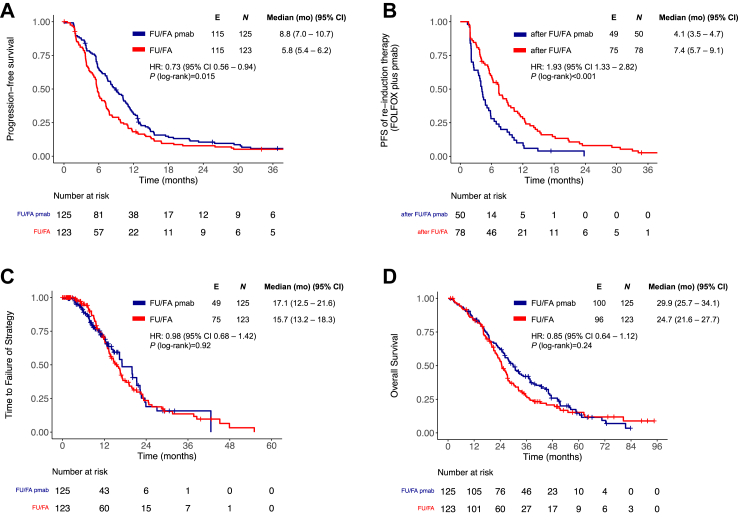
**(A)** Progression-free survival, **(B)** progression-free survival of re-induction therapy **(C)** time to failure of strategy and **(D)** overall survival of the randomized phase 2 open-label PanaMa trial Full Analysis Set, according to treatment arms. FU/FA, 5-fluorouracil and folinic acid; pmab, panitumumab; FOLFOX, 5-fluorouracil; folinic acid, oxaliplatin; PFS, progression-free survival; mo, months; HR, hazard ratio; CI, confidence interval.

In randomized and treated patients, PFS was significantly longer (8.8 versus 5.8 months, HR = 0.73 (95% CI 0.56–0.94), *P* = 0.015) and PFS re-ind. significantly shorter for FU/FA plus panitumumab compared to FU/FA maintenance (4.1 versus 7.4 months, HR = 1.93 (95% CI 1.33–2.82), *P* < 0.001). TFS was comparable (17.1 versus 15.7 months, HR = 0.98 (95% CI 0.68–1.42), *P* = 0.92) and OS was numerically, but not statistically significant, longer (29.9 versus 24.7 months, HR = 0.85 (95% CI 0.64–1.12), *P* = 0.24) for panitumumab added to FU/FA (Fig. 2A–D). OS of re-induction was comparable in both arms (Supplementary Figure S2).

### Multivariate cox regression analyses of prognostic clinical and molecular biomarkers

In clinical subgroup analyses, multivariate Cox regression identified the number of organs involved (*P* = 0.04) as independent prognostic variable for PFS. Age (*P* = 0.002), the number of organs involved (*P* = 0.02) and previous surgery of the primary tumor (*P* < 0.001) were independent predictors of OS (Supplementary Table S3).

Mutational hyperselection status as described above was an independent prognostic biomarker for OS (*P* = 0.002) (Supplementary Table S3).

### Objective response rates according to maintenance and re-induction treatment

The addition of panitumumab to FU/FA versus FU/FA alone led to higher ORR during maintenance (40.8% versus 29.3%), but lower ORR at re-induction (8.0% versus 35.9%) (Table 2).

**Table 2:** Objective response rate according to all treatment phases in the PanaMa trial.

Variables	ORR of maintenance *N* = 248		ORR of re-induction *N* = 128	
	FU/FA plus panitumumab *N* = 125	FU/FA *N* = 123	FU/FA plus panitumumab *N* = 50	FU/FA *N* = 78
Response, N (%)				
CR	9 (7.2)	7 (5.7)	0 (0.0)	4 (5.1)
PR	42 (33.6)	29 (23.6)	4 (8.0)	24 (30.8)
SD	50 (40.0)	47 (38.2)	18 (36.0)	24 (38.0)
PD	17 (13.6)	30 (24.4)	18 (36.0)	12 (15.4)
NE	7 (5.6)	10 (8.1)	10 (20.0)	14 (17.9)
ORR, N (%)	51 (40.8%)	36 (29.3%)	4 (8.0%)	28 (35.9%)
(80% CI)	(32.1%–49.9%)	(21.4%–38.1%)	(2.2%–19.2%)	(25.3%–47.6%)
Odds ratio (80% CI)	0.60 (0.43–0.85)		6.45 (3.06–13.33)	
*P* (Fisher's exact test, two-sided)	0.06		**<0.001**	

ORR, objective response rate; FU/FA, 5-fluorouracil and folinic acid; CI, confidence interval; CR, complete remission; PR, partial remission; SD, stable disease; PD, progressive disease; NE, not evaluable; *P*-values considered significant are displayed **bold**.

### Predictive implications of clinical and molecular subgroups for panitumumab efficacy

**Fig. 3:**
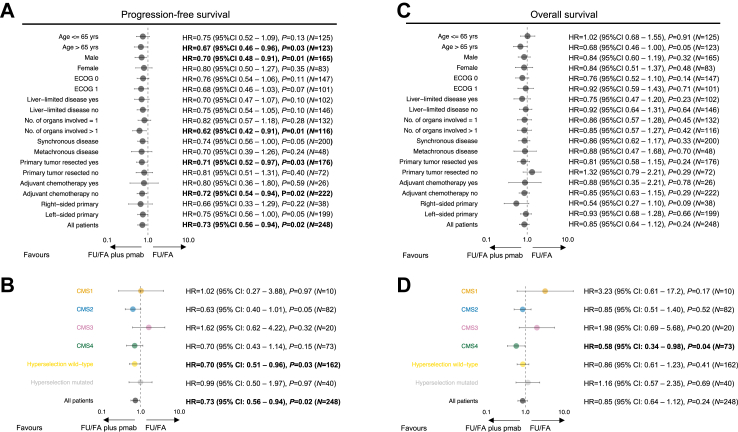
Univariate Cox regression analyses performed in subgroups for (A) progression-free survival of maintenance treatment by baseline and (B) molecular characteristics and (C) overall survival of maintenance treatment by baseline and (D) molecular characteristics, displayed as forest plot. Results considered significant are displayed **bold**. FU/FA, 5-fluorouracil and folinic acid; pmab, panitumumab; yrs, years; ECOG, Eastern Cooperative Oncology Group; No., number; CMS, consensus molecular subtype; HR, hazard ratio; CI, confidence interval.

Numerically, for PFS all analyzed clinical subgroups trended towards better outcome with FU/FA plus panitumumab versus FU/FA alone. Significant effects of PFS when panitumumab was added were observed in patients aged older than 65 years, male sex, more than one organ involved, previous resected primary tumors and without adjuvant chemotherapy (Fig. 3A). In molecular analyses, CMS2 and CMS4 tumors as well as hyperselection wild-type status were likely associated with benefit from panitumumab (Fig. 3B).

Consistently with the OS analysis, less pronounced effects of panitumumab were observed rather homogeneously across the clinical and molecular subgroups (Fig. 3C and D).

### Subgroup analysis of combined hyperselection status and primary tumor sidedness

PFS re-ind. and OS were significantly longer in hyperselection wild-type left- and right-sided primary tumors in multivariate analysis, whereas no differences were observed for PFS and TFS and other constellations of hyperselection status and primary tumor sidedness (Supplementary Table S4).

PFS was numerically longer and PFS re-ind. significantly shorter when panitumumab was added in hyperselection wild-type left-sided primary tumors. Treatment arms were equally effective according to other endpoints and constellations of hyperselection status and primary tumor sidedness (Supplementary Table S5).

### Adverse events during maintenance therapy and re-induction therapy

The most frequent adverse event (AE) grade ≥3 was rash (FU/FA + panitumumab: n = 15, 12.0%, FU/FA: n = 17, 6.9%). 141 patients (37.3%) experienced at least one serious AE One treatment-related death occurred (neutropenic sepsis, FU/FA).

During maintenance therapy, 97.6% (FU/FA plus panitumumab) and 90.2% (FU/FA) patients experienced an adverse event, and of those 60.8% and 30.9% were grade ≥3. Events leading to a permanent discontinuation of therapy were observed in 15.2% (FU/FA plus panitumumab) and 1.6% (FU/FA) of patients. Grade 5 events affected 3 patients in each treatment arm (2.4%) (Supplementary Table S6).

During re-induction therapy with mFOLFOX6 plus panitumumab adverse events were reported in 92.0% (prior FU/FA plus panitumumab) and 91.0% (prior FU/FA) of patients, of those 40.0% and 52.6% were grade ≥3. Permanent discontinuation of therapy due to adverse events was observed in 10.0% (prior FU/FA plus panitumumab) and 14.1% (prior FU/FA). Grade ≥3 events during re-induction therapy were reported in 4.0% of patients treated with previous FU/FA plus panitumumab maintenance and 1.3% of patients pretreated with FU/FA maintenance (Supplementary Table S6).

## Discussion

This report was performed to provide the final analysis of the primary endpoint (PFS of maintenance), analyses of secondary efficacy endpoints such as PFS of re-induction and time to failure of strategy and also to evaluate the key secondary endpoint, overall survival, with a mature event rate in the full analysis set.

This final PFS analysis confirms the primary analysis of the trial, demonstrating a significant improvement of PFS of maintenance therapy with the addition of panitumumab to a fluoropyrimidine based regimen.^3^ Interestingly, the PFS of re-induction of mFOLFOX6 + panitumumab was significantly shorter and the ORR of re-induction significantly lower after FU/FA plus panitumumab, while the time to failure of strategy was consecutively comparable between the arms. In addition, with 79% events, overall survival of maintenance treatment, despite being numerically longer with FU/FA plus panitumumab, was, consistently with other maintenance trials, not significantly improved by adding panitumumab to FU/FA maintenance.^1^^,^^2^^,^^5^^,^^10^, ^11^, ^12^

Although these results may appear contradictory at first glance, they however provide a plausible overall picture when several aspects are considered and synthesized.

First, all patients in the PanaMa trial received treatment with panitumumab during induction treatment before entering the randomized maintenance phase and must have experienced at least stable disease to proceed, meaning that only patients who did not progress during induction therapy received maintenance at all. Consequently, patients with early disease progression were not included in this trial and the likelihood of receiving more than one treatment lines was therefore higher for this study population. Eventually, this bias might have compensated for the missed antibody during maintenance. This perspective is clearly supported by the longer PFS and also higher ORR of re-induction therapy observed in the PanaMa trial, which was longer in patients who received FU/FA alone as maintenance treatment. However, the median PFS in both treatment arms was lower than expected despite an adequate relative effect size, which could be due to additional confounders such as the non-exclusion of microsatellite-instable tumors or other prognostically relevant molecular subgroups. In turn, re-escalation with oxaliplatin after FU/FA plus panitumumab maintenance should be critically discussed, as the additional benefit by re-escalation at disease progression might be limited—albeit taking into account the limited sample size. From a biological perspective, long-term therapy with an anti-EGFR directed monoclonal antibodies may have led to selection of clones with intrinsic resistance to anti-EGFR directed monoclonal antibodies in the FU/FA plus panitumumab arm, which prevented the pure oxaliplatin re-induction to provide satisfactory efficacy, since the treated tumors appeared to be refractory to the other two drugs.^13^^,^^14^ Conversely, the application of treatment without anti-EGFR directed monoclonal antibodies (i.e. FU/FA alone) was likely associated with gradual depletion of resistant clones that may have developed during induction therapy.^14^^,^^15^ The clearly improved PFS of re-induction following FU/FA maintenance without panitumumab is therefore not surprising, but rather confirms the concept of re-challenge/re-induction strategies of anti-EGFR directed monoclonal antibodies following anti-EGFR directed monoclonal antibody free treatment intervals.^15^, ^16^, ^17^, ^18^

Secondly, the missing difference in the time to failure of strategy between both arms might indicate that the timepoint of adding panitumumab to treatment could play a subordinate role for the overall treatment efficacy.

By contrast, it might be argued, that any treatment choice can only guarantee (if at all) treatment for the following weeks, but not necessarily further treatment lines in the future.^19^, ^20^, ^21^ This knowledge of the underlying population effect might be the central reason why rather toxic and highly effective regimens, like EGFR-based combinations or FOLFOXIRI (even reducing options in later lines by using many substances upfront) became an established standard of care in mCRC.^22^, ^23^, ^24^, ^25^ With respect to the numerical advantage in OS observed in PanaMa, it might be speculated that the biologic sensitivity of the tumors to panitumumab in the standard of care arm (FU/FA alone maintenance) enabled the re-induction therapy to partly compensate the advantage of maintenance-panitumumab, but the population effect (not enough patients were able to follow this sequence of therapy) prohibited a numerical neutralization of OS.

In summary, our observations confirmed the primary analysis of PanaMa with significant prolongation of the primary endpoint PFS by the addition of Panitumumab to FU/FA maintenance treatment. In terms of the secondary endpoints, patients who, for example, do not tolerate or wish to receive maintenance therapy with panitumumab, might not necessarily face a survival disadvantage if the anti-EGFR antibody is re-introduced timely. By contrast, patients that received anti-EGFR antibodies during maintenance do not derive benefit from re-induction and a switch of the treatment strategy as next treatment line should be considered.^26^^,^^27^

Of note, CMS4 tumors appeared to have a significant OS benefit from maintenance with FU/FA plus panitumumab in our retrospective exploratory subgroup analysis. Although fibroblast-enriched, CMS4 tumors were formerly observed to respond exceptionally to EGFR inhibition when combined with Irinotecan.^28^ While oxaliplatin is suspected of antagonizing the antineoplastic effect of cetuximab in CMS4 tumors,^29^ no comparable studies exist for the interaction of oxaliplatin with panitumumab. A rather, albeit speculative, synergistic effect of oxaliplatin and panitumumab could therefore explain the survival advantage in CMS4 tumors.

No unexpected adverse events beside the known were observed in PanaMa. Of note, the rate of grade ≥3 events for the combination of FU/FA and panitumumab was higher during maintenance, but lower during re-induction therapy compared to FU/FA alone. Consistently with efficacy, it might be concluded that re-induction therapy somehow balanced the effects and consequences of therapy and that the advantage of less adverse events during maintenance therapy with FU/FA alone might be a short-lived observation and not enough reason to support avoiding panitumumab during maintenance therapy.

A limitation of PanaMa might be that only a subset (128 of 248 = 51.6%) of patients received re-induction in accordance with the trial protocol. Focused on the FU/FA plus panitumumab arm, these might be retrospectively regarded as clinically justified decisions by the individual investigators with respect to timely progression after FU/FA plus panitumumab maintenance strategy. In turn, it might be speculated that a higher rate of re-induction therapy in the FU/FA maintenance arm (78 of 123 patients received re-induction therapy = 63.4%) could have impacted positively on the long-term outcome, particularly OS, of this trial arm. Lastly, it should be noted that the sample size of PanaMa does not support a conclusive analysis of overall survival in the maintenance setting.

In summary, it is important to acknowledge that this trial –if the treatment schedule was respected-randomized rather a sequence of panitumumab use than a panitumumab-versus no-panitumumab question. Taking into account that re-induction therapy was apparently able to partly compensate the randomized PFS in this trial, both strategies explored in PanaMa might somehow be acceptable. A good reason to use FU/FA plus panitumumab as a standard of care maintenance regimen might be that a potential re-induction therapy with panitumumab cannot be guaranteed in the future whereas the immediate exposure in the maintenance therapy is a safe and effective treatment option.

## Contributors

**Conception and design:** D. P. Modest, S. Fruehauf, U. Graeven, K. Caca, S. Kasper, V. Heinemann, S. Stintzing, T. Trarbach.

**Administrative support:** D. P. Modest, A. Reinacher-Schick, S. Stintzing.

**Provision of study materials or patients:** D. P. Modest, S. Fruehauf, E. Goekkurt, A. Reinacher-Schick, S. Stintzing, T. Trarbach.

**Collection and assembly of data:** A. Stahler, D. P. Modest, M. Karthaus, S. Fruehauf, U. Graeven, L. Müller, L. Fischer von Weikersthal, K. Caca, A. Kretzschmar, A. Ballhausen, G. Sommerhäuser, A. H. S. Alig, E. Goekkurt, A. Jarosch, D. Horst, A. Reinacher-Schick, S. Kasper, V. Heinemann, S. Stintzing.

**Data analysis and interpretation:** A. Stahler, D. P. Modest, M. Karthaus, S. Fruehauf, U. Graeven, L. Müller, L. Fischer von Weikersthal, K. Caca, A. Kretzschmar, A. Ballhausen, G. Sommerhäuser, A. H. S. Alig, S. Held, E. Goekkurt, A. Jarosch, D. Horst, A. Reinacher-Schick, S. Kasper, V. Heinemann, S. Stintzing, T. Trarbach.

**Verification of the underlying data:** A. Stahler, S. Held, S. Stintzing, D. P. Modest.

**Manuscript writing**: All authors.

**Final approval of manuscript:** All authors.

**Accountable for all aspects of the work:** All authors.

## Data sharing statement

With respect to the clinical trial, Randomized Phase 2 study for evaluation of efficacy and safety of maintenance treatment with 5-FU/FA plus panitumumab versus 5-FU/FA alone after prior induction treatment with mFOLFOX6 plus panitumumab and re-induction with mFOLFOX6 plus panitumumab in case of progression for first-line treatment of patients with metastatic colorectal cancer” (PanaMa), sponsor code AIO-KRK-0212, EudraCT-No. 2012-005422-30, AIO-Studien-gGmbH acting as the legal sponsor is committed to provide information about its results to researchers with the goal of facilitating scientific progress. Information that will be considered for disclosure includes individual participant data that underlie the results reported in this article (text, tables, figures, and appendices). Additionally, study protocol and statistical analysis plan can be made available. All data shared must be anonymised to protect the privacy of the patients who participated in the trial, in accordance with applicable laws and regulations and in compliance with the International Council for Harmonisation and Good Clinical Practice (ICH/GCP). Researchers should provide a scientifically sound proposal directed to “info@aio-studien-ggmbh.de” for approval to gain access to the requested data. Shared data are only to be used to achieve aims of the approved proposal.

## Declaration of interests

A. Stahler: Advisory role: Bristol Myers Squibb, NovoCure, Takeda. Honoraria: Roche, Servier, Taiho Pharmaceutical, Amgen, Merck KGaA. Reimbursement for travel, accommodations, expenses: Amgen, Roche, Lilly, Pfizer, Servier.

M. Karthaus: Reimbursement for travel, accomodations, expenses: Amgen.

S. Fruehauf: Stock options: AbbVie, Bristol Myers Squibb/Pfizer, Johnson & Johnson/Janssen, Merck.

U. Graeven: Funding for the present manuscript: Amgen. Honoraria: Amgen, Roche, Merck, MSD, Boehringer Ingelheim, Sanofi.

A. Ballhausen: Grants: Amgen, AstraZeneca, Merck. Honoraria: Amgen, AstraZeneca, Merck. Reimbursement for travel, accomodations, expenses: Amgen. Leadership: Steering Committee member AIO working group for molecular and translational oncology within the German Cancer Society. Stock: BioNTech SE.

A. H. S. Alig: Honoraria: Amgen, MSD, Merck KGaA, Servier, Pfizer, Pierre-Fabre, Roche, BMS. Reimbursement for travel, accomodations, expenses: Nordic, Servier, Merck KGaA, MSD, Pfizer, Pierre-Fabre, Roche, Amgen, Daiichi Sankyo.

A. Reinacher-Schick: Advisory role: Abbvie, Amgen, Boehringer Ingelheim, Daiichi-Sankyo, Janssen-Cilag, Roche, Merck Serono, BMS, MSD, AstraZeneca, Pierre Fabre, Servier. Honoraria: Amgen, Roche, Merck Serono, BMS, MSD, MCI Global, AstraZeneca. Reimbursement for travel, accomodations, expenses: Roche, Amgen, MSD, Pierre Fabre. Other (Study grant to institution): Roche, Ipsen. Other (Research grants to institution): Roche, Celgene, Ipsen, Amgen, Alexion Pharmaceuticals, AstraZeneca, Lilly, Servier, AIO-Studien-gGmbH, Rafael Pharmaceutics, Erytech Pharma, BioNTech.

S. Kasper: Grants: Roche, BMS, Amgen, Lilly. Consulting Fees: BMS, MSD, Roche, Novartis, Merck Healthcare, Amgen, Servier, Lilly, AstraZeneca. Honoraria: Bristol Myers Squibb, MSD Oncology, AstraZeneca, Merck Serono, Amgen, Servier, Lilly, Onkowissen. Reimbursement for travel, accomodations, expenses: MSD, Lilly, Amgen, Roche. Participation on a data safety monitoring board: University Hospital Cologne.

S. Stintzing: Consulting Fees: Amgen, AstraZeneca, CV6, Bayer, BMS, Daiichi-Sankyo, ESAI, Leo-Pharma, Lilly, Merck KGaA, MSD, Pierre-Fabre, Roche, Sanofi, Servier, Taiho, Takeda. Honoraria: Amgen, AstraZeneca, Bayer, BMS, Daiichi-Sankyo, ESAI, ISOFOL, Leo-Pharma, Lilly, Merck KGaA, MSD, Pierre-Fabre, Roche, Sanofi, Servier, Taiho, Takeda. Reimbursement for travel, accomodations, expenses: Amgen, AstraZeneca, Bayer, BMS, Daiichi-Sankyo, ESAI, Leo-Pharma, Lilly, Merck KGaA, MSD, Pierre-Fabre, Roche, Sanofi, Servier, Taiho, Takeda. Advisory role: Amgen, AstraZeneca, CV6, Bayer, BMS, Daiichi-Sankyo, ESAI, Leo-Pharma, Lilly, Merck KGaA, MSD, Pierre-Fabre, Roche, Sanofi, Servier, Taiho, Takeda. Leadership: AIO.

T. Trarbach. Funding for the present manuscript: Amgen.

D. P. Modest: Grants: Amgen, Servier. Consulting Fees and advisory boards: Servier, Amgen, Takeda, Regeneron, IKF, Taiho, BMS, MSD, Bicara, Rottapharm, Neoconnect, 21up, Digimed, AstraZeneca, AIO gGmbH, PierreFabre, Jörg Eickeler, Incyte, Noggo, Lilly, Doktorflix, COR2ED. Honoraria: Servier, Amgen, Takeda, Regeneron, IKF, Taiho, BMS, MSD, Bicara, Rottapharm, Neoconnect, 21up, Digimed, AstraZeneca, AIO gGmbH, PierreFabre, Jörg Eickeler, Incyte, Noggo, Lilly, Doktorflix, COR2ED. Reimbursement for travel, accomodations, expenses: Servier, Amgen. No other potential conflicts of interest were reported.

## Appendix B Supplementary data

PanaMa study protocol
