# Supplementary material for: Panitumumab plus 5-fluorouracil and folinic acid or 5-fluorouracil and folinic acid alone as maintenance therapy in RAS wild-type metastatic colorectal cancer (PanaMa, AIO KRK 0212): final efficacy analysis of a randomised, open-label, phase 2 trial
Source: eClinicalMedicine. 2024 Dec 16;79:103004. doi: 10.1016/j.eclinm.2024.103004 (PMC11719858; doi:10.1016/j.eclinm.2024.103004)
Supplement: PanaMa study protocol [file mmc1.pdf]

PanaMa

Randomized Phase II study for evaluation of efficacy and safety of maintenance treatment with 5-FU/FA plus panitumumab vs. 5-FU/FA alone after prior induction treatment with mFOLFOX6 plus panitumumab and re-induction with mFOLFOX6 plus panitumumab in case of progression for first-line treatment of patients with metastatic colorectal cancer

**Short title:**	PanaMa
**Sponsor:**	AIO-Studien-gGmbH
**Coordinating investigator:**<br>(for Germany: LKP)	Prof. Dr. med. Dominik Paul Modest
**Coordinating study manager:**<br>(Sponsor)	Helge Schröder, Dipl. Biol.
**Data Management:**<br>**Monitoring:**<br>**Pharmacovigilance:**	ClinAssess GmbH<br>Birkenbergstr. 82<br>51379 Leverkusen
**Statistics:**	ClinAssess GmbH<br><br>Prof. Dr. Uwe Pichlmeier (Consultant)
**EudraCT no.:**	2012-005422-30
**Protocol identification number:**	AIO-KRK-0212
**Protocol version:**	FINAL Version 9.0, 02-Feb-2021

Confidentiality

The contents of the protocol are confidential and may neither be communicated verbally nor in writing without the agreement of the study sponsor.

CONTACT ADDRESSES

**Sponsor**	Dr. Aysun Karatas<br>AIO Studien gGmbH<br>Kuno-Fischer-Str. 8<br>14057 Berlin
**Coordinating Investigator and Steering Committee Chair**	Prof. Dr. med. Dominik Paul Modest<br>Medizinische Klinik m.S. Hämatologie, Onkologie und Tumorimmunologie CVK<br>Augustenburger Platz 1<br>13353 Berlin<br>Phone:030 450-553834<br>Fax: 030 450-553959<br><br>E-Mail: dominik.modest@charite.de
**Assistant to Coordinating Investigator and Steering Committee Chair**	Dr. med. Tanja Trarbach<br>Zentrum für Tumorbiologie und Integrative Medizin<br>Friedrich-Paffrath-Str. 100<br>26389 Wilhelmshaven<br>Tel.: 04421-892480<br>Fax.: 04421-892484<br>tanja.trarbach@klinikum-whv.de
**CRO**	ClinAssess GmbH<br>Birkenbergstr. 82<br>51379 Leverkusen<br>Tel.: +49 (0) 2171 / 36 336 0<br>Fax: +49 (0) 2171 / 36 336 55
**Statistics**	ClinAssess GmbH<br>Prof. Dr. Uwe Pichlmeier (Consultant)
**Investigational Medicinal Product**	Panitumumab
**Drug Supply**	Amgen (Panitumumab only)
**Protocol Committee**	Dr. Tanja Trarbach<br>Prof. Dr. Uwe Pichlmeier<br>Prof. Dr. Dominik Modest<br>Prof. Dr. Stefan Kasper<br>Prof. Dr. Sebastian Stintzing<br>Dr. Nicole Prasnikar

APPROVAL OF THE PROTOCOL

Dr. Aysun Karatas – Representative of the Sponsor

Signature — Date (DD Month YYYY): 02. Feb. 2021

Prof. Dr. Dominik Paul Modest – Coordinating Investigator (for Germany: LKP)

Signature — Date (DD Month YYYY): 2. 2. 2021

Swantje Held – Statistician, ClinAssess

Signature — Date (DD Month YYYY)

Dr. Burkhard Deuß – Representing the CRO

Signature — Date (DD Month YYYY)

APPROVAL OF THE PROTOCOL

Dr. Aysun Karatas – Representative of the Sponsor

Signature	Date (DD Month YYYY)

Prof. Dr. Dominik Paul Modest – Coordinating Investigator (for Germany: LKP)

Signature	Date (DD Month YYYY)

Swantje Held – Statistician, ClinAssess

	02.02.2021
Signature	Date (DD Month YYYY)

Dr. Burkhard Deuß – Representing the CRO

	02.02.2021
Signature	Date (DD Month YYYY)

INVESTIGATOR'S AGREEMENT

I have read the attached protocol entitled

"PanaMa Randomized phase II study for evaluation of efficacy and safety of maintenance treatment with 5-FU/FA plus panitumumab vs. 5-FU/FA alone after prior induction treatment with mFOLFOX6 plus panitumumab and re-induction with mFOLFOX6 plus panitumumab in case of progression for first-line treatment of patients with metastatic colorectal cancer",

and agree to abide by all provisions set forth therein.

I agree to comply with the International Conference on Harmonization Tripartite Guideline on Good Clinical Practice, all applicable national regulations as well as the requirements of the appropriate Institutional Review Board/Independent Ethics Committee and any other institutional requirements.

I agree to ensure that the confidential information contained in this document will not be used for any purpose other than the evaluation or conduct of the clinical investigation without the prior written consent of the study sponsor.

______________________________ ______________________________

Signature Date (DD Month YYYY)

____

Investigator

____

Investigator's Institution

SYNOPSIS

Protocol no.	AIO-KRK-0212
Protocol version (Date)	FINAL Version 9.0 (02-Feb-2021)
Title	PanaMa
Detailed title	Randomized phase II study for evaluation of efficacy and safety of maintenance treatment with 5-FU/FA plus panitumumab vs. 5-FU/FA alone after prior induction treatment with mFOLFOX6 plus panitumumab and re-induction with mFOLFOX6 plus panitumumab in case of progression for first-line treatment of patients with metastatic colorectal cancer
EudraCT no.	2012-005422-30
National Coordinating investigator	Prof. Dr. med. Dominik Paul Modest<br>Medizinische Klinik m.S. Hämatologie, Onkologie und Tumorimmunologie CVK<br>Augustenburger Platz 1<br>Phone: 030 450-553834<br>Fax: 030 450-553959<br>13353 Berlin
Sponsor	AIO-Studien-gGmbH
Translational research committee	Prof. Dr. Stefan Kasper, Prof. Dr. Dominik Modest, Prof. Dr. Sebastian Stintzing, Dr. Tanja Trarbach.
Quality of life committee	Dr. T. Trarbach
Study design	Phase II, randomized, multi-center, open-label, parallel-group
Start date	Q2 2014
Duration of study	Duration of accrual — Approx. 84 months<br><br>Final analysis of primary study endpoint with 218 events: — Approx. 88 months after start of enrollment<br><br>End of FU (observation period of at least 24 months after randomization for each patient): — Approx. 108 months after start of enrollment<br><br>End of study: — Approx. 24 months after last randomization
Total number of centers	approx. 95
Study population	Patients with metastatic colorectal cancer (wild-type RAS) in palliative first-line therapy
Primary objective	To assess the efficacy of panitumumab plus 5-FU/ FA as maintenance after an induction treatment of 12 weeks with mFOLFOX6 plus panitumumab in the first-line treatment of RAS wild-type metastatic colorectal cancer patients compared to 5-FU/ FA maintenance alone in terms of progression-free survival.
Secondary objectives	To compare maintenance arms with respect to:<br>• Time from randomization until failure of treatment strategy (death/ progression)<br>• Progression-free survival of re-induction<br>• Objective response after 12 weeks of induction chemotherapy<br>• Objective best response during maintenance and re-induction<br>• Overall survival measured from time of randomization and from time of registration

	• Safety<br>• Health and skin related Quality of life
Exploratory objectives	• Translational research parameters as defined in the respective section<br>• Central review of CT/MRI scans<br>• Depth of response (during induction and maintenance therapy)
Planned sample size	Approx. 400 patients will be enrolled to reach the planned number of 272 randomizations
Inclusion criteria	• Signed written informed consent<br>• Male or female ≥ 18 years of age<br>• Histologically proven metastatic colorectal cancer<br>• Geno- or phenotypically excluded dihydropyrimidine dehydrogenase (DPD) deficiency (by genotyping of the DPD-coding gene or by uracil plasmalevel measurement)<br>• Molecular testing showing RAS wild-type in colorectal carcinoma cells<br>• Life expectancy > 12 weeks<br>• At least one measurable lesion according to RECIST 1.1<br>• Adequate bone marrow, liver, kidney, organ and metabolic function<br>○ Bone marrow function<br>○ leukocyte count ≥ 3.0 × 10^9/L<br>○ ANC ≥ 1.5 x 10^9/L<br>○ platelet count ≥ 100 × 10^9/L<br>○ hemoglobin ≥ 9 g/dL or 5.59 mmol/L (may be transfused or treated with erythropoietin to maintain/ exceed this level)<br>○ Hepatic function<br>○ Total bilirubin ≤ 1.5 × UNL<br>○ ALT and AST ≤ 2.5 × UNL (or ≤ 5 × UNL in presence of liver metastases)<br>○ AP ≤ 5 × UNL<br>○ Renal function<br>○ Creatinine clearance ≥ 50 mL/ according to Cockroft-Gault formula or serum creatinine ≤ 1.5 × UNL<br>○ Metabolic function<br>○ Magnesium ≥ lower limit of normal<br>○ Calcium ≥ lower limit of normal<br>• ECOG performance status 0 - 1<br>• Women of child-bearing potential must have a negative pregnancy test
Exclusion criteria	• Previous treatment for colorectal cancer in the metastatic setting with the exception that patients with urgent need of immediate treatment (high tumor load, symptoms) may have received one cycle of any FOLFOX regimen (no capecitabine!) in case of yet unconfirmed RAS status.<br>• Previous EGFR-targeting therapy<br>• < 6 months after end of adjuvant therapy (previous chemoradiation for rectal cancer is accepted for inclusion into the trial and does not account as adjuvant therapy)

	• Known brain metastases unless adequately treated (surgery or radiotherapy) with no evidence of progression and neurologically stable off anticonvulsants and steroids<br>• Chronic inflammatory bowel disease<br>• Peripheral neuropathy ≥ NCI-CTCAE V 4.03 grade 2<br>• Other previous malignancies with the exception of a history of previous curatively treated basal cell carcinoma of the skin or pre-invasive carcinoma of the cervix or other curatively treated malignant disease without recurrence after at least 5 years of follow-up<br>• Significant disease that, in the investigator's opinion, would exclude the patient from the study<br>• History of cardiac disease; defined as:<br>o Congestive heart failure > New York Heart Association (NYHA) class 2<br>o Active coronary artery disease (myocardial infarction more than 6 months prior to start of study treatment is allowed)<br>o Cardiac arrhythmias requiring anti-arrhythmic therapy (beta-blockers or digoxin are permitted)<br>o Uncontrolled hypertension (defined as blood pressure ≥ 160 mmHg systolic and/or ≥ 90 mmHg diastolic on medication)<br>• Patients with interstitial lung disease, e.g. pneumonitis or pulmonary fibrosis or evidence of interstitial lung disease on baseline chest CT scan<br>• Known HIV, hepatitis B or C infection<br>• Known hypersensitivity reaction to any of the study components<br>• Radiotherapy, major surgery or any investigational drug 21 days before registration<br>• Pregnancy or lactation or planning to be pregnant during treatment and within 6 months after the end of treatment<br>• Subject (male or female) is not willing to use highly effective methods of contraception (per institutional standard) during treatment and for at least an additional 6 months after the end of treatment<br>• Known alcohol or drug abuse<br>• Any condition that is unstable or could jeopardize the safety of the patient and his compliance in the study
Treatment scheme	<u>Induction chemotherapy</u><br>6 cycles mFOLFOX6 plus panitumumab for 12 weeks<br><br>Panitumumab 6mg/kg BW<br><br>mFOLFOX6:<br>85 mg/m² Oxaliplatin 2h d1<br>400 mg/m² folinic acid 2h d1<br>2400mg/m² 5-FU over 46 h d1 -2<br>Q2w<br><br><u>Maintenance</u><br>Patient with CR, PR and SD after 12 weeks of induction treatment, will be randomized in a 1:1 ratio to receive either 5-FU/FA +

	panitumumab q2w (arm A) or 5-FU/FA alone q2w (arm B) until tumor progression.<br>Patient with curative resection within 12 weeks of induction therapy do not qualify for randomization.<br><u>Re-induction:</u><br>After tumor progression, a reinduction with mFOLFOX6 plus panitumumab will be started and patients will receive this regimen until tumor progression<br><br><u>Concomitant therapy:</u><br>Prophylactic management program for panitumumab-related acute and late skin toxicities (see section 6.5.2, 6.5.3)
Primary parameter	Progression-free survival during maintenance therapy defined as time from randomization until disease progression or death, whatever occurs first.
Secondary parameters	• Time from randomization until failure (death/ progression) of treatment strategy<br>• Progression-free survival of re-induction<br>• Objective response after 12 weeks of induction chemotherapy<br>• Objective best response during maintenance and re-induction<br>• Overall survival measured from time of randomization and from time of registration<br>• Safety<br>• Health and skin related Quality of life
Exploratory parameters	Translational research analysis in tumor tissue, circulation tumor cells, circulating tumor DNA, and blood cells. These investigations will include DNA, RNA, immunohistochemistry, FISH, Sequencing from tumor/or blood cells as well as evaluations of laboratory markers (tumor markers).<br><br>Central review of CT/MRI scans for resectability, volumetry and further related parameters (i.e. depth of response etc.)

Study procedures	After the initial screening procedure, eligible patients will be registered for study participation.<br><br>The patient receives chemotherapy consisting of 6 cycles mFOLFOX6 plus panitumumab every 2 weeks. Patients showing CR, PR or SD after induction therapy and qualifying for subsequent maintenance treatment and re-induction treatment with all potential drug components, will be randomized to receive a maintenance regimen of 5-FU/FA + panitumumab or 5-FU/FA alone until tumor progression.<br><br>After tumor progression a reinduction with mFOLFOX6 plus panitumumab will be started and patients will receive this regimen until tumor progression.<br><br>Tumor assessments will be performed 12 weeks after treatment start with induction therapy and every 8 weeks during maintenance therapy and re-induction.<br><br>All patients will have an end of treatment visit 4 weeks (+ 7 days) after the last dose of the study agent. Thereafter, all patients will be followed up for survival every 3 months.
Randomization procedure	Permuted block randomization will be applied to guarantee balanced group numbers throughout enrollment period. To increase homogeneity between treatment arms, randomization will be stratified by<br>1. Response to induction therapy at time of randomization (CR/PR vs. SD)<br>2. Prior oxaliplatin-containing adjuvant therapy (yes vs. no)<br>3. Planned starting dose of panitumumab for maintenance therapy, if patient will be assigned to arm A (full dosage vs. reduced dosage)<br><br>Randomization will be performed in the subgroup of patients achieving CR, PR or SD 12 weeks after start of induction therapy qualifying for maintenance treatment and re-induction treatment with all potential drug components.
Statistical considerations	
Sample size calculation	With a total number of 218 events (progressions or death, whichever occurs first), a logrank test for testing superiority of progression-free survival with a 10% one-sided significance level will have 80% power to reject the null-hypothesis if the true median progression-free survival times in patients treated with maintenance alone and maintenance plus panitumumab are 7.5 and 10 months, respectively. A total of approx. 400 patients eligible for induction therapy should be accrued for randomisation of 272 patients needed to reach the required number of events.
Planned interim analysis	No confirmatory interim analyses for efficacy with the aim to stop the trial prematurely are foreseen within this study protocol.

INFORMATION TO BE PROVIDED REGARDING SAES/PREGNANCY

In the case of a serious adverse event (SAE) or pregnancy, the following person must be contacted by fax within 24 hours:

ClinAssess GmbH Safety
Birkenbergstr. 82
51379 Leverkusen

Fax: +49 (0) 2171 / 36 336 55

FLOW CHART: SCHEDULE OF ASSESSMENTS DURING THE STUDY

	**Screening**		**Treatment phase (During induction phase, maintenance and re-induction)**		**End of treatment**	**Follow-up[16]**
	Within 21 days prior to start of therapy	Within 7 days prior to start of therapy	Day 1 of each cycle	12 weeks after start of induction therapy, every 8 weeks during maintenance and re-induction	4 weeks after last chemotherapy application	Every 3 months
Visit window [days]			±2	± 7	+ 7	± 7
Informed Consent[1]	x					
In- / Exclusion Criteria	x					
Demographics	x					
Medical History[2]	x					
Pregnancy Test (if applicable)[3]		x				
Physical examination		x	x		x	x
Vital signs[4]		x	x		x	
Body weight and height[5]		x	x		x	x
ECOG performance status		x	x		x	x
12 lead ECG[6]	x			(x)[15]	(x)[15]	
Hematology[7]		x	x		x	
Clinical chemistry[8]		x	x		x	
CEA, CA 19-9 measurement		x	x		x	
Tumor assessment (CT/ MRI) thorax/ abdomen[9]	x			X		(x)[17]
Skin toxicity[10]		x[14]	Continuous			x[18]
Adverse events[10]		x[14]	Continuous			x[18]
Confirming *RAS* status[11]	x					
Translational analyses	x[12]			x[20] (two timepoints)	x[20]	
Quality of life[13]	x		day 1 of first and 2nd cycle (week 2 & week 4), After that day 1 of every 2nd cycle (Cycle 4 [week 8], cycle 6 [week 12], cycle 8 [week 16], etc.)		x	
Concomitant medication	x		Continuous			
Survival						x[19]

[1] Prior to the first study-specific measures

[2] Medical History including information on dates and description of initial diagnosis of colorectal cancer and prior therapy as well as concurrent illnesses

[3] Applicable for women of childbearing potential. Serum β-HCG test within 7 days before the first dose of chemotherapy and panitumumab (to be repeated by urine test, if date of first result exceeds the 7-day window).

[4] Vital signs: Blood pressure, heart rate, body temperature

[5] Body height will be measured at screening only

[6] A 12-lead ECG will be performed at screening; during the treatment phase and for EOT only as clinically indicated

[7] Hematology: Leukocytes, ANC, erythrocytes, platelets, hemoglobin. In case a cycle of any FOLFOX regimen has been applied prior to enrollment due to urgent treatment need, the hematologic baseline values can be obtained from evaluation prior to or at that cycle to prevent bias of the assessment due to chemotherapy

[8] Clinical Chemistry: Sodium, potassium, calcium, magnesium, urea, alkaline phosphatase, AST, ALT, LDH, total bilirubin, serum creatinine, creatinine clearance (calculated by Cockroft Gault formula), total protein, albumin

[9] The baseline tumor assessment must be done within 21 days prior to treatment start. Tumor assessment (CT or MRI) will be done according to RECIST 1.1 and will comprise thorax and abdomen. Radionuclide bone scan/skeletal x-ray for suspected bone metastases, and cranial CT for suspected CNS metastases will only be performed if clinically indicated. During the treatment phase tumor assessments will be performed 12 weeks after start of induction therapy and every 8 weeks during maintenance and re-induction therapy with a visit window of +/- 7 days. For patients discontinuing maintenance or re-induction prior to documentation of disease progression, disease assessment will continue every 8 weeks (+/- 7 days) until disease progression is documented or other anti-cancer therapy is started, whichever comes first

[10] To be evaluated according to NCI-CTCAE version 4.03

[11] Only patients with documented wild-type *RAS* (exon 2 [codons 12/13], exon 3 [codons 59/61], and exon 4 [codons 117/146] of KRAS **and** NRAS) may be included, resulting from primary or metastatic tumor (tested anytime).

[12] Optional: exploratory analyses will be performed on paraffin-embedded archived tumor tissue and circulating tumor cells/ tumor DNA. The following will be collected during screening:

- 1 Tumor block (paraffinated)
- 1 PAXGene DNA-Tube (8,5ml)
- 1 PAXGene RNA-Tube (8,5ml)
- 1 EDTA-Tube (7ml)
- 1 Serum-Tube (5ml)
- 1 Streck Tube (for cell free DNA analysis) (9ml)

[13] Quality of life will be assessed using the EORTC QLQ-C 30, DLQI, FACT-EGFRI 18 and Skindex-16 questionnaires

[14] Current symptoms and/ or residual toxicities from prior therapy; all adverse events from date of informed consent are to be documented

[15] ECG during treatment and for end of treatment visit only if clinically indicated

[16] The first visit of the long-term follow-up period will take place three months after the EOT visit. Follow-up for the individual patient will continue until death or until 2 years after randomization of the last randomized patient (whichever occurs first). Every effort will be made to collect the exact date of death.

[17] During Follow-up, tumor assessments will only be documented at 8 week intervals for patients who discontinued treatment before progression of disease occurred.

[18] Adverse events present at the end of treatment will be followed-up until the event has resolved or stabilized

[19] Survival data and further anti-cancer therapy

- [20] Optional: exploratory analyses will be performed on whole blood and serum samples. Samples will be collected at up to 4 timepoints: On day 1 of induction therapy and day 1 of maintenance therapy, on end of maintenance therapy (i.e. day of PD diagnosis) as well as during the end of treatment visit.1 EDTA-Tube (7ml) and 1 Serum-Tube (5ml), 1 Streck Tube (for cell free DNA analysis) (9ml)

GLOSSARY OF ABBREVIATIONS

β-HCG	Beta human chorionic gonadotropin
AE	Adverse event
ALT (SGPT)	Alanine aminotransferase
AMG	Arzneimittelgesetz (German Drug Law)
ANC	Absolute neutrophil count
AST (SGOT)	Aspartate aminotransferase
BSA	Body surface area
BW	Body weight
CEA	Carcinoembryonic antigen
CI	Confidence interval
CNS	Central nervous system
CR	Complete response
CRC	Colorectal cancer
CRF	Case report form
CRO	Contract research organization
CT	Computer tomography
CTCAE	Common terminology criteria for adverse events
D	Day
DLQI	Dermatology Life Quality Index
DSMB	Data safety monitoring board
ECG	Electrocardiogram
ECOG	Eastern Cooperative Oncology Group
EGFR	Epidermal growth factor receptor
EORTC	European Organization for Research and Treatment of Cancer
EOT	End of treatment
FA	Folinic acid
5-FU	5-fluorouracil
GCP	Good Clinical Practice
GCP-V	GCP-Verordnung
GI	Gastrointestinal
HR	Hazard ratio
IC	Induction chemotherapy
ICH	International Conference on Harmonization
IEC	Independent ethics committee
IgG2	Immunoglobulin G2
IMP	Investigational medicinal product
IRB	Institutional review board
ITT	Intent to treat
IV	Intravenous
KRAS	Kirsten Rat sarcoma
NRAS	Neuroblastoma RAS viral oncogene homolog
LDH	Lactate dehydrogenase
LKP	Leiter der klinischen Prüfung (coordinating investigator)
mCRC	Metastatic colorectal cancer
MRI	Magnetic resonance imaging
MT	Mutant type
NCI	National Cancer Institute
OS	Overall survival
PCR	Polymerase chain reaction
PD	Progressive disease
PFS	Progression-free survival
PPS	Per protocol set

PR	Partial response
QLQ	Quality of life questionnaire
RAS	Rat Sarcoma
RECIST	Response Evaluation Criteria in Solid Tumors
rHuMAb	Recombinant humanized monoclonal antibody
SAE	Serious adverse event
SD	Stable disease
SmPC	Summary of product characteristics
SADR	Serious adverse drug reaction
SAP	Statistical Analysis Plan
SPF	Sun protection factor
SUSAR	Suspected unexpected serious adverse reaction
TGF	Transforming growth factor
UNL	Upper normal limit
VEGF	Vascular endothelial growth factor
WT	Wildtype

TABLE OF CONTENTS

1	BACKGROUND AND RATIONALE		19
1.1	**Background**	**19**	
1.1.1	Colorectal cancer	19	
1.1.2	EGFR inhibition in colorectal cancer	19	
1.1.3	Study drug panitumumab	20	
1.1.3.1	*Clinical efficacy of panitumumab*	*21*	
1.1.3.2	*Clinical experience - safety*	*22*	
1.2	**Study Rationale**	**23**	
2	OBJECTIVES OF THE STUDY		26
2.1	**Primary Objectives**	**26**	
2.2	**Secondary Objectives**	**26**	
2.3	**Exploratory Objectives**	**26**	
3	INVESTIGATIONAL PLAN		27
3.1	**Overview of Study Design and Dosing Regimen**	**27**	
3.1.1	Definition of Treatment Cycle and Duration	30	
3.1.2	Treatment Phase	30	
3.1.3	End of Treatment Visit	30	
3.1.4	Follow-Up Phase	30	
3.2	**Assignment of Patients to Treatment**	**31**	
3.3	**Centers**	**32**	
3.4	**Study Duration**	**32**	
4	SELECTION OF THE STUDY POPULATION		33
4.1	**Target Population**	**33**	
4.2	**Inclusion Criteria**	**33**	
4.3	**Exclusion Criteria**	**34**	
5	SCHEDULE OF ASSESSMENT AND PROCEDURES		35
5.1	**Study Assessments**	**35**	
5.1.1	Tumor Assessments	35	
5.1.2	Other Clinical Assessments	36	
5.1.3	Safety Assessments	38	
5.1.4	Laboratory Assessments	39	
5.1.5	Translational analysis	39	
5.1.6	Digital imaging	41	
5.2	**Study Procedures**	**42**	
5.2.1	Screening Procedures	42	

5.2.2 Treatment Phase 43
5.2.3 End of Treatment 44
5.2.4 Follow-Up 44
5.2.5 End of Study 44
5.3 Planned Treatment of the Patient after End of Treatment Phase 44
5.4 Removal of Patients from Treatment 44
5.5 Study Discontinuation 46
6 INVESTIGATIONAL PRODUCT 47
6.1 Investigational medicinal product (IMP) 47
6.2 Preparation and Administration of test drug panitumumab and chemotherapy 47
6.2.1 Preparation and Administration of panitumumab 47
6.2.1.1 Drug Name, Formulation and Storage 47
6.2.1.2 Route of Administration 48
6.2.1.3 Preparation of Study Drug 48
6.2.1.4 Compliance 49
6.2.2 Preparation and Administration of chemotherapy 50
6.2.2.1 Oxaliplatin 50
6.2.2.2 5-Fluorouracil 50
6.2.2.3 Folinic Acid 50
6.3 Dose Modifications 51
6.3.1 Dose Modifications for panitumumab 52
6.3.1.1 Interruption of panitumumab infusion 52
6.3.1.2 Panitumumab dosage adjustments 52
6.3.1.3 Criteria for withholding a dose of panitumumab 54
6.3.1.4 Criteria for re-treatment with panitumumab 54
6.3.1.5 Dose modification schedule 54
6.3.1.6 Panitumumab delayed or missed doses 56
6.3.2 Dose Modifications for chemotherapy 56
6.3.2.1 General aspects 56
6.3.2.2 Specific toxicities - hematologic toxicities 57
6.3.2.3 Specific toxicities – oxaliplatin induced toxicities 58
6.3.2.4 Discontinuation of therapy 59
6.4 Preconditions for starting a new treatment cycle 60
6.5 Concomitant and supportive medication and treatment 60
6.5.1 Pre-medication 60
6.5.2 Skin toxicities 61
6.5.2.1 General pre-emptive skin treatment 61
6.5.3 Preventive and treatment recommendations for specific clinical symptoms of the skin 62
6.5.4 Treatment of nausea/ vomiting 66

6.5.5 Treatment of diarrhea 66
6.5.6 Electrolyte management 66
6.5.7 Prevention and treatment of neutropenia 67
6.5.8 Prohibited Medications 67

7 ASSESSMENT OF SAFETY 68

7.1 Definitions of Adverse Events and Serious Adverse Events 68
7.1.1 Adverse Event 68
7.1.2 Adverse Drug Reaction 68
7.1.3 Serious Adverse Event 68
7.1.4 SUSAR 69

7.2 Reporting of SAEs 69

7.3 Reporting of SUSARs 70

7.4 Recording of Adverse Events 71

7.5 Laboratory Test Abnormalities 72

7.6 Pregnancy 72

7.7 Adverse Drug Reactions with Concomitant Medication 73

8 BIOSTATISTICAL ASPECTS 74

8.1 Trial Design and Hypotheses 74

8.2 Sample Size Calculation 74

8.3 Evaluation Categories for Patients 76
8.3.1 Full Analysis Population 76
8.3.2 Per Protocol Population 76
8.3.3 Safety Population 76

8.4 Methods of Statistical Analysis 77
8.4.1 General Statistical Considerations 77
8.4.2 Demographics and Baseline Characteristics 78
8.4.3 Efficacy Evaluation 78
8.4.4 Safety Evaluation 82

8.5 Interim and Final Analysis 83

9 DATA QUALITY ASSURANCE 83

10 STUDY COMMITTEES – DATA SAFETY MONITORING BOARD 84

11 ETHICAL ASPECTS 85

11.1 Declaration of Helsinki / Good Clinical Practice 85

11.2 Patient Information and Informed Consent 85

11.3 Independent Ethics Committees and Regulatory Authorities 86
11.3.1 Approval of the Study by the Regulatory Authority and Independent Ethics Committees 86

11.3.2 Notification of the Study 86
11.3.3 Obligation to Report and Document 86

12 CONDITIONS FOR MODIFYING THE PROTOCOL 86

13 STUDY DOCUMENTATION, CRFS AND RECORD-KEEPING 87

13.1 Investigator's Files / Retention of Documents 87

13.2 Source Documents and Background Data 87

13.3 Audits and Inspections 88

13.4 Electronic Case Report Forms (eCRF) 88

14 MONITORING THE STUDY 89

15 CONFIDENTIALITY OF TRIAL DOCUMENTS AND PATIENT RECORDS 89

16 STUDY REPORT AND PUBLICATION POLICY 90

17 APPENDICES 91

18 REFERENCES 103

PART I – STUDY DESIGN AND CONDUCT

1 Background and Rationale

1.1 Background

1.1.1 Colorectal cancer

Colorectal cancer (CRC) is one of the most frequent malignancies in developed countries and one of the leading causes of cancer-related deaths[1-3]. In Germany, about 65,000 people are currently diagnosed with CRC every year[2]. By the time of diagnosis a loco-regional spread of the disease is already detected in 36% of the patients, while about 20% of patients already present with metastatic disease at initial diagnosis[4].

For palliative first-line treatment of metastatic disease, chemotherapy regimens with 5FU/LV and oxaliplatin and/ or irinotecan have become standard of care as chemotherapy backbones in both, 1st and 2nd line therapy[5]. On the basis of a randomized phase II trial, it has been shown that the efficacy of FOLFIRI followed by FOLFOX6 at progression is similar to the vice-versa-sequence of FOLFOX6 followed by FOLFIRI in terms of progression-free (14.2 vs. 10.9 months, respectively, p=0.64) and overall survival (21.5 vs. 20.6 months, respectively; p=0.99), offering patients both options for a sequential combination treatment[6].

Even with the improvement in traditional chemotherapy made over the years, there remain limitations with this treatment approach. The substantial unmet needs in preventing and managing metastatic cancer may be addressed, at least in part, by better characterizing its pathogenesis and growth and developing more specifically acting agents. The emergence of molecularly targeted agents, such as those targeting the epidermal growth factor receptor (EGFR) and vascular endothelial growth factor (VEGF) represent a key treatment advance. In recent years, several studies have shown the efficacy of targeted therapy and led to the approval of the VEGF antibody bevacizumab and the EGFR antibodies panitumumab and cetuximab for treatment of metastatic colorectal cancer.

1.1.2 EGFR inhibition in colorectal cancer

EGFR is a cell surface protein that is overexpressed in many carcinomas. Several studies have shown that overexpression of EGFR is a poor prognostic factor in colon cancer (summarized in Yarom and Jonker, 2011[7]). The receptor is thought to drive tumor metastasis and proliferation by binding its ligands, which leads to dimerization, auto-phosphorylation and activation of the receptor and the subsequent activation of at least three downstream intracellular signaling pathways: the Ras-Raf-MAPK pathway, the PI3K-Akt pathway, and the protein kinase C-Jak/Stat pathway, all of which are thought to drive transcription of genes involved in tumor growth and survival[8].

Panitumumab and cetuximab are antibodies targeting EGFR. They appear to exert their anti-neoplastic effects by blocking ligand binding by the receptor thus preventing activation of the receptor and the downstream signaling pathways. The two monoclonal antibodies both have proven efficacy in large randomized studies on advanced colorectal cancer, either in 1st line or relapse treatment, and as single drug or combined with cytotoxic agents[9-13], leading to the market authorization of both drugs in this disease.

However, if one or more of the downstream effector molecules contains a mutation rendering that molecule constitutively active, receptor blockade may have little or no effect on tumor growth, as it has been demonstrated for KRAS, one of the molecules in the Ras-Raf-MAPK pathway. Data from multiple clinical trials of both panitumumab and cetuximab in colorectal cancer (CRC) have demonstrated that patients whose tumors contain activating mutations in the KRAS gene do not derive clinical benefit from antibody therapy and have significantly shortened survival compared to patients whose tumor expresses wild-type (wt) KRAS[12,14-16]. Based on the results of these and further studies, administering EGFR-targeting monoclonal antibodies to unselected metastatic colorectal cancer patients can no longer be considered the standard of care, as those agents will be ineffective in patients with activating mutations in KRAS[17,18].

Besides the well-known KRAS mutations, several other mutations were analyzed with respect to EGFR antibody response prediction, including BRAF, NRAS, PTEN, PI3K-AKT and single-nucleotide polymorphisms[16,19-21] The first analyses of these data revealed that mutations in these molecules may also have an impact on response rates. Analysis of a phase III panitumumab monotherapy study indicated that KRAS and NRAS mutations beyond KRAS exon 2 may be predictive of panitumumab efficacy[22]. A prospectively defined retrospective analysis of data from a randomized, multicenter phase 3 study (PRIME study) of panitumumab plus FOLFOX versus FOLFOX alone in subjects with previously untreated wild-type KRAS metastatic colorectal cancer indicated a statistically significant OS benefit in patients with wild-type *RAS* under treatment with panitumumab and FOLFOX versus treatment with FOLFOX alone wheras patients with any RAS mutations were unlikely to benefit from treatment with panitumumab and FOLFOX and BRAF mutation had no predictive value[23].

1.1.3 Study drug panitumumab

Panitumumab [pINN] (rHuMAb-EGFr; ABX-EGF) is a high affinity ($K_d = 5 \times 10^{-11}$ M) human IgG2 monoclonal antibody directed against human EGFR. Panitumumab blocks EGFR binding of the ligands EGF, TGFα, amphiregulin, betaregulin, epiregulin, and heparin-binding EGF. Autocrine or paracrine stimulation of EGFR by its ligands may have a critical role in the

progression of tumors expressing this receptor; thus, an antibody that blocks ligand binding to EGFR may inhibit tumor cell survival, proliferation, and metastasis.

For details on pre-clinical data, please refer to the current investigator's brochure.

1.1.3.1 Clinical efficacy of panitumumab

Panitumumab is being studied as a monotherapy and in combination with chemotherapy and/or radiotherapy in several clinical studies. Efficacy has been observed in various tumor types when given as both monotherapy and in combination with chemotherapy.

Efficacy results from a phase III, randomized (1:1), controlled, multicenter clinical trial of panitumumab (Study 20020408)[13] in 463 subjects with metastatic colorectal cancer who had disease progression during or after prior standard fluoropyrimidine, irinotecan, and oxaliplatin chemotherapy demonstrated that treatment with 2-weekly panitumumab plus best supportive care (BSC) increased progression-free survival relative to BSC alone ($p < 0.0001$). The rate of disease progression or death was reduced by approximately 43% in the panitumumab group (hazard ratio = 0.574, 95% CI: 0.473, 0.697). Twenty-two subjects (10%) in the panitumumab plus BSC group and none in the BSC alone group had an objective response by central review ($p < 0.0001$). Analysis of tumor samples from this study suggests that improvements of PFS and objective response rate are restricted to subjects with KRAS wt tumors[14]. 5 phase II studies (20025405, 20030167, 20030250, 20030194 and 20050216) confirmed the anti-tumor activity (measured by objective response rates) in subjects with mCRC who failed prior irinotecan and/ or oxaliplatin treatment.

Two pivotal large phase III studies (20050203 and 20050181)[10,11] on combinations with FOLFOX and FOLFIRI provide efficacy data on panitumumab in combination with chemotherapy as initial or second-line treatment in the mCRC setting. Both studies were open-label, randomized trials of panitumumab plus chemotherapy versus chemotherapy alone. FOLFOX and FOLFIRI were selected for these studies because they were among the standard of care in this setting when these trials were designed. The results demonstrated that in subjects with wt KRAS tumor status, panitumumab resulted in a statistically significant and clinically relevant improvement of PFS when combined with chemotherapy relative to chemotherapy alone. For first-line treatment with FOLFOX4 +/- panitumumab, PFS was increased from 8.0 to 9.6 months (HR 0.789, 95% CI 0.656, 0.971; log-rank test $p = 0.0234$)[10]. For second-line treatment with FOLFIRI +/- panitumumab, PFS was increased from 3.9 to 5.9 months (HR 0.732, 95% CI 0.593, 0.903; log-rank test $p = 0.0036$)[11]. In both phase III studies, the primary analysis of overall survival (OS) showed a strong trend towards improvement with panitumumab plus chemotherapy relative to chemotherapy alone in subjects with wt KRAS tumors (absolute difference in median OS of 4.2 months and 2.0 months in studies 20050203

and 20050181, respectively), but did not demonstrate a statistically significant difference between treatment arms. The incidence of subsequent anti-EGFR therapy was higher in subjects receiving chemotherapy alone compared with those receiving panitumumab plus chemotherapy; these imbalances between treatment arms may have influenced the analysis of OS.

In study 20050203, PFS and OS were worse in subjects with mt KRAS tumors in the panitumumab plus FOLFOX arm compared to the FOLFOX alone arm. In study 20050181, the addition of panitumumab to FOLFIRI had no positive or negative effect on PFS, OS or objective response rate in subjects with mt KRAS tumors. In subjects with wt KRAS tumors, panitumumab resulted in a higher objective response rate (i.e., confirmed CR or PR) when combined with chemotherapy relative to chemotherapy alone in study 20050203 (55% vs. 48%) and study 20050181 (35% vs. 10%).

For further details on efficacy data, please refer to the current investigator's brochure.

1.1.3.2 Clinical experience - safety

Safety analyses from clinical studies in subjects with mCRC (n = 2588) receiving panitumumab as monotherapy or combination therapy indicated that panitumumab is generally well tolerated.

In these subjects, skin-related toxicities were the most frequently reported adverse events (93% of subjects), with most events being mild to moderate.

Dermatologic toxicities include rash (45%), acneiform dermatitis (39%), pruritus (35%), erythema (30%), and dry skin (22%). Relatively few subjects (2%) permanently discontinued panitumumab due to dermatologic adverse events. Dermatologic toxicities were typically observed after initiation of panitumumab, with a median time to first integument toxicity (of any severity) of 10 days (95% CI: 8, 11). Severe skin toxicities developed in 34% of patients, and <1% of patients developed skin toxicities of NCI-CTCAE grade 4. Rare cases of Stevens-Johnson syndrome and toxic epidermal necrolysis occurred in the postmarketing phase.

Other common treatment-related adverse events (i.e., subject incidence ≥ 10%) included gastrointestinal toxicities (diarrhea (50%), nausea (41%), vomiting (27%), constipation (23%) and abdominal pain (23%)), constitutional symptoms (fatigue (37%) and fever (37%)), anorexia (27%) and paronychia (20%). Infusion reactions to panitumumab (defined as any reported allergic reaction, anaphylactic reaction, chills, fever, or dyspnea, occurring within 24 hours of the first dose that were not otherwise designated as either anaphylactic or allergic reaction) were infrequent (4% of subjects; < 1% severe); particularly considering that premedication was not mandated in study protocols.

More treatment-related adverse events occurred in the wild-type KRAS subset compared with the mutant KRAS subset, presumably due to the longer therapy. Subjects in the wild-type KRAS subset received a higher number of panitumumab infusions compared with subjects in the mutant subset (mean [median] 10.0 [8.0] and 4.9 [4.0], respectively). These adverse events were mainly skin toxicities (erythema, pruritus, dermatitis acneiform) likely reflecting the increased duration of exposure to panitumumab. No qualitative differences in overall adverse events were observed between the wild-type KRAS subset, the mutant KRAS subset and the overall population, however, treatment related grade 3 adverse events were reported for 25% of subjects in the wild-type KRAS subset compared with 12% of subjects in the mutant KRAS subset. 2% of wild-type KRAS subjects and 1% of mutant KRAS subjects withdrew for panitumumab-related events.

The clinical safety profile of panitumumab in combination with chemotherapy is based on results from 9 clinical studies with data from 1536 subjects, including 585 subjects who received panitumumab in combination with oxaliplatin-based chemotherapy. The safety data of the pivotal phase III trials[10] indicate a tolerable safety profile for panitumumab when administered in combination with oxaliplatin to subjects with wt KRAS mCRC as first-line treatment. In subjects treated with oxaliplatin, the rate of grade 3 and 4 adverse events and serious adverse events was slightly higher for panitumumab plus chemotherapy relative to chemotherapy alone, primarily due to toxicities known to be associated with panitumumab, such as rash, diarrhea, acneiform dermatitis and hypomagnesemia. However the rate of discontinuation was similar between treatment arms, suggesting that these events were clinically manageable.

Please refer to the current version of the panitumumab Investigator’s Brochure as well as the updated safety information contained in the Investigational New Drug safety letters for further updates.

1.2 Study Rationale

Palliative treatment for metastatic colorectal cancer, though not intending to cure, should focus on both length and quality of life.

Combination chemotherapy plus anti-EGFR antibody is approved for first-line therapy in metastatic colorectal cancer in KRAS wild type patients. However, continuous therapy until tumor progression is problematic in terms of toxicity and quality of life, and its necessity for maximum antineoplastic effect and patient benefit remains to be proven. Therefore, concepts to optimize or reduce therapeutic agents to a minimum while retaining efficacy are needed. The concept of limiting the number of chemotherapy cycles and continuing with an intensity-

reduced maintenance treatment including the EGFR antibody seems to be most promising, if the induction combination therapy can be administered at full dose and without any delay, and if the remaining toxicity of the maintenance treatment can be specifically addressed.

Palliative treatment with oxaliplatin-containing regimens is standard of care. However, the cumulative neurotoxicity associated with oxaliplatin often requires patients to discontinue while still responding to therapy.

It has been investigated in several trials, whether oxaliplatin-based regimens can be used in a partial stop-and-go fashion (induction with an oxaliplatin-based regimen, maintenance without oxaliplatin and re-introduction upon progression) or in a complete stop-and-go fashion (oxaliplatin-based regimen with therapy free interval) in order to reduce the toxic side effects of therapy.

The OPTIMOX2 trial directly compared a partial and a complete stop-and-go strategy, showing longer duration of disease control, PFS and OS in the partial stop-and-go arm, hence favoring the partial stop-and-go strategy[24]. The COIN trial did not show non-inferiority of intermittent compared with continuous chemotherapy in terms of OS[25].

The OPTIMOX1 trial has shown that a short induction with FOLFOX7 followed by maintenance therapy with 5-FU alone was better tolerated than and achieved a similar efficacy as continuous administration of FOLFOX4[26]. The median PFS was 9 months for continuous chemotherapy (arm A) and 8.7 months for induction/ maintenance therapy (arm B). Overall survival was similar for both arms with 19.3 months (arm A) and 21.2 months (arm B) (HR = 0.93; 95% CI: 0.72, 1.11; P = 0.49). Objective response rates were similar between treatment arms (58.5% arm A; 95% CI: 54.5%, 62.5%; 59.2% arm B; 95% CI: 55.2%, 63.2%; P = not significant). During early treatment, the tolerability and toxicity profiles of the two arms were similar. However, the risk of developing a grade 3 or 4 toxicity was greatly reduced during maintenance therapy, when patients did not receive oxaliplatin. The results of the study are consistent with the proposition that, for most patients, a limited number of cycles of FOLFOX are sufficient to achieve the full clinical benefits of oxaliplatin treatment. In a successor study, OPTIMOX2, it could be shown that the majority of patients could resume FOLFOX after progression on maintenance therapy[24].

These trials show that a partial stop-and-go strategy for oxaliplatin-based regimens is feasible and better tolerated than continuous chemotherapy with oxaliplatin for palliative therapy of metastatic colorectal cancer.

A recent trial presented at the 2012 ASCO investigated the use of targeted therapy during maintenance therapy. The DREAM study showed significantly improved duration of PFS when

bevacizumab and erlotinib were used for maintenance therapy compared to bevacizumab alone[27]. This trial suggests that targeted therapy is beneficial during maintenance treatment.

As the anti-EGFR antibody panitumumab is approved for first-line therapy in metastatic colorectal cancer in KRAS wild-type patients, this study aims to investigate whether addition of a targeted agent, i.e., panitumumab, to a partial stop-and-go strategy of mFOLFOX6, in analogy to the OPTIMOX-concept, can reduce the oxaliplatin component to an effective minimum, while maintaining the maximum clinical benefit and improving the quality of life.

Although it has been demonstrated that patients with mutant KRAS fail to respond to anti-EGFR therapy, not all patients with wild-type KRAS respond. As mentioned in the introduction, several other potential biomarkers have been investigated for predictability of response, and accordingly wild-type RAS beyond exon 2 (not only KRAS exon 2 wild-type) as probable prerequisite of any treatment with panitumumab in combination with FOLFOX[22,23] will be an inclusion criterion for the study. However, further investigations are needed to improve the ability to predict response to EGFR directed therapy. To further evaluate potential correlations between the regimen and EGFR expression as well as to explore potential predictive markers of response and to collect additional information on *RAS* and BRAF mutations, exploratory analyses will be included in this study and will be performed on archived tumor tissue and serum samples. These analyses are of great importance in light of the above cited recent retrospective analyses with regard to the importance of testing the RAS mutation status beyond exon 2 as probable prerequisite of any treatment with panitumumab in combination with FOLFOX[22,23].

2 Objectives of the Study

2.1 Primary Objectives

To assess the efficacy of panitumumab plus 5-FU/ FA as maintenance after an induction treatment of 12 weeks with mFOLFOX6 plus panitumumab in the first-line treatment of RAS wild-type metastatic colorectal cancer patients compared to 5-FU/ FA maintenance alone.

Efficacy will be compared by means of progression-free survival during maintenance therapy defined as time from randomization until disease progression or death, whichever occurs first. Randomization will take place in the subgroup of patients showing CR, PR or SD 12 weeks after start of induction therapy and qualifying for maintenance treatment and re-induction treatment with all potential drug components.

Randomization after 12 weeks of induction therapy and excluding the time of induction treatment from derivation of progression-free survival time will increase the power to detect any potential difference between maintenance plus/minus panitumumab since induction therapy does not contribute to any treatment effect.

2.2 Secondary Objectives

To compare maintenance arms with respect to:

- Time from randomization until failure of treatment strategy (death/ progression)
- Progression-free survival from re-induction
- Objective response after 12 weeks of induction chemotherapy
- Objective best response during maintenance and re-induction
- Overall survival measured from time of randomization and from time of registration
- Safety
- Health and skin related Quality of life

2.3 Exploratory Objectives

- Translational research parameters including assessments in tumor tissue (mutations, expressions, etc. related to CRC or used therapy (e.g., 5-FU/LV, oxaliplatin, panitumumab).
- Assessment of circulating markers such as tumor markers or circulating tumor cells from collected blood samples
- Any exploratory marker can/will be correlated to clinical data
- Central evaluation of CT/MRI scans obtained between registration and final progression following re-induction therapy.

- CT/MRI scans can be evaluated for resectability, volumetry and for exploratory information such as depth of response, time to deepest response.
- All data obtained during central evaluation of CT/MRI scans will be correlated to clinical data.

3 Investigational Plan

3.1 Overview of Study Design and Dosing Regimen

This is a phase II, randomized, multi-center, open-label, parallel-group study to evaluate the progression-free survival during maintenance therapy.

Eligible patients will be treated within a 12-week induction therapy. Those patients achieving CR/PR or SD at 12 weeks and qualifying for maintenance treatment and re-induction treatment with all potential drug components, will be randomized in a ratio of 1:1 to receive chemotherapy plus panitumumab (arm A) or chemotherapy alone (arm B) during maintenance. In case of progression, re-induction treatment will be started.

Induction therapy

- **6 cycles of mFOLFOX6 induction therapy plus panitumumab**

  Panitumumab at a dose of 6 mg/kg prior to administration of chemotherapy
  Oxaliplatin 85 mg/m^2 over 2 hours on day 1
  Folinic acid 400 mg/m^2 over 2 hours on day 1
  5-FU 2400mg/m^2 46h continuous infusion day 1 - day 2
  Repeat on day 15

Arm A:

- **Maintenance therapy**

  Panitumumab at a dose of 6 mg/kg prior to administration of chemotherapy
  Folinic acid 400 mg/m^2 over 2 hours on day 1
  5-FU 2400mg/m^2 46h continuous infusion day 1 - day 2
  Repeat on day 15

- **Re-induction upon progression**

  Panitumumab at a dose of 6 mg/kg prior to administration of chemotherapy
  Oxaliplatin 85 mg/m^2 over 2 hours on day 1
  Folinic acid 400 mg/m^2 over 2 hours on day 1
  5-FU 2400mg/m^2 46h continuous infusion day 1 - day 2

Repeat on day 15

Arm B:

- **Maintenance therapy**

  Folinic acid 400 mg/m^2 over 2 hours on day 1

  5-FU 2400mg/m^2 46h continuous infusion day 1 - day 2

  Repeat on day 15

- **Re-induction upon progression**

  Panitumumab at a dose of 6 mg/kg prior to administration of chemotherapy

  Oxaliplatin 85 mg/m^2 over 2 hours on day 1

  Folinic acid 400 mg/m^2 over 2 hours on day 1

  5-FU 2400mg/m^2 46h continuous infusion day 1 - day 2

  Repeat on day 15

Re-induction therapy will be administered until progression of disease. Tumor assessments will be performed 12 weeks after start of induction therapy and every 8 weeks during maintenance and re-induction. Objective response will be evaluated according to RECIST version 1.1 (see appendix 4). The same assessment method (i.e. CT, MRI) should be used throughout the study for each subject. In case of unacceptable toxicity, treatment will be prematurely discontinued without changing the time schedule of the tumor assessments. A brief overview of the study is given in Figure 1.

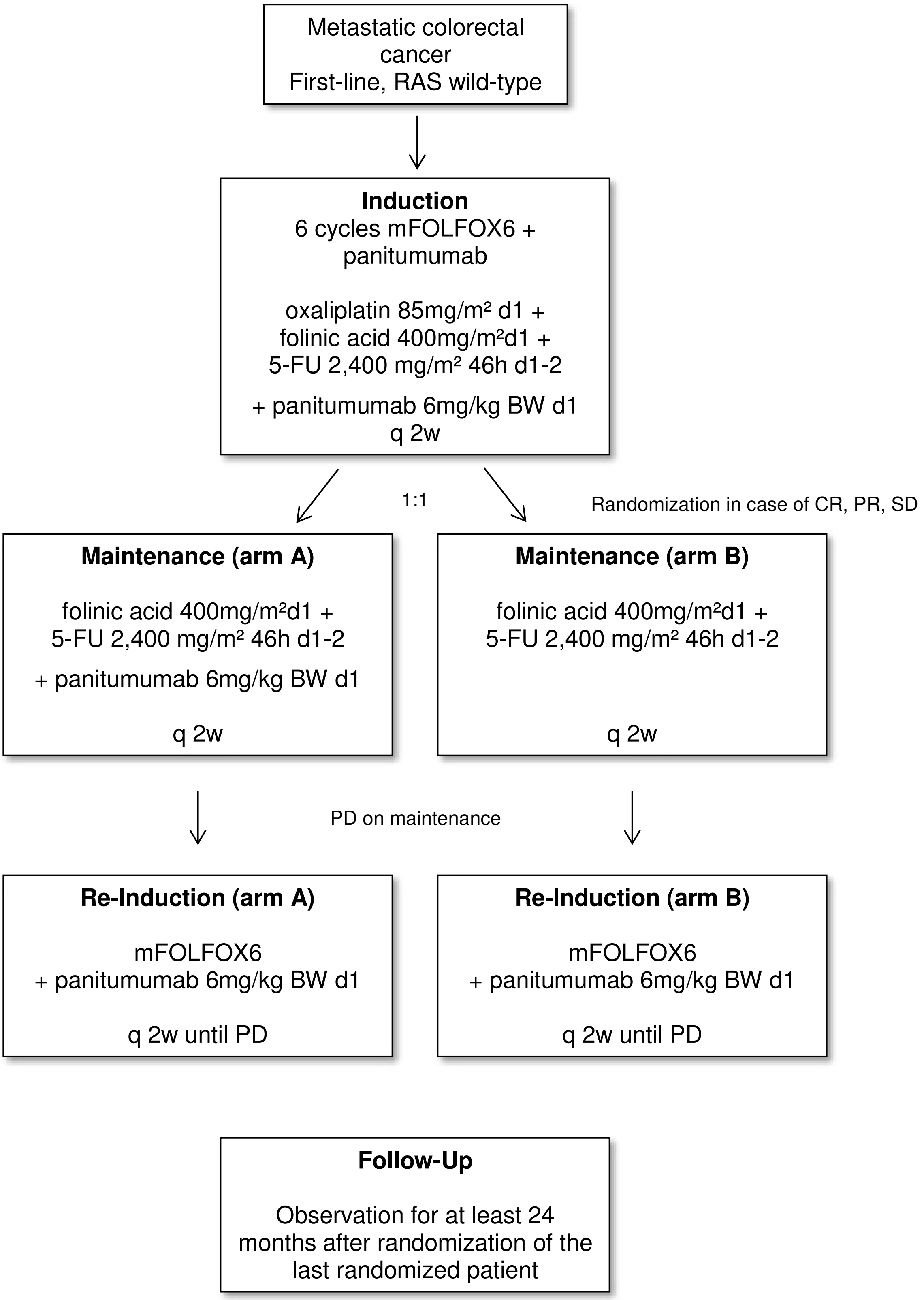

Figure 1: Overview of study design

3.1.1 Definition of Treatment Cycle and Duration

A treatment cycle in this study consists of chemotherapy administration +/- panitumumab on day 1. Cycles will be repeated on day 15. Chemotherapy and study drug administration may be delayed for toxicity according to protocol section 6.3.

The induction with the mFOLFOX6 chemotherapy/ antibody combination will be administered either until diagnosis of disease progression, or up to a maximum of 6 cycles, corresponding to approximately 3 months of treatment.

In case of CR, PR or SD status at the end of the 6-cycle period, randomization will be performed in patients who qualify for maintenance treatment and re-induction treatment with all drug components. Maintenance with 5-FU/ folinic acid ± panitumumab (6 mg/kg body weight [BW], every 2 weeks) is started and repeated until disease progression. At disease progression, mFOLFOX6 plus panitumumab treatment should be re-induced (in order to determine time to failure of strategy).

3.1.2 Treatment Phase

The treatment phase consists of induction therapy, maintenance therapy and re-induction. Subjects who cannot be randomized after induction therapy will have an End of Treatment visit and will be followed-up for survival only. Randomized patients will enter the maintenance phase until they experience disease progression demonstrated by computed tomography (CT) or magnetic resonance imaging (MRI) according to RECIST Version 1.1. Upon progression, patients will undergo re-induction with mFOLFOX6. Re-induction treatment will be continued until progression of disease is demonstrated by CT or MRI. Study treatment will end in case of disease progression during re-induction, or unacceptable toxicities occurring in individual subjects, or consent is withdrawn.

3.1.3 End of Treatment Visit

Patients that discontinue from treatment, regardless of the reason, will perform an End of Treatment assessment 4 weeks (+ 7 days) after Day 1 of the last chemotherapy cycle.

3.1.4 Follow-Up Phase

All patients should be followed. Follow-up evaluations will be performed every 3 months in order to collect information on survival status. Those patients who discontinue study treatment prematurely, i.e., prior to documented disease progression during maintenance or re-induction phase, will remain in the study and will undergo tumor assessments every 8 weeks until progression of disease is demonstrated by CT- or MRI-scan. Thereafter, patients will be followed for survival status every 3 months.

Details of the study schedule are illustrated in the study flow chart (page 11).

3.2 Assignment of Patients to Treatment

According to section 8, a total of approx. 400 patients will be enrolled to reach the planned number of 272 randomizations.

Patients who experience CR/PR or SD 12 weeks after induction therapy and qualify for subsequent maintenance treatment and re-induction treatment with all drug components will be allocated by means of a computer-generated randomization list in a 1:1 ratio to maintenance chemotherapy plus/ minus panitumumab.

The randomization list will be prepared by a statistician using validated SAS software.

Randomization will be performed using a stratified permuted block technique. The block length will be unknown to the centers. To increase homogeneity between the two treatment arms, randomization will be stratified by

- Response to induction therapy at time of randomization (CR/PR vs. SD)
- Prior oxaliplatin-containing adjuvant therapy (yes vs. no)
- Planned starting dose of panitumumab for maintenance therapy, if patient will be assigned to arm A (full dosage vs. reduced dosage)

Registration and randomization are organized centrally by ClinAssess, Germany. If, after check of eligibility a patient who fulfills the inclusion/exclusion parameters is to be included, the center will complete a registration form and fax this page to

ClinAssess GmbH

Fax +49 (0)2171 / 36 336 55

Patients who experience CR/PR or SD 12 weeks after induction therapy and qualify for subsequent maintenance treatment and re-induction treatment with all drug components will be randomized. A randomization form will be sent via fax to ClinAssess, Germany, confirming presence of CR/ PR or SD and qualification for subsequent treatment, as well as providing details for stratification.

Registration forms are accepted at working days between 08:00 am and 06:00 pm. At ClinAssess, forms will be checked, a patient number will be assigned and the confirmation of study participation and treatment assignment of the patient will be faxed back to the center.

The first planned dose of induction chemotherapy and panitumumab will be administered no more than 7 days following registration.

Randomizations will be conducted via eCRF.

3.3 Centers

An approximate number of 95 centers in Germany will participate in the study. Each center is expected to recruit approximately 4-6 patients starting the induction phase until the planned total number of approx. 400 patients or 272 randomizations respectively is reached.

3.4 Study Duration

The study is planned to start in Q4 2013 with respect to first patient in (FPI) including a recruitment period of approx. 84 months, hence the last patient in will be included not prior to Q4 2020. The length of treatment will be approximately 1 year, leading to the last patient off study-treatment approx. in Q4 2021. The total number of events (progressions or deaths, whatever first) required for final analysis of PFS will be reached approx. in Q2 2022. In order to obtain mature data, all randomized patients' minimum observation period of 2 years after randomization of the last subject should be available for each patient randomized to the trial. Therefore, last patient out will be expected approx. in Q4 2022.

**Submission to EC/CA:**	Q3 2013
**First Patient in (FPI):**	Q4 2013
**Recruitment Phase:**	Approx. 84 months
**Last patient in (LPI):**	Approx. Q4 2020
**Final Analysis of primary study endpoint**:	Approx. Q2 2022
**Last Patient out (LPO):**	Approx. Q4 2022

4 Selection of the Study Population

4.1 Target Population

Patients with mCRC in first-line therapy will be eligible for the study. Eligibility criteria are to be fulfilled at the time of registration for induction treatment. Baseline tumor assessment by CT thorax/ abdomen must have taken place within 3 weeks prior to treatment start (day 1 of cycle 1). Under no circumstances are patients, who were once enrolled in this study, permitted to be re-enrolled into the same study. Both male and female patients are eligible for enrollment.

4.2 Inclusion Criteria

- Signed written informed consent
- Male or female ≥ 18 years of age
- Histologically proven metastatic colorectal cancer
- Geno- or phenotypically excluded dihydropyrimidine dehydrogenase (DPD) deficiency (by genotyping of the DPD-coding gene or by uracil plasmalevel measurement)
- Molecular testing showing RAS wild-type in colorectal carcinoma cells
- Life expectancy > 12 weeks
- At least one measurable lesion according to RECIST 1.1
- Adequate bone marrow, liver, kidney, organ and metabolic function
  - Bone marrow function
    - leukocyte count ≥ 3.0 × 10^9/L
    - ANC ≥ 1.5 × 109/L
    - platelet count ≥ 100 × 10^9/L
    - hemoglobin ≥ 9 g/dL or 5.59 mmol/L (may be transfused or treated with erythropoietin to maintain/ exceed this level)
  - Hepatic function
    - Total bilirubin ≤ 1.5 × UNL
    - ALT and AST ≤ 2.5 × UNL (or ≤ 5 × UNL in presence of liver metastases)
    - AP ≤ 5 × UNL
  - Renal function
    - Creatinine clearance ≥ 50 mL/min according to Cockroft-Gault formula or serum creatinine ≤ 1.5 × UNL
  - Metabolic function
    - Magnesium ≥ lower limit of normal
    - Calcium ≥ lower limit of normal
- ECOG performance status 0 – 1
- Women of child-bearing potential must have a negative pregnancy test

4.3 Exclusion Criteria

- Previous treatment for colorectal cancer in the metastatic setting with the exception that patients with urgent need of immediate treatment (high tumor load, symptoms) may have received one cycle of any FOLFOX regimen (no capecitabine!) in case of yet unconfirmed RAS status
- Previous EGFR-targeting therapy < 6 months after end of adjuvant therapy (previous chemoradiation for rectal cancer is accepted for inclusion into the trial and does not account as adjuvant therapy)
- Known brain metastases unless adequately treated (surgery or radiotherapy) with no evidence of progression and neurologically stable off anticonvulsants and steroids
- Chronic inflammatory bowel disease
- Peripheral neuropathy ≥ NCI-CTCAE V 4.03 grade 2
- Other previous malignancies with the exception of a history of previous curatively treated basal cell carcinoma of the skin or pre-invasive carcinoma of the cervix or other curatively treated malignant disease without recurrence after at least 5 years of follow-up
- Significant disease that, in the investigator's opinion, would exclude the patient from the study
- History of cardiac disease; defined as:
  - Congestive heart failure > New York Heart Association (NYHA) class 2
  - Active coronary artery disease (myocardial infarction more than 6 months prior to start of study treatment is allowed)
  - Cardiac arrhythmias requiring anti-arrhythmic therapy (beta-blockers or digoxin are permitted)
  - Uncontrolled hypertension (defined as blood pressure ≥ 160 mmHg systolic and/or ≥ 90 mmHg diastolic on medication)
- Patients with interstitial lung disease, e.g., pneumonitis or pulmonary fibrosis or evidence of interstitial lung disease on baseline chest CT scan
- Known HIV, hepatitis B or C infection
- Known hypersensitivity reaction to any of the study components
- Radiotherapy, major surgery or any investigational drug 21 days before registration
- Pregnancy or lactation or planning to be pregnant during treatment and within 6 months after the end of treatment
- Subject (male or female) is not willing to use highly effective methods of contraception (per institutional standard) during treatment and for at least an additional 6 months after the end of treatment
- Known alcohol or drug abuse

- Any condition that is unstable or could jeopardize the safety of the patient and his compliance in the study

5 Schedule of Assessment and Procedures

Please refer to the study flow chart (page 11) for an overview.

Data will be collected via the completion of an electronic Case Report Form (eCRF) for each eligible patient. The investigator should confirm eligibility of the patient according to the inclusion and exclusion criteria of the study. All patients have to provide written Informed Consent before any study specific assessment is performed. A study specific assessment is defined as a procedure that is not part of the routine assessments performed for diagnostic purposes or standard care. Screening assessments should occur within 21 days of the first administration of induction therapy.

Patients not meeting the eligibility criteria will not be enrolled into the study. Patients should receive their first dose of study treatment as soon as possible after registration, but not later than 7 days after registration.

5.1 Study Assessments

5.1.1 Tumor Assessments

Objective response will be evaluated based on RECIST criteria Version 1.1 (see Appendix 4) using CT scan or MRI scan. For patients with multiple measurable lesions, up to 5 lesions in total and 2 lesions per organ should be identified.

- If brain metastases are suspected or known, a CT or MRI scan of the brain will be performed. Patients with brain metastases are excluded.
- If suspicion of bone metastasis, a radiologic/ scintigraphic assessment should be performed

The exact technique used for measurement of lesions (i.e., either CT or MRI scan) will be left to the discretion of the investigator, however, for each patient the same technique must be used throughout the study, assessed whenever possible by the same individual. All lesions identified at screening have to be assessed at each scheduled tumor measurement. Patients with measurable lesions will be eligible for inclusion. Measurable lesions must have at least one diameter of 10 mm by CT scan (CT slice thickness no greater than 5 mm. When CT scans have slice thickness greater than 5 mm, the minimum size for a measurable lesion should be twice the slice thickness). Where there are several lesions, assessment is based on the sum of the longest diameters of the individual target lesions. Both – longest and shortest diameters should be recorded in the clinical report. Lymph nodes with a short axis of ≥ 15 mm are considered measurable and assessable as target lesions. The short axis measurement should

be included in the sum of lesions in calculation of tumor response. Nodes that shrink to < 10 mm short axis are considered normal. All other pathological nodes (those with short axis ≥ 10 mm but < 15 mm) should be considered non-target lesions.

In cases where there is suspicion of progression before the next scheduled assessment, an unscheduled tumor assessment should be performed.

In case a detected increase in tumor size is below the resolution limit of the CT/ MRI scanner, it is accepted to continue with treatment until a second assessment at a later time point unequivocally confirms progressive disease.

The following are defined as non-target lesions: bone lesions, leptomeningeal disease, pleural/pericardial effusion, ascites, inflammatory breast disease, lymphangitis, cystic lesions and lesions not measurable by computed tomography (CT) or magnetic resonance imaging (MRI). All non-target lesions are described over time and need not be measured.

The tumor assessment for inclusion should be recorded and measured within 21 days prior to treatment start of cycle 1. If a cycle of any FOLFOX regimen has been administered before enrolment due to urgent treatment need and yet unconfirmed RAS status this baseline CT / MRI must be performed before start of that cycle and still within 21 days prior to start of cycle 1. Please take care to avoid any delays within that tight schedule.

Tumor assessments will be performed 12 weeks after treatment start with induction therapy and every 8 weeks during maintenance therapy and re-induction. During the maintenance and re-induction period, disease assessments will be performed every 8 weeks, independent of chemotherapy cycle delay.

Please note that for subjects discontinuing maintenance or re-induction therapy prior to documentation of disease progression, tumor assessments will be performed at 8 week intervals (+/- 7 days) until disease progression is documented.

In order to assess progression during maintenance therapy, tumor assessments during maintenance will be compared to the tumor assessment performed at randomization (i.e., tumor assessment in week 12 of the study). In order to assess progression during re-induction, the tumor assessment at the start of re-induction therapy will serve as a reference (i.e., that tumor assessment showing progression during maintenance (1st progression)).

5.1.2 Other Clinical Assessments

- ECOG performance status

To be eligible for study entry, patients must have an ECOG performance score of 0 or 1. The patients' ECOG performance status will be assessed at the screening visit (within 7 days prior to treatment start), before every chemotherapy cycle during the treatment phase, at the EOT

visit that will take place 4 weeks after day 1 of the last chemotherapy cycle, and during follow-up.

- 12-lead ECG

A 12-lead ECG will be measured at screening. ECGs will only be performed during the treatment phase and for the EOT visit if clinically indicated.

- Physical examination

A physical examination will be performed at the screening visit, before start of each treatment cycle, at the EOT visit and at the FU visits (documentation of physical examination during follow up only until progression if treatment was discontinued before progression of disease occurred).

- Vital signs

Vital signs (blood pressure, heart rate, and body temperature) will be measured at the screening visit, before start of each treatment cycle and at the EOT visit. Body weight will be measured at the screening visit, before the start of each treatment cycle, at the EOT visit and for follow-up visits (documentation of body weight during follow up only until progression if treatment was discontinued before progression of disease occurred). Body height will be measured at screening only.

- Quality of life

Quality of life will be evaluated using the validated EORTC QLQ-C30, FACT-EGFRI 18, SKINDEX-16 and DLQI, which need to be filled out by the patient. Quality of life questionnaires will be completed at the following timepoints:

- Within 21 days before start of therapy (screening)
- Day 1 of 1st Induction cycle
- Day 1 of 2nd Induction cycle
- **Day 1 of every 2nd** following cycle, that is:
    - Induction cycle 4,
    - Induction cycle 6
    - Maintenance cycle 2 (Overall cycle 8)
    - Maintenance cycle 4 (Overall cycle 10)
    - etc., with ongoing two-cycles interval also when entering re-induction phase

Examples:

- Patient with 6 cycles maintenance is entering re-induction: QoL in re-induction Cycles 2, 4, 6, etc.
- Patient with 7 cycles maintenance is entering re-induction: QoL in re-induction cycles 1, 3, 5, etc.

- EOT visit

5.1.3 Safety Assessments

Throughout the treatment period until the EOT visit, patients will be assessed for all adverse events. Common terminology criteria for adverse events (CTCAE v4.03) will be used for grading. If necessary, the patient may be withdrawn from the study treatment.

- **Medical history** including concurrent illnesses and information on dates and description of initial diagnosis of colorectal cancer and prior cancer treatment history will be reviewed and recorded at the screening visit. *RAS* mutational status (exon 2 [codons 12/13], exon 3 [codons 59/61], and exon 4 [codons 117/146] of *KRAS* **and** *NRAS*) needs to be determined before inclusion of the patients in the study.
- **Concomitant medications** will be documented during screening and throughout the treatment phase until the EOT visit.
- **Adverse events** (see also section 7): All patients will be closely monitored for adverse events from the date of informed consent through the end of treatment visit. Adverse events should be followed up until they have returned to baseline status or stabilized.
- **Pregnancy test** a serum β-HCG test within 7 days before the first dose of study drug will be performed for women with childbearing potential. A urine test will be done if the date of the first result exceeds the 7 day window.

5.1.4 Laboratory Assessments

Blood samples will be taken for hematological, serum chemistry monitoring and tumor marker measurement (CEA and CA 19-9) at screening, during the treatment phase before start of each treatment cycle and at the end of treatment visit. The local laboratory will perform the analyses and provide reference ranges.

5.1.5 Translational analysis

Blood samples (Up to 4 sampling timepoints: on day 1 of cycle 1, on day 1 of maintenance-therapy at the end of maintenance therapy (i.e. day of PD diagnosis) and at the end of treatment visit) and paraffin-embedded tissues will be collected and characterized.

The tissue block may be collected at initial diagnosis or during earlier treatment (operations, biopsies) of the patient. Parameters analyzed include DNA, RNA, immunohistochemistry, FISH, immunological staining and analyses using blood samples (cells and soluble targets like tumor markers, cell-free DNA etc.).

Tissue blocks and blood samples will be evaluated by:

Prof. Dr.med. Dominik Paul Modest

Charité Universitätsmedizin Berlin

Campus Virchow Klinikum (CVK)

Medizinische Klinik m.S. Hämatologie, Onkologie und Tumorimmunologie

Geländeadresse: Ostring 4, Dachgeschoss, Studienzentrale

Augustenburger Platz 1

13353 Berlin

SHIPMENT address (until further notice):

Prof. Dr.med. Dominik Paul Modest

c/o AG Onkologie Prof. Dr. Volker Heinemann

Med. Klinik III

Klinikum der Universität (LMU)

Marchioninistrasse 15

81377 München

5.1.6 Digital imaging

All patients will be asked to give their consent that CT/MRI images series acquired throughout the study can be copied and archived to be re-read centrally for research purposes. Patient images will be provided by study sites without any confidential patient data and stored confidentially in an image database maintained by

Prof. Dr.med. Dominik Paul Modest
Charité Universitätsmedizin Berlin
Campus Virchow Klinikum (CVK)
Medizinische Klinik m.S. Hämatologie, Onkologie und Tumorimmunologie
Geländeadresse: Ostring 4, Dachgeschoss, Studienzentrale
Augustenburger Platz 1
13353 Berlin

SHIPMENT address (until further notice):

Prof. Dr.med. Dominik Paul Modest
c/o AG Onkologie Prof. Dr. Volker Heinemann
Med. Klinik III
Klinikum der Universität (LMU)
Marchioninistrasse 15
81377 München

5.2 Study Procedures

5.2.1 Screening Procedures

Data, that have been generated / obtained as part of routine care before the subjects informed consent was available may be documented to determine eligibility only after the subjects informed consent has been obtained. All patients will be screened and screening procedures performed within 21 days prior to the start of induction treatment (Cycle 1 of induction treatment). These include the following:

Signed Informed Consent	Obtained prior to any study specific assessment
Demographics and medical history	• Age, gender, race<br>• Tumor diagnosis including dates and description of initial diagnosis of colorectal cancer<br>• Previous and concurrent relevant diseases other than colorectal cancer<br>• Current symptoms and/ or residual toxicities from prior therapies<br>• Previous treatments for colorectal cancer<br>• BSA
RAS Status	• Determination (or acquisition if existing) of *RAS* status (exon 2 [codons 12/13], exon 3 [codons 59/61], and exon 4 [codons 117/146] of *KRAS* **and** *NRAS*)
Tumor Assessments<br>• Target lesions	See section 5.1.1
• CT or MRI of the brain	In case of neurological symptoms, suspected or known brain metastases
• Bone scans	Bone scans will be performed only if clinically indicated at screening. Bone lesions noted on a screening scan should be confirmed and followed by plain X-ray (bone lesions are not target lesions).
Cardiac evaluation	12-lead ECG
Physical examination and vital signs<br>(Day -7 to 0)	• Physical examination<br>• Height (at screening only), weight<br>• Vital signs measurements will include body temperature, heart rate and blood pressure
ECOG performance status<br>(Day -7 to 0)	See Appendix 3
Serum pregnancy test<br>(Day -7 to 0)	A serum pregnancy test will be performed in pre-menopausal women and women who are menopausal for < 2 years. In case the sampling date for serum pregnancy testing exceeds 7 days before treatment start, a urine test is required for confirmation of the absence of pregnancy.
Hematology (Day -7 to 0)	Hemoglobin, erythrocytes, platelets, leukocytes, ANC. In case a cycle of any FOLFOX regimen has been applied prior to enrollment due to urgent treatment need, the hematologic baseline values can be obtained from evaluation prior to or at that cycle to prevent bias of the assessment due to chemotherapy

Clinical chemistry<br>(Day -7 to 0)	Sodium, potassium, calcium, magnesium, alkaline phosphatase, urea, AST, ALT, LDH, total bilirubin, albumin, creatinine, total protein, creatinine clearance (by Cockroft-Gault formula)
Tumor Markers	Carcinoembryonic antigen (CEA) and CA 19-9
Concomitant medication	Concomitant medication currently used
Quality of Life	EORTC QLQ-C30, FACT-EGFRI 18, Skindex-16, DLQI
Paraffin-embedded archived tumor samples	Optional, investigational analyses, see section 5.1.5
Translational analyses in serum and whole blood samples	Optional, investigational analyses, see section 5.1.5

5.2.2 Treatment Phase

During the treatment phase the following assessments are to be performed:

Concomitant medications<br>Adverse events<br>Skin toxicity	Assessed on an ongoing basis
Physical examinations and vital signs	Day 1 of each chemotherapy cycle<br>• Physical examination will include weight<br>• BSA<br>• Vital signs measurements will include body temperature, heart rate and blood pressure.
ECOG performance status	Day 1 of each chemotherapy cycle
ECG	If clinically indicated
Hematology	Within 2 days prior to each chemotherapy cycle: hemoglobin, erythrocytes, platelets, leukocytes, ANC
Clinical chemistry	Within 2 days prior to each chemotherapy cycle:<br>Sodium, potassium, calcium, magnesium, alkaline phosphatase, urea, AST, ALT, LDH, total bilirubin, albumin, creatinine, total protein, creatinine clearance (by Cockroft-Gault formula)
Tumor Markers	Carcinoembryonic antigen (CEA) and CA 19-9 after 6 cycles of induction therapy before randomization
Tumor measurement (CT or MRI)	12 weeks after start of the induction therapy and every 8 weeks during maintenance and re-induction according to RECIST 1.1
Quality of Life	The EORTC QLQ-C30, FACT-EGFRI 18, Skindex-16, DLQI questionnaires will be completed on Day 1 of each chemotherapy cycle
Translational analyses in serum and whole blood samples	• Optional at day 1 of cycle 1 and day 1 of maintenance therapy, investigational analyses, see section 5.1.5<br>• Additionally, another optional blood sample set should be collected at the end of maintenance phase on day of PD diagnosis.

If secondary resectability of metastasis is achieved, hepatic surgery is performed according to the standards of the respective institution, but should follow the published S1 guidelines of the German Cancer Society. A time interval of about 21 days between last chemotherapy application and surgery is recommended. These patients will have an End of Treatment visit

performed 4 weeks after last chemotherapy and will be followed-up every 12 weeks for at least two years.

5.2.3 End of Treatment

Patients who discontinued therapy for any reason must have an End-of-Treatment-Visit completed 4 weeks (+ 7 days) after day 1 of the last chemotherapy cycle. This assessment should include ECOG performance status, physical examination, weight, vital signs, quality of life assessment, hematology, clinical chemistry, concomitant treatments and adverse events. An ECG will be performed if clinically indicated. An optional whole blood and serum sample will be collected if the patient agrees to translational research project.

5.2.4 Follow-Up

After discontinuation of treatment, all patients will be followed-up every 3 months. The first visit of the long-term follow-up period will take place 3 months after the EOT visit. Overall collection of long-term follow-up data will terminate 2 years after randomization of the last subject. The following information will be collected during the follow-up visits:

- Physical examination
- Body weight
- ECOG performance status
- Survival data
- Subsequent anti-cancer therapy
- Adverse events continuing from treatment phase will be followed-up until the event has resolved or stabilized.

For subjects discontinuing maintenance or re-induction therapy prior to documentation of disease progression, tumor assessments will be performed at 8 week intervals (+/- 7 days) until disease progression is documented.

5.2.5 End of Study

The study will terminate approx. 2 years after randomization of the last patient.

5.3 Planned Treatment of the Patient after End of Treatment Phase

After completion of the study at routine follow-up (EOT), patients will generally be treated at the discretion of the investigator according to medical routine.

5.4 Removal of Patients from Treatment

Subjects will be free to discontinue treatment or withdraw from the study at any time, for any reason, or they may be withdrawn/ removed if necessary in order to protect their health (see reasons for withdrawal below). It is understood by all concerned that an excessive rate of

withdrawals can render the study uninterpretable; therefore, unnecessary withdrawal of subjects should be avoided. Patients who are withdrawn from the study will not be replaced.

Patients will be removed from further study treatment for the following reasons:

- Disease progression during re-induction therapy
- Continued unacceptable toxicities despite optimal treatment or dose reduction

- Administration of any other anti-neoplastic medication or any other experimental drug
- Investigator decision in the best interest of the patient
- Withdrawal of consent
- Non-compliance/ lost to follow-up
- Pregnancy
- Termination of the study by the sponsor
- Death

Treatment delays for more than 6 weeks from the previous dose of Panitumumab or Chemotherapy should only occur if medically founded. Treatment delays for other than medical reasons should be avoided. Study treatment continuation after such long delays without medical justification should be evaluated carefully by the investigator, also with regard to data interpretation.

If there is a medical reason for withdrawal of treatment, the patient will remain under the supervision of the investigator until the AEs have been resolved or declined to baseline values. If a patient has failed to attend scheduled assessments in the study, the investigator must determine the reasons and circumstances as completely and accurately as possible.

In case of premature discontinuation of the study treatment, the investigations scheduled for the EOT and the follow-up visits should be performed, if possible. The CRF section entitled "End of Treatment" must be completed in all cases. Should a patient decide to withdraw, every effort will be made to complete and report the observations as thoroughly as possible. The investigator should contact the patient to determine as completely as possible the reason for the withdrawal. A complete final evaluation at the time of the patient's withdrawal should be made, with an explanation of why the patient is withdrawing from the study. If the reason for removal of a patient from the study is an adverse event or an abnormal laboratory test result, the principal specific event or test will be recorded on the case report form.

If a patient withdraws consent for further study treatment, the patient should still be followed for progression and survival. If a patient withdraws consent for further participation in the study, follow-up assessments will be discontinued.

5.5 Study Discontinuation

The whole study may be discontinued at the discretion of the sponsor in the event of any of the following:

- Medical or ethical reasons affecting the continued performance of the study
- Difficulties in the recruitment of patients
- Occurrence of AEs unknown to date in respect of their nature, severity, and duration or the unexpected incidence of known AE

6 Investigational Product

6.1 Investigational medicinal product (IMP)

An investigational medicinal product is a pharmaceutical form of an active substance or placebo being tested or used as a reference in a clinical trial, including products already with a marketing authorization but used or assembled (formulated or packaged) in a way different from the authorized form, or when used for an unauthorized indication, or when used to gain further information about the authorized form.

The investigational study drug of this trial is panitumumab only. As 5-fluorouracil/ leucovorin and oxaliplatin are generally available and form the routine treatment of advanced colorectal cancer, they are considered as chemotherapy backbone medication. Thus, they will be prescribed by the treating physician, as this prescription is within the framework of standard, approved usage. The same holds for the components of the prophylactic skin treatment, as they are routinely prescribed in case of skin reactions of the types to be expected in this study.

The supply of panitumumab is supported by AMGEN GmbH as a research grant. It will be delivered to the participating centers free of charge. Details on the distribution will be described in a specific operation procedure with appropriate forms attached.

The investigator or a pharmacist or other appropriate individual, who is designated by the local principal investigator, should maintain records of the inventory at the site, the use for each subject, and the delivery, storage and destruction. Investigators should maintain records that adequately document that subjects were provided the doses specified in the protocol and reconcile all investigational product(s) received from the sponsor.

The investigator should ensure that the investigation product(s) are used only in accordance with the protocol.

6.2 Preparation and Administration of test drug panitumumab and chemotherapy

6.2.1 Preparation and Administration of panitumumab

6.2.1.1 Drug Name, Formulation and Storage

Drug name:

INN: Panitumumab

Trade name: Vectibix®

Manufacturer: Amgen Ltd.

Formulation:
Each vial of panitumumab will contain 5, 10 or 20 mL of a sterile, colorless, preservative-free protein solution containing a 20 mg/mL solution of panitumumab. The vial will contain approximately 100 mg, 200 mg or 400 mg of panitumumab and is for single dose use only.

Labeling:
Each vial of panitumumab will be labeled in accordance with current ICH GCP, FDA and specific national requirements.

Storage:
Panitumumab must be stored at 2-8 °C (36° to 46°F) in a secured area upon receipt. Vials are to be stored in the original carton under refrigeration at 2-8 °C (36° to 46°F) until time of use. The product should be protected from direct sunlight and should not be frozen or shaken excessively. Exposure of the material to excessive temperature above or below this range should be avoided. Do not allow panitumumab to freeze and do not use if contents freeze in transit or in storage. If vials fall out of specified temperature requirement, please contact Amgen for instructions.

As panitumumab contains no preservative, vials are designed for single use only. Any unused portion of panitumumab remaining in the vial must not be used. The diluted solution should be used ≤ 6 hours after dilution, if stored at room temperature, or ≤ 24 hours after dilution if stored refrigerated at 2° to 8°C (36° to 46°F).

Records of the actual storage condition during the period of the study should be maintained.

6.2.1.2 Route of Administration

Panitumumab will be administered as an intravenous infusion.

6.2.1.3 Preparation of Study Drug

NOTE: Panitumumab is a protein and should be handled gently to avoid foaming, which may lead to denaturation of the protein product. This precaution applies not only to panitumumab stored in the vial, but also for diluted panitumumab prepared in the IV bag. It is, therefore, essential to avoid medication delivery methods, particularly pneumatic tube systems that could potentially lead to excessive shaking or vibration that would lead to particulate formation in the protein product.

The pharmacist, using aseptic techniques, will prepare the panitumumab infusion. The dose of panitumumab will be 6 mg/kg and will be based upon the subject’s baseline weight. The dose will not be recalculated unless the weight changes at least ± 10% from the baseline weight. It is recommended that the calculated amount of panitumumab (may be rounded to the nearest tenth milligram [e.g., 456 mg rounded to 460 mg or 312 mg rounded to 310 mg]) is

removed from the vials and added to a total volume of 100 mL of pyrogen-free 0.9% sodium chloride solution. **The maximum concentration of the diluted solution to be infused should not exceed 10 mg/mL.** Doses higher than 1000 mg should be diluted to 150 ml with 0.9% sodium chloride injection. The diluted solution should be mixed by gentle inversion, do not shake. Once diluted, panitumumab should be used ≤ 6 hours after dilution if stored at room temperature, or ≤ 24 hours after dilution if stored refrigerated at 2° to 8°C (36° to 46°F).

Panitumumab does not contain antimicrobial preservatives or bacteriostatic ingredients.

The bag should be labeled per site pharmacy standard operating procedures and promptly forwarded to the clinic center for infusion.

No incompatibilities have been observed between panitumumab and sodium chloride injection in polyvinyl chloride bags, polyolefin bags, or glass.

Panitumumab 6 mg/kg will be administered IV by an infusion pump through a peripheral line or indwelling catheter using a non-pyrogenic, low protein binding filter with a 0.2- or 0.22-micron pore size in-line filter infusion set-up over 60 minutes by a trained healthcare professional. If a dose of panitumumab is well tolerated (i.e., without any serious infusion-related reactions), then subsequent IV infusions of panitumumab may be administered over 30 - 60 minutes. In the event a subject's actual body weight requires an infusion with a volume greater than 150-mL, panitumumab will be administered over approximately 90 minutes .

Panitumumab should not be administered as an IV push or bolus.

Panitumumab should not be mixed with, or administered as, an infusion simultaneously with other medicinal products. The infusion line should be thoroughly flushed with saline (supplied by the center) before and after administration of panitumumab to avoid mixing with other drug products or IV solutions.

6.2.1.4 Compliance

The investigator or a pharmacist or other appropriate individual, who is designated by the local principal investigator, should maintain records of the inventory at the site, the use for each subject and delivery, storage and destruction. Investigators should maintain records that adequately document that subjects were provided the doses specified in the protocol and reconcile all investigational product(s) received from the sponsor.

The investigator should ensure that the investigational product(s) are used only in accordance with the protocol. All doses given are to be documented in the CRF, including exact dose and date administered.

The investigational product(s) should be stored as specified by the sponsor and in accordance with applicable regulatory requirements.

6.2.2 Preparation and Administration of chemotherapy

All drugs used in the study are labeled for treatment of advanced colorectal cancer. Clinical use and administration should follow the respective product/prescribing information. Administration of mFOLFOX6 or 5-FU/ FA chemotherapy will commence on day 1 of each treatment cycle. Chemotherapy will be administered after administration of panitumumab.

6.2.2.1 Oxaliplatin

Mode of action: DNA cross-linkage

Administration: intravenous. For specific formulation, packaging, preparation and administration information, please refer to the instructions provided in the oxaliplatin product labeling. **Oxaliplatin has to be administered before the application of 5-FU. Do not dilute oxaliplatin with NaCl-solution or other chloride containing solutions.**

Aluminium containing equipment should not be used for administration of oxaliplatin as there is a risk of oxaliplatin degradation.

Common side effects: nausea, vomiting, diarrhea, leukopenia, thrombocytopenia, anemia, increase of liver enzymes, alopecia, peripheral neurotoxicity.

Please refer to the SmPC for further details.

6.2.2.2 5-Fluorouracil

Mode of action: anti-metabolite

Administration: as continuous intravenous infusion after calculation of the individual 5-FU dose requirement (rounded up to the nearest 10mg)

Common side effects: Mucositis, stomatitis, diarrhea, anorexia, alopecia, hematological side effects (mainly leucopenia, more rarely thrombocytopenia). The white blood cell nadir occurs 9-14 days after treatment. Rare side effects are hepatotoxicity, pulmonary toxicity, neurotoxicity, cardiotoxicity and dermatitis. Interaction: Allopurinol should not be co-administered with 5-FU since it may decrease its effect.

Contra-indication: Known DPD deficiency. (NOTE: test is highly recommended according to current prescribing information)

6.2.2.3 Folinic Acid

Mode of action: Folinic acid (FA) stabilizes the complex between FdUMP and thymidilate synthase and thereby increases the inhibitory effect on DNA synthesis.

Administration: as intravenous infusion after calculation of the individual FA dose requirement (rounded up to the nearest 10 mg).

Side effects: Side effects are gastrointestinal upsets; also allergic reactions are possible. Interactions are conceivable with all drugs that interfere with folinic acid metabolism (e.g., allopurinol, trimethoprim, pyrimethamine). Folinic acid can reduce the therapeutic effects of antiepileptic agents such as phenobarbital, primidon, phenytoin or succinimid and may thus cause an increase in epileptic seizures.

Please refer to the Summary of Product Characteristics for details on description, preparation, administration and precautions for use.

6.3 Dose Modifications

Toxicity will be graded according to NCI-CTCAE, version 4.03; the therapy modifications described below are applied according to this severity grading.

Toxicities of severity grade 1 only will not lead to any dose reduction or cycle delay. The same holds for adverse reactions without any potential of serious or life-threatening complications according to the judgment of the physician (e.g., alopecia). Presumably, severe overlapping toxicity between chemotherapy and antibody will not occur (except for diarrhea). Thus, in case of toxicity requiring treatment modification, this alteration should reflect the causal relationship of the respective drug(s). E.g., if the toxicity is unequivocally caused by only one drug, a dosage modification of the other drugs is not required. Likewise, a delay of chemotherapy or the antibody does not lead to the delay of the other modality.

If more than one different type of toxicity occurs concurrently, the most severe grade will determine the modification.

In case of a necessary dose reduction the lower dose level will be applied throughout the rest of the therapy without re-escalation (except for panitumumab, see section 6.3.1). If toxicity requires a treatment delay of more than 3 weeks after the last regularly scheduled dosing date (i.e., 5 weeks after last regular treatment with **all** treatment components), study treatment continuation should be evaluated carefully by the investigator.

In case of acute allergic reactions of grade 3 or 4, the respective agent should be discontinued permanently; in case of grade 1 or 2, it is up to the physician to continue treatment without dose modification and using pre-treatment IV antihistamines and corticosteroids, if this is in the best interest of the patient.

Each dose modification or treatment delay has to be documented in the CRF, including the respective reason.

6.3.1 Dose Modifications for panitumumab

6.3.1.1 Interruption of panitumumab infusion

Subjects who experience any serious infusion reaction during panitumumab administration will have the infusion stopped. Continuation of dosing will be based on the severity and resolution of the event and will be at the discretion of the investigator.

Suspected infusion reactions should be reported as an adverse event. All subjects who experience such an event will be followed for safety.

6.3.1.2 Panitumumab dosage adjustments

For subjects who experience toxicities while on study, one or more doses of panitumumab may need to be withheld, reduced, or delayed (administered at > 14 day intervals). On resolution of toxicity, a limited number of attempts to re-escalate reduced panitumumab doses will be allowed (outlined in Figure 2). Dose escalations above 6 mg/kg starting dose are not allowed. Panitumumab dose reductions are listed in Table 1.

Table 1: Panitumumab dose reductions

	Starting dose	1st dose reduction	2nd dose reduction
Percentage (%)	100	80	60
mg/kg	6	4.8	3.6

Figure 2: Panitumumab dose modification algorithm for toxicity

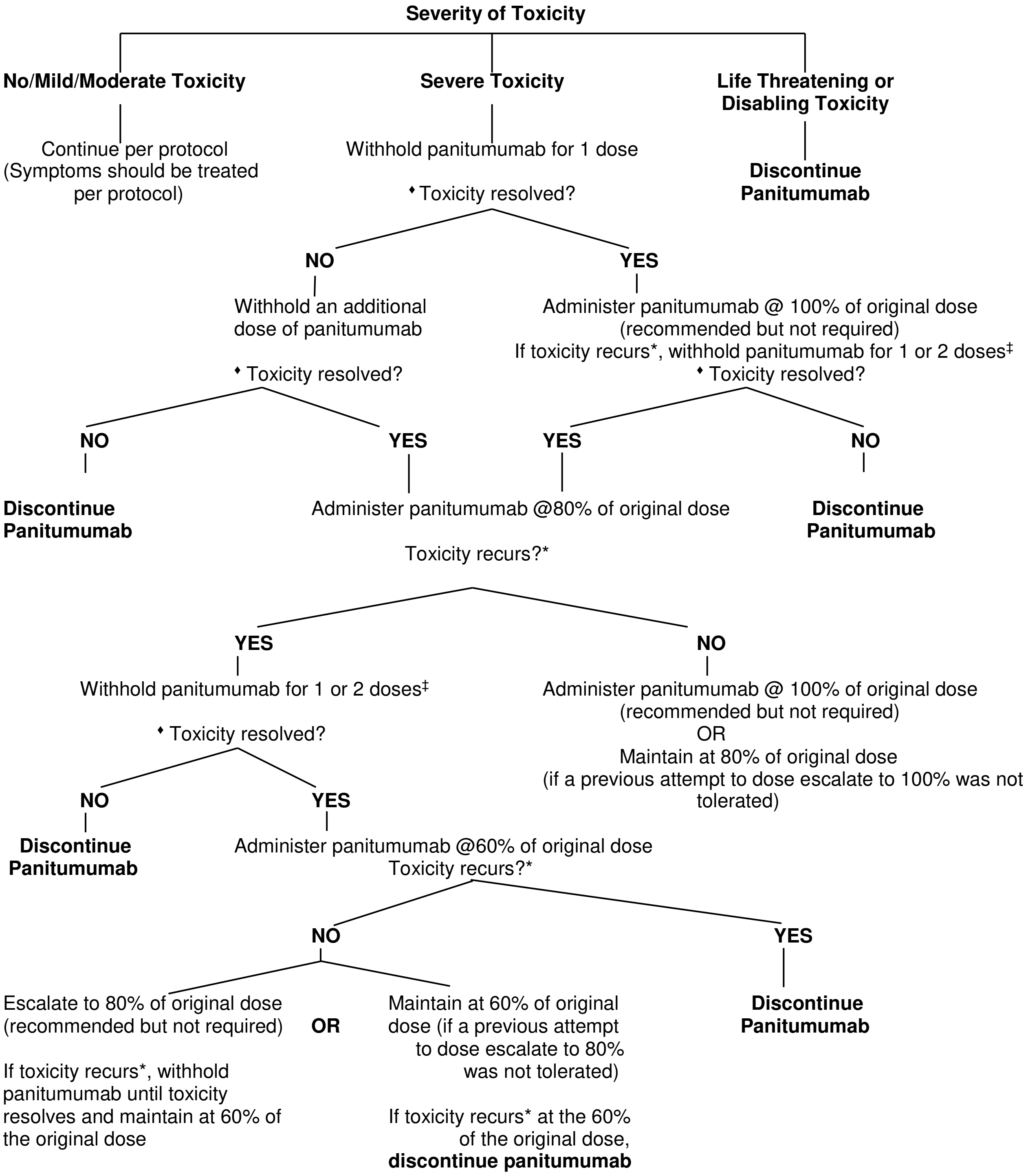

* Assess toxicity before each cycle. Toxicity recurs = meets the criteria for withholding a dose of panitumumab at any time during the study (see Section 6.3.1.3)

♦ Assess toxicity before each cycle. Toxicity resolved = meets the criteria for restarting panitumumab (see section 6.3.1.4). Subjects from whom > 2 subsequent cycles of panitumumab are required to be withheld should not be re-treated with panitumumab.

‡ Up to 2 subsequent doses of panitumumab may be withheld but panitumumab may not be withheld longer than 6 weeks from the previous dose. The second dose should only be withheld if the toxicity has not resolved by the time that the subsequent cycle of chemotherapy is due.

6.3.1.3 Criteria for withholding a dose of panitumumab

Skin- or nail-related toxicities:

- Symptomatic skin- or nail-related toxicity requiring narcotics, systemic steroids, or felt to be intolerable by the subject
- Skin or nail infection requiring IV antibiotic or IV antifungal treatment
- Need for surgical debridement
- Any skin- or nail-related serious adverse event

An algorithm for managing specific panitumumab-related adverse events is shown in Figure 2.

Non-skin- or nail-related toxicities:

- Any grade 3 or 4 toxicity with the following exceptions:
    - Panitumumab will only be withheld for symptomatic hypomagnesemia and/or hypocalcemia that persists despite aggressive magnesium and/or calcium replacement
    - Panitumumab will only be withheld for grade 3 or 4 nausea, diarrhea, or vomiting that persists despite maximum supportive care
    - Panitumumab will only be withheld for grade ≥ 3 anemia or grade 4 thrombocytopenia that cannot be managed by transfusion(s) or cytokine therapy

6.3.1.4 Criteria for re-treatment with panitumumab

Panitumumab may be restarted once:

Skin- or nail-related toxicities:

- The adverse event has improved to ≤ grade 2 or returned to baseline or
- The subject has recovered to the point where symptomatic skin- or nail-related toxicity is felt to be tolerable or
- Systemic steroids are no longer required or
- IV antibiotic or IV antifungal treatment is no longer required

Non-skin or nail-related toxicities: Panitumumab administration may be restarted once the adverse event has improved to ≤ Grade 1 or returned to baseline.

6.3.1.5 Dose modification schedule

Subjects should be assessed for toxicity before each dose. Dose modification should be performed according to the schedule described below and outlined in Figure 2.

Subjects who develop a toxicity that does not meet the criteria for withholding a dose of panitumumab (Section 6.3.1.3) should continue to receive panitumumab and their symptoms should be treated.

Panitumumab-related toxicity will be considered resolved if it improves to a degree that allows for re-treatment with panitumumab (Section 6.3.1.4).

For subjects who experience a toxicity that meets the criteria for withholding a dose of panitumumab:

- Subjects receiving either 100% or 80% of the starting dose of panitumumab are allowed to have up to 2 subsequent doses withheld for toxicity. However, a second dose should only be withheld if the toxicity has not resolved by the time that the subsequent cycle of chemotherapy is due.
- Subjects treated at the 100% dose level whose toxicity resolves after 1 dose of panitumumab is withheld should be re-started at the 100% dose level (recommended but not required, reduction to the 80% dose is allowed as an alternative to re-challenge with the 100% dose).
- If toxicity recurs, subjects treated at the 100% dose or 80% dose should be re-started at the 80% dose or 60% dose, respectively, when the toxicity resolves after withholding 1 or 2 doses of panitumumab.
- Subjects treated at the 100% dose level whose toxicity resolves only after 2 subsequent doses of panitumumab are withheld should be re-started at the 80% dose level.
- Subjects treated at the 80% dose level whose toxicity resolves after withholding 1 or 2 doses of panitumumab should be re-started at the 60% dose level.
- Subjects who experience toxicity at the 60% dose level will not be re-treated with panitumumab.

It is recommended that panitumumab doses will be escalated in subjects whose toxicity resolves to the degree that meets the criteria for re-starting a dose of panitumumab (Section 6.3.1.4). Dose escalations are recommended but not required. Dose escalations should occur in the following manner:

- Subjects treated at the 80% dose level whose toxicity does not recur should receive the 100% dose level at the next cycle unless a previous attempt to re-escalate to the 100% dose level was not tolerated (re-initiation of the 80% dose is allowed as an alternative to dose escalation).

- Subjects treated at the 60% dose level whose toxicity does not recur should receive the 80% dose at the next dose unless a previous attempt to re-escalate to the 80% dose level was not tolerated (re-initiation of the 60% dose is allowed as an alternative to dose escalation).

Subjects who must have a delay of panitumumab administration beyond 6 weeks from the previous dose of panitumumab (i.e., 3 or more consecutively missed doses) due to toxicity, will be considered unable to tolerate panitumumab and will not be re-treated with panitumumab. Nevertheless, those patients will remain in the study and chemotherapy without panitumumab is continued as scheduled.

If a subject demonstrates a clinical benefit with a demonstrated response of stable disease, partial response or complete response and there are reasons that the above dose modification rules cannot be implemented, the investigator should contact and discuss the reasons with the sponsor. The investigator must obtain written agreement from the sponsor before any changes in the dose modification rules can be implemented.

6.3.1.6 Panitumumab delayed or missed doses

Panitumumab should be given on the first day of each chemotherapy cycle. Delays of panitumumab administration beyond 6 weeks from the previous dose of panitumumab are not allowed.

Reasons to withhold a dose of panitumumab are described in Section 6.3.1.3. If a subject is able to receive a cycle of FOLFOX but panitumumab must be withheld due to toxicity, FOLFOX should be administered, and this dose of panitumumab will be considered missed. For all subjects, delays of panitumumab beyond 6 weeks from the previous dose of panitumumab (i.e., 3 or more consecutive missed doses) are not allowed and panitumumab therapy will be permanently discontinued. Missed panitumumab doses will not be made up.

6.3.2 Dose Modifications for chemotherapy

6.3.2.1 General aspects

If severe hematological or non-hematological toxicity should occur, the dose modifications provided in Table 2 are recommended for the subsequent cycle. Please refer to section 6.4 for general pre-conditions for the start of a new cycle. A treatment delay of one component of the chemotherapy regimen (i.e. oxaliplatin, 5-FU) results in a delay of the other component to allow both therapies to be given together on day 1 of each 2-week cycle.

Table 2: Chemotherapy dose modification in case of hematological or non-hematological toxicity

CTC grade	Percent of initial chemotherapy dose
0 - 2	100%
3 - 4	75%
3 - 4 (2nd occurrence)	50%

If a grade 3 or 4 toxicity occurs after two dose reductions, the patient is taken off treatment. If the pre-conditions for the start of a new course (according to section 6.4) are not met, treatment should be delayed up to a maximum of 3 weeks.

6.3.2.2 Specific toxicities - hematologic toxicities

In the event of any pre-cycle hematology profile identifying neutropenia and or thrombocytopenia, treatment should be delayed until the absolute neutrophil count (ANC) ≥ 1.5 × 10^9/L, platelet count ≥ 75 × 10^9/L, and recovery from non-hematological toxicity to baseline or grade ≤ 1. Treatment should then be re-initiated with the doses indicated in Table 3, Table 4, and Table 5.

Table 3: Modification of FOLFOX due to NEUTROPENIA

CTC grade	Grade 2	Grade 3	Grade 4
**ANC**	**≥ 1.0 to < 1.5 × 10^9/L**	**≥ 0.5 to < 1.0 × 10^9/L**	**< 0.5 × 10^9/L**
1st occurrence	No dose adjustment	Cytotoxic drugs at 75% of initial dose. Consider prophylactic G-CSF	Cytotoxic drugs at 50% of initial dose. Consider prophylactic G-CSF
2nd occurrence	No dose adjustment	Cytotoxic drugs at 50% of initial dose. Consider prophylactic G-CSF	Stop treatment permanently
3rd occurrence	No dose adjustment	Stop treatment permanently unless it is in the subject's best interest to continue on 5-FU/leucovorin alone + panitumumab	Not applicable

Table 4: Modifications of FOLFOX due to FEBRILE NEUTROPENIA

CTC grade	Grade 2	Grade 3
**ANC**	**< 1.0 × 10^9/L**	**< 1.0 × 10^9/L**
**Temperature**	**≥ 38.5°C**	**≥ 38.5°C**

Life-threatening sepsis	No	Yes
1st occurrence	Cytotoxic drugs at 75% of initial dose. Consider prophylactic G-CSF	Stop treatment permanently unless it is in the subject's best interest to continue on 5-FU/leucovorin alone + panitumumab. Consider prophylactic G-CSF
2nd occurrence	Stop treatment permanently unless it is in the subject's best interest to continue on 5-FU/leucovorin alone + panitumumab. Consider prophylactic G-CSF	Stop treatment permanently

Table 5: Modification of FOLFOX due to THROMBOCYTOPENIA

Platelet range	≥ 50 to < 75 × 10^9/L	≥ 25 to < 50 × 10^9/L	< 25 × 10^9/L
1st occurrence	No dose adjustment	Cytotoxic drugs at 75% of initial dose	Cytotoxic drugs at 50% of initial dose
2nd occurrence	No dose adjustment	Cytotoxic drugs at 50% of initial dose	Stop treatment permanently unless it is in the subject's best interest to continue on 5-FU/leucovorin alone + panitumumab
3rd occurrence	No dose adjustment	Stop treatment permanently unless it is in the subject's best interest to continue on 5-FU/leucovorin alone + panitumumab	Stop treatment permanently

6.3.2.3 Specific toxicities – oxaliplatin induced toxicities

Hypersensitivity reactions

Special surveillance should be ensured for patients with a history of allergic manifestations to other products containing platinum. In case of anaphylactic manifestations the infusion should be interrupted immediately and an appropriate symptomatic treatment started. Re-administration of oxaliplatin to such patients is contra-indicated. Cross reactions, sometimes fatal, have been reported with all platinum compounds.

In case of oxaliplatin extravasation, the infusion must be stopped immediately and usual local symptomatic treatment initiated.

Neurological Symptoms

Neurological toxicity of oxaliplatin should be carefully monitored, especially if co-administered with other medicinal products with specific neurological toxicity. A neurological examination should be performed before each administration and periodically thereafter.

For patients who develop acute laryngopharyngeal dysaesthesia, during or within the hours following the 2-hour infusion, the next oxaliplatin infusion should be administered over 6 hours.

Table 6 describes the recommended dose modifications of oxaliplatin based on the duration of oxaliplatin-associated neurotoxicity.

Table 6: Dose modification guidelines for oxaliplatin induced neurotoxicity

Toxicity (grade)	Duration of toxicity		Persistent (not resolved between cycles
	1 – 7 days	> 7 days	
Paresthesias/ dysesthesia of short duration that resolve and do not interfere with function (grade 1)	No change	No change	No change
Paresthesias/ dysesthesia limiting instrumental activities of daily life (grade 2)	No change	No change	Decrease oxaliplatin to 75%
Paresthesias/ dysesthesia limiting self-care activities of daily life (grade 3)	1st time: decrease oxaliplatin to 75%<br>2nd time: decrease oxaliplatin to 50%	STOP	STOP

6.3.2.4 Discontinuation of therapy

In the event that chemotherapy administration is discontinued for any reason prior to disease progression, panitumumab may continue as mono-therapy. During mono-therapy, panitumumab infusions should remain on a once every 14 days (± 2 days) schedule until the subject develops disease progression or is unable to tolerate panitumumab mono-therapy.

In the event that oxaliplatin administration is discontinued for any reason prior to disease progression, 5-FU/ FA (and panitumumab if applicable) may continue on a once every 14 days (± 2 days) schedule until the subject develops disease progression or intolerance to the therapy.

In the event that panitumumab administration is discontinued for any reason prior to disease progression, chemotherapy may continue. Chemotherapy should remain on a once every 14 days (± 2 days) schedule until the subject develops disease progression or is unable to receive chemotherapy.

6.4 Preconditions for starting a new treatment cycle

The following requirements have to be met, before a new treatment cycle may be administered:

- No hematological toxicity of grade ≥ 2 (i.e., ANC > 1,500/µL, platelets > 75,000/µL)
- No stomatitis, nausea, vomiting, diarrhea, hand-foot syndrome or other non-hematological toxicity (except for alopecia) of grade ≥ 2
- No ongoing requirement for anti-diarrheic treatment
- Bilirubin ≤ 1.5 × UNL
- Transaminases ≤ 2.5 × UNL in patients without liver metastases or ≤ 5 × UNL in the presence of hepatic lesions
- No persisting cardiac toxicity
- No peripheral neurotoxicity of grade 3
- Creatinine clearance ≥ 30 mL/min
- No treatment delay of more than 3 weeks after regularly scheduled dosing date (i.e. 5 weeks after last regular treatment)

6.5 Concomitant and supportive medication and treatment

Patients may continue their baseline medication(s). All concomitant medication(s) must be reported in the case report form (CRF). Any diagnostic, therapeutic or surgical procedure performed during the study period should be recorded, including the dates, description of the procedure(s) and any clinical findings. Patients should receive full supportive care including transfusion of blood and products, antibiotics, etc. where applicable. The treatment details should be recorded in the CRF.

6.5.1 Pre-medication

Panitumumab specific pre-medication is not required for routine panitumumab infusions. If, during or after infusion, a reaction occurs, pre-medication may be used for subsequent panitumumab infusions (e.g. acetaminophen/ paracetamol and/ or an H1 blocker, e.g. diphenhydramine).
Pre-medication may be used, when clinically indicated, at the discretion of the investigator or according to institutional and regional practice. For recommendations, please refer to the following sections.

6.5.2 Skin toxicities

The comprehensive supportive care prevention and treatment recommendations for EGFRI (epidermal growth factor receptor inhibitors) -induced dermatologic toxicities based on the pertinent literature currently available are detailed below. Recommendations are based on the results of two expert panels recently published.

Firstly, an expert panel from Germany with expertise in medical oncology, dermatology or clinical pharmacology with special knowledge of the treatment of EGFR-inhibitor-induced skin reactions was convened to develop expert recommendations based on published peer-reviewed literature. Recently published literature and data presented during the Annual meeting of the American Society of Clinical Oncology (ASCO) 2008, the World Congress of Gastrointestinal Cancer, Barcelona 2008, the European Cancer Organisation-15 Congress 2008, the ASCO GI 2009 and the ASCO 2009 served as a data source. Published clinical trials in Medline, Cochrane Library, Cochrane Controlled Trials Register, and EMBASE Drugs and Pharmacology databases were included. Information was accessed until April 2010[28].

Secondly, the Multinational Association for Supportive Care in Cancer (MASCC) Skin Toxicity Study Group assembled an international, interdisciplinary group of experts in dermatology, medical and supportive oncology, health-related quality of life (HQOL), and pharmacovigilance. Literature reviews were performed via databases such as Ovid MEDLINE (National Library of Medicine, Bethesda, MD, USA) and EMBASE (Elsevier B.V. Amsterdam, The Netherlands). Published literature as of November 2010 was included[29].

6.5.2.1 General pre-emptive skin treatment

Skin toxicity prophylaxis is performed according to the following outline at the start of therapy:

- With regard to skin care[30], patient should be advised to:
  - Use gentle soaps and shampoos (i.e., pH 5) [31-35]
  - Use lukewarm water (32-35°C) when cleansing dry skin[31]
  - Use only clean and smooth towels
  - Do not rub the skin dry after bath/ shower
  - Shave carefully
  - Moisturize skin (e.g. using water-in-oil creams e.g., Excipal U Lipolotio®), especially after washing, bathing and showering (if possible, once daily) with plenty of hypoallergenic, perfume-free moisturiser. Be careful, however, with greasy ointments in the acute phase of the acneiform eruption on the face and upper trunk as occlusion of the skin pores by ointments may aggravate the acneiform rash[31-35]

  - Use hypoallergenic sun protection of SPF 20 or higher, taking care to reapply throughout the day, as recommended, when exposed to sun[31,32,34,36]
  - Keep hands dry and out of water if possible
- With regard to nail care, patients should be advised to:
  - Clean nails, and apply hypoallergenic moisturising creams or lotions to the hands and feet
  - Do manicure straight across
  - Do not trim cuticles
  - Avoid friction and pressure on the nails, i.e., do not wear tight shoes
  - Seek podiatric advice prior to initiation of EGFRI treatment (especially in cases of ingrown nails or other situations predisposing to paronychia)
- With regard to clothing, patients should be advised to:
  - Wear hat and protective clothing for sun protection[35]
  - Wear rubber or cotton-lined gloves when washing dishes or cleaning[30]
  - Avoid tight-fitting shoes
  - Wear slippers or house shoes

6.5.3 Preventive and treatment recommendations for specific clinical symptoms of the skin

After onset of dermatologic reactions, treatment should be started as early as possible.

Treatment of papulopustular (acneiform) rash

<u>Prevention</u>

Within the first 2-4 weeks EGFRI-treated patients frequently develop a typical papulopustular (acneiform) rash. Therefore, preventive/prophylactic management is recommended unless there are contraindications based on patient and/or health care provider factors[29]. Skin care, moisturizing of the skin and sun protection may be useful as described in the general treatment recommendations[37,38].

Prophylactically doxycycline 100 mg bid for the first 6 weeks of treatment is recommended[38]. The safety profile of doxycycline appears to be advantageous, especially in patients with renal dysfunction, however, it increases the skin photosensitivity.

<u>Treatment</u>

For early stage and low grade papulopustular skin reactions should be a topical antibiotic treatment started with metronidazole 2%[39].
If a moderate acneiform rash occurs after the first 6 weeks of treatment doxycycline 100 mg bid should be re-induced.

Secondarily infected rash

Secondary infection of an acneiform skin rash may occur at later stages, which includes impetiginisation - an important complication caused by staphylococci or streptococci. In addition, abscesses may require incision and drainage to prevent sepsis. Bacterial swabs should be taken and calculated anti-infective treatment should be started by a dermatologist, which may include oral or intravenous antibiotics. *Staphylococcus aureus* is the most frequently detected relevant infectious agent detected in EGFR inhibitor-induced skin lesions[40].

The papulopustular (acneiform) rash is most pronounced in weeks 4-6 after EGRFI initiation and the severity of skin toxicity decreases after 6-8 weeks. However, post-inflammatory skin alterations occur as erythema and hyperpigmentation that may exist for months or years[29].

Therefore, prophylactic strategies are important, and appropriate medication should be considered throughout EGFRI treatment and follow-up in order to minimize these late effects.

Pruritus/ itching

Preventive

Both preventive skin care measures as well as treatment of rash may reduce pruritus. Furthermore, it is useful to nurse dryness of the skin because itching can also occur as a consequence of dry skin. Therefore, skin moisturizers (e.g., Deep Moisture Bodylotion Sensitive by Neutrogena ®) and urea-containing lotions (e.g., Eucerin ® TH 10% Urea Lotion) are useful.

Treatment

As first approach for systemic treatment, non-sedating second-generation antihistamines (loratadine e.g., Loratadin STADA ® 10 mg) are recommended for pruritus during daytime[41-43]. The recommended dose for loratadine for adults is 10 mg once daily.

Since antihistamines may result in severe anticholinergic reactions, especially in elderly patients, caution should be exercised when dosing. The dosage should therefore be kept to a minimum.

Because of the sedative properties of the first-generation antihistamines (diphenhydramine and hydroxyzine e.g., Dolestan ® 25 mg and ATARAX ® 25 mg) should be recommended in

patients who suffer from itching especially at night[44] The dosage for adults for diphenhydramine or hydroxyzine is 25 mg once for night.

Treatment for xerotic and eczematous skin

Preventive

Bathing techniques are already preventive. Lukewarm water for bathing should be used. Patients should be advised to use bath oils or mild moisturizing soaps that are free of fragrances or perfumes.

Extreme temperatures such as severe cold, dry weather or significant heat should be avoided. Likewise, patients should not exposure direct in the sun because of the risk of sunburns.

Patients should not use alcohol- containing lotions or skin products that may dehydrate skin[34,45].

Treatment

Mild or moderate xerosis should be treated with thick moisturizing creams without fragrances or potential irritants (e.g., Neutrogena ® Intense Repair Bodybalsam). Urea may be included in specific creams (e.g., Eucerin ® TH 10% Urea Lotion).

Moisturizers should be occlusive, emollient creams that are generally packaged in a jar or tub rather than a lotion that can be pumped or poured. Greasy creams may be used on the limbs for better control of xerosis but are cautioned on the face and chest, along with extremely hairy sites, due to risk for folliculitis secondary to occlusion[34].

Fissures

Preventive

Protective footwear should be worn or fingertips should be covered to avoid friction, to prevent skin fissures and to aid healing. Thick moisturizers (e.g., Neutrogena® Intense Repair Bodybalsam) or zinc oxide creams (e.g., Pantederm® N HEXAL® 10% Salbe) can be applied.

Treatment

Medical skin adhesives like cyanoacrylate preparations (e.g., Epiglu® (Etyhl-2-Cyanoacrylat, Fa. Meyer-Haake, Oberursel) and Histoacryl® (Butyl-2-Cyanoacrylat, Fa. Braun, Melsungen) can be used to seal the cracks and keep them from worsening or becoming infected[46,47]. Dermabond ® (octyl-2-cyanoacrylate, Ethicon) is available as a new skin adhesive. Sealing

the cracks with these products can also help relieve pain and allow for healing[46]. Oral antibiotics may be necessary if infection worsens despite topical treatment[29].

Paronychia

Preventive

Paronychia carries the risk of a super infection. For prevention of periungual trauma, comfortable shoes should be worn, nails should be cut short without aggressive manicure, and gloves should be worn while cleaning (e.g., household, dishes)[29].

Treatment

To prevent bacterial superinfection antimicrobial soaks (e.g. chlorhexidine, povidone-iodine) are recommended[29].

Culturing of lesional skin to determine if superinfection is present is universally recommended so that antimicrobials can be directed accordingly. Bacterial swabs should be taken and anti-infective treatment should be started by a dermatologist, which may include oral or intravenous antibiotics.

For severe conditions of papulopustular (acneiform) rash, pruritus, xerosis, fissures or paronychia a consultation of a dermatologist is warranted. A summary of preventive and treatment recommendations for dermatological symptoms is listed in table 7.

Table 7: Preventive and treatment recommendations for dermatological symptoms

Skin toxicity	Rash	Pruritus	Xerosis	Fissures	Paronychia
Prevention	Topical<br>- Moisturizer twice daily<br>- Sunscreen twice daily<br><br>Systemic<br>- Doxycyline 100 mg bid	Topical<br>- Moisturizer twice daily<br>or<br>- Urea-containing lotions	Topical<br>- Tepid water<br>- Bath oils or mild moisturizing soaps<br>- Regular moisturizing<br><br>CAVE no alcohol-containing lotions	Topical<br>- Protective footwear<br>- Avoid friction with fingertips, toes and heals	Topical<br>- Comfortable shoes<br>- Short nails<br>- Gloves while cleaning
Treatment	Topical for CTC grade 1<br>- Metronidazole cream 2% once for night<br><br>Systemic for CTC grade ≥ 2<br>- Doxycyline 100 mg bid	Systemic<br>- Antihistamines<br>First approach: Loratadine 10 mg once daily<br><br>Itching at night: Diphenhydramine 25 mg once at night	Topical for CTC grade ≥1<br>- Thick moisturizing creams<br>or<br>- Urea-containing lotions<br>- Greasy creams for limbs, CAVE not for face, chest and extremely hairy sites	Topical for CTC grade ≥1<br>- Thick moisturizers<br>or<br>- Zinc oxide creams<br>- Medical skin adhesives	Topical for CTC grade 1<br>- Antimicrobial soaks<br><br>Systemic for CTC grade ≥ 2<br>- Antimicrobials reserved for culture proven infection
	Bacterial swabs should be taken and calculated anti-infective treatment started		Bacterial swabs should be taken and calculated anti-infective treatment started	Bacterial swabs should be taken and calculated anti-infective treatment started	

6.5.4 Treatment of nausea/ vomiting

For acute nausea and vomiting, a 5-HT3 antagonist with corticosteroids prior to infusion would be considered standard premedication. For delayed nausea and vomiting, an oral 5-HT3 antagonist is the first option; metoclopramide, alizapride and prochlorperazine may be also used.

6.5.5 Treatment of diarrhea

Symptoms of diarrhea and/or abdominal cramping may occur at any time and should be managed according to standard institutional practice.

Subjects should also be instructed to notify the investigator or nurse for the occurrence of bloody or black stools, symptoms of dehydration, fever, inability to take liquids by mouth, inability to control diarrhea (return to baseline) within 24 hours. Subjects with diarrhea should be evaluated frequently by a nurse or physician until resolution of diarrhea.

Changes in electrolytes, even without BUN/urea and/or creatinine elevation, may reflect early physiologic consequences of treatment-induced gastrointestinal toxicity. Subjects with clinically significant electrolyte changes should be evaluated for dehydration and receive aggressive fluid and electrolyte replacement, if indicated.

A prophylactic treatment is not recommended. As soon as signs of diarrhea occur, the patient should immediately consult his physician and start with the intake of loperamide: 2 capsules (4 mg), thereafter 1 capsule (2 mg) every two hours, for at least 12 hours and for at least 12 hours after the last observation of liquid stool. The use of drugs with laxative properties should be avoided because of the potential for exacerbation of diarrhea. Patients should be advised to contact their physician to discuss any laxative use. Sufficient oral rehydration has to be administered during the whole diarrhea episode.

If diarrhea is severe (requiring intravenous rehydration) and/or associated with fever or severe neutropenia (Grade 3 or 4), broad-spectrum antibiotics must be prescribed. Patients with severe diarrhea or any diarrhea associated with severe nausea or vomiting **must be hospitalized** for intravenous hydration and correction of electrolyte imbalances. In case of persisting severe diarrhea despite loperamide treatment, this drug should be replaced by another antidiarrheal therapy (e.g. octreotide).

6.5.6 Electrolyte management

Subjects should be evaluated as outlined in section 5 and managed as per local medical practice. If hypomagnesemia is present, replacement should be managed with either oral or parental replacement, or both, according to institutional practice and to the degree of hypomagnesemia present. It is recommended that subject’s serum magnesium level should be maintained within the normal range during study treatment.

It is important to assess and manage serum potassium and calcium (adjusted for albumin) in subjects who have concomitant hypomagnesemia. Subject's serum potassium and calcium parameters are recommended to be maintained, as per local medical practice, within the normal ranges during study treatment.

6.5.7 Prevention and treatment of neutropenia

Hematopoietic growth factors (i.e., filgrastim or pegfilgrastim) may be used according to institutional or other specific guidelines (e.g. ASCO, EORTC) to treat febrile neutropenia. Due to the high risk of neutropenia together with gastrointestinal toxicity, secondary prophylactic use of G-CSF should be considered after the first chemotherapy associated episode of neutropenia > grade 2, especially in patients >60 years.

Use of any supplementary growth factor must be documented in the patient record. Growth factors must be discontinued at least 48 hours prior to initiation of the next cycle of chemotherapy.

Prophylactic treatment with antibiotics is not allowed.

6.5.8 Prohibited Medications

Patients should not receive any of the following medications during the treatment period:

- any other antineoplastic treatment
- any other medication for skin toxicity *prophylaxis*
- antibiotics for prophylaxis of hematological toxicity
- other investigational therapies

7 Assessment of Safety

The Investigator’s Brochure will be used as reference document for panitumumab and will be provided to the investigators in the investigator’s file.

7.1 Definitions of Adverse Events and Serious Adverse Events

7.1.1 Adverse Event

An adverse event is defined in the International Conference on Harmonization (ICH) Guideline for Good Clinical Practice as “any untoward medical occurrence in a patient or clinical investigation subject administered a pharmaceutical product and that does not necessarily have a causal relationship with this treatment” (ICH E6:1.2).

An adverse event can therefore be any unfavorable and unintended sign (including an abnormal laboratory finding, for example), symptom or disease temporally associated with the use of a medicinal product, whether or not it is considered related to the medicinal product.

7.1.2 Adverse Drug Reaction

All untoward and unintended responses to a medicinal product related to any dose administered.

The phrase “responses to a medicinal product” means that a causal relationship between the medicinal product and the adverse event is at least a reasonable possibility, i.e., the relationship cannot be ruled out.

A serious ADR (SADR) is an ADR that meets the definition of serious (provided below).

7.1.3 Serious Adverse Event

A serious adverse event (SAE) is defined as an adverse event that

- is fatal
- is life threatening (places the subject at immediate risk of death)
- requires in-patient hospitalization or prolongation of existing hospitalization
- results in persistent or significant disability/incapacity
- is a congenital anomaly/birth defect
- other significant medical hazard

For this study, the following is **not** classified as serious adverse event:

- Progression or deterioration of the malignancy under study (including new metastatic lesions) or death due to progression unless the investigator deems it to be related to the use of study treatments.

- Hospitalizations for the performance of protocol-required procedures or administration of study treatment
- Hospitalizations or procedures planned prior to study start or elective hospitalizations

A hospitalization meeting the regulatory definition for “serious” is any inpatient hospital admission that includes a minimum of an overnight stay in a healthcare facility. Any adverse event that does not meet one of the definitions of serious (e.g., visit to A&E, outpatient surgery, or requires urgent investigation) may be considered by the investigator to meet the “other significant medical hazard” criterion for classification as a serious adverse event. Examples include allergic bronchospasm, convulsions, and blood dyscrasias.

7.1.4 SUSAR

A SUSAR is defined as a suspected unexpected serious adverse reaction. An unexpected adverse event is any adverse drug event, the specificity or severity of which is not consistent with the current investigator’s brochure for panitumumab. Also, reports that provide significant information on the specificity or severity of a known, already documented adverse event constitute unexpected adverse events. An event more specific or more severe than described in the investigator’s brochure would be considered “unexpected”. All suspected adverse reactions related to panitumumab which occur in the trial and that are both unexpected and serious (SUSARs) are subjectFto expedited reporting.

7.2 Reporting of SAEs

Any clinical adverse event or abnormal laboratory test value that is serious occurring during the course of the study from the date of signing the informed consent, irrespective of the treatment received by the patient, must be reported to the sponsor within 24h of knowledge (expedited reporting) per European Union Guidance CT3 (R2). For each patient, all serious adverse events should be reported up to 30 days after the last dose of investigational product. Serious adverse events with relation to the IMP occurring more than 30 days after a patient is discontinued from the study treatment must be reported to the Sponsor.

The completed SAE form must be faxed to:

ClinAssess GmbH
Birkenbergstr. 82, 51379 Leverkusen
Fax: +49 (0) 2171 / 36 336 55

The sponsor will medically review all SAEs.

It is possible that Amgen may request follow-up information via the sponsor.

The following detailed information must be recorded for each serious adverse event in the SAE report form:

- A description of the AE in medical terms preferably according to NCI-CTCAE Version 4.03, not as reported by the subject
- The severity grade as assessed by the investigator according to the definitions in NCI-CTCAE Version 4.03
- The date of becoming serious and the date of becoming known (if different)
- The reason for seriousness
- The outcome of the SAE at the time of the report
- Information on administration of the study drug and chemotherapy and any action taken
- Information on any treatment procedures necessary for the SAE, concomitant medications, relevant lab tests and relevant medical history

If in any one subject the same SAE occurs on several occasions, then the SAE in question must be documented and assessed anew each time.

The investigator is required to submit SAE Follow-up reports until the SAE has resolved or stabilized and all queries have been answered.

7.3 Reporting of SUSARs

The sponsor will ensure the notification of the appropriate ethics committees, competent authorities and participating investigators of all SUSARs events occurring at the sites in accordance with local legal requirements, statutes and the European Clinical Trial Directive as follows:

- Reporting of the SUSAR to the Competent Authorities and Ethics Committees within 15 days (or within 7 days for fatal and life-threatening events)
- Sending the event to all participating Investigators for information (with confirmation of receipt).

In addition, all events that require a new assessment of the risk-benefit ratio will be reported to the Ethics Committee and the Competent Authority of each concerned Member State within 15 days. This includes:

- Single reports of expected serious adverse reactions with unexpected outcome.
- An increase in the rate of occurrence of expected serious adverse reactions which is judged to be clinically relevant
- Post-study SUSARs that occur after the patient has completed a clinical trial

- New events related to the conduct of the trial or the development of the investigational medicinal products and likely to affect the safety of the subjects

The sponsor is responsible to ensure that the latest investigator's brochure is used as the source document for determining the expectedness of an SAE.

7.4 Recording of Adverse Events

The investigator is responsible for ensuring that all adverse events observed by the investigator or reported by subjects are properly recorded in the subjects' medical records and the case report form.

The following adverse event attributes must be assigned by the investigator:

- Adverse event term according to the NCI-CTCAE criteria Version 4.03
- Severity grade according to the NCI-CTCAE criteria Version 4.03
- Start date and stop date (or date of last assessment)
- Outcome
- Causality to study drug and chemotherapy (to be assessed as either related or unrelated)
- Any action taken

Adverse events will be followed until they resolve to baseline or considered stable. It will be left to the investigator's clinical judgment to determine whether an adverse event is related and of sufficient severity to require the subject's removal from treatment or from the study. A subject may also voluntarily withdraw from treatment due to what he or she perceives as an intolerable adverse event. If either of these situations arises, the subject should be strongly encouraged to undergo an end-of-study assessment and be under medical supervision until symptoms cease or the condition becomes stable.

Pre-existing diseases, when worsening during study therapy, have to be considered as adverse events. They can lead to serious adverse events, if they meet the criteria described in section 7.1.3.

Intensity of adverse events will be graded using the National Cancer Institute Common Terminology Criteria for Adverse Events (CTCAE), version 4.03 (see investigator's file). If an adverse event occurs which is not described in the CTCAE version 4.03, the four-point scale below will be used.

Mild:	Discomfort noticed but no disruption of normal daily activity
Moderate:	Discomfort sufficient to reduce or affect daily activity
Severe:	Inability to work or perform normal daily activity

Life-threatening: Represents an immediate threat to life

7.5 Laboratory Test Abnormalities

Laboratory test results will be recorded on the laboratory results pages of the case report form. In the event of unexplained abnormal laboratory test values, the tests should be repeated immediately and followed up until they have returned to the normal range and/or an adequate explanation of the abnormality is found.

Laboratory test value abnormalities as such should not be reported on the AE page of the CRF as adverse events unless they are treatment-emergent and they satisfy one or more of the following conditions for clinical significance:

1. Accompanied by clinical symptoms
2. Leading to a change in study medication (e.g., dose modification, interruption or permanent discontinuation)
3. Requiring a change in concomitant therapy (e.g., addition of, interruption of, discontinuation of, or any other change in a concomitant medication, therapy or treatment)

Please note: Any laboratory result abnormality fulfilling the criteria for a serious adverse event (SAE) should be reported as such, in addition to being recorded as an adverse event in the CRF.

7.6 Pregnancy

Female patients must be instructed to immediately inform the investigator if they become pregnant during the study. The study treatment must immediately be stopped. Pregnancies occurring up to 6 months after the completion of the last treatment cycle must also be reported to the investigator. The investigator must report all pregnancies within 24 h to ClinAssess. ClinAssess will forward all pregnancy reports to the sponsor within one working day. The investigator should counsel the patient; discuss the risks of continuing the pregnancy and the possible effects on the fetus. The patient should be monitored until the conclusion of the pregnancy. Follow up case reports have to be provided as new information becomes available.

Pregnancy occurring in the partner of a patient participating in the study should also be reported to the investigator, the sponsor and the CRO. The partner should be counseled and followed as described above.

7.7 Adverse Drug Reactions with Concomitant Medication

The investigators must be aware that for all concomitant medications the regulations of post-marketing reporting for suspected adverse drug reactions apply, i.e., reporting to the marketing authorization holder or the local regulatory bodies.

8 Biostatistical Aspects

8.1 Trial Design and Hypotheses

This trial is designed as a randomized, parallel-group, open label, multicentre, phase II trial with formal "within-trial" comparison in order to show any beneficial effect of maintenance plus panitumumab compared to maintenance alone in terms of progression-free survival. The following hypothesis system will be subjected to statistical analysis:

Null Hypothesis H_0: HR= 1 vs. Alternative Hypothesis H_1: HR< 1,

where HR denotes the ratio of the hazard of progression/death with arm A divided by the hazard of progressions/death with arm B.

8.2 Sample Size Calculation

The sample size required within this trial is calculated based on the hypothesis system described in section 8.1. To estimate the number of events (progressions or deaths, whichever first) required within the Full Analysis Population in this study, the following assumptions are made:

- One-sided Log-rank test will be applied for comparison of PFS between treatment arms.
- The one-sided significance level is set to 10%. This reflects the goal of the phase II to
  - allow for unbiased evaluation of any potential superiority of 5-FU/FA plus panitumumab compared to 5-FU/FA alone
  - generate sufficient data for preliminary assessment of the clinical relevance of the maintenance treatment with 5-FU/FA plus panitumumab
  - provide a sensible gate-keeper for further development and
  - support further decision making within the community and
  - last but not least decide whether a Phase III trial is justified.
- Progression-free survival follows an exponential distribution.
- Median PFS in patients treated with maintenance lacking panitumumab is expected to be roughly 7.5 months. This is derived by considering OPTIMOX2 showing a median PFS of 8.6 months in patients treated with mFOLFOX7 during induction followed by simplified LV5FU2 therapy thereafter. Excluding the first 3 months of induction therapy and excluding progressions and other cases where patients are not eligible for randomization after 3 months which do not start maintenance therapy, will lead to a median progression-free survival (calculated from date of randomisation) of roughly 7.5 months.

- On the other hand, previous phase III studies (e.g., PRIME-study) have shown that adding panitumumab can lead to prolongation of PFS with hazard ratios (HR) ranging between 0.7 and 0.8. Based on this data, 5-FU/FA+panitumumab will be considered to be clinically relevant if it is associated with a HR of 0.75; this converts into a median PFS of 10 months.
- The power to yield statistical significance if the HR is in fact 0.75 is set to 80%.
- All patients are to be followed until progression or death whatever occurs first. 5% of patients will be assumed to be continuously lost during the study period per year.

Based on the asumptions above, a logrank test for comparing the progression-free survival curves will have 80% power to reject the null-hypothesis if the true hazard ratio is 0.75 with a total number of 218 events (progressions or deaths, whichever occurs first).

Since the power of any time-to-event study is determined by the number of events rather than the number of patients, there is a range of sample sizes that meet the objectives of this trial. Naturally, the more patients being followed, the sooner the desired number of events will be observed. Assuming that with roughly 9 patients starting the induction phase, 8 patients per month can actually be randomized thereafter, the required number of events will be expected to occur within a total study duration (including follow-up) of roughly 108 months with at most 252 patients qualifying for Full Analysis population. To account for the fact that merely roughly 90% of patients treated with mFOLFOX6+panitumumab will show CR, PR or SD, but a relevant drop-out for various reasons (includung patients with secondary resections who were therefore not eligible for randomisation) occurs after induction therapy, a total of approx. 400 patients should be enrolled to the trial to ensure that 252 patients enter the randomisation and receive maintenance treatment.

If a more rigid significance level of one-sided 5% is required, the above mentioned sample size allows demonstrating statistical significance with a power of roughly 69%. However, if the true HR is 0.714 or less (i.e., if the median PFS associated with 5-FU/FA+panitumumab is at least 10.5 months), statistical significance at the 5% significance level can be ensured with a power of 80%.

8.3 Evaluation Categories for Patients

8.3.1 Full Analysis Population

The Full Analysis Population is used to perform the Intent-to-Treat (ITT) analysis and includes all randomized patients who received at least one dose of maintenance treatment (irrespective of substance) according to the study protocol. Patients within the ITT will be analyzed in their initial group of randomization.

Furthermore, the Full Analysis Population for the whole study, the induction phase and the maintenance phase will be defined, including all patients who received at least one dose of study treatment in the respective study phase.

8.3.2 Per Protocol Population

The Per Protocol Population (PP) will comprise only patients of the Full Analysis Population (only for maintenance phase) while excluding patients if any of the following criteria are met:

- Administration of less than two cycles of maintenance treatment if the reason for discontinuation was any other than death, progression of disease or continued unacceptable toxicities despite optimal treatment or dose reduction
- Any violation of major inclusion criteria or major exclusion criteria.
- Other major protocol violations such as wrong treatment received.
- Lack of any efficacy data after start of maintenance treatment.

Major violations will be determined independently of knowledge of response to therapy. A data review will be conducted prior to database lock based on all data to review protocol deviations, to discuss specific unforeseeable data issues and to allocate the subjects to the analysis sets. The associated decisions are documented and approved in the Data Review Meeting protocol. Protocol violations resulting in an exclusion from the Per Protocol Population will be described in the Clinical Study Report. All patients in the PP will be analyzed within their group of actual treatment received.

8.3.3 Safety Population

The Safety Population consists of all randomized patients who started maintenance treatment. All patients will be analyzed within their group of actual treatment received.

Furthermore, an additional induction therapy specific safety population will be defined to allow analyzing toxicity of induction therapy. All enrolled patients who started induction will be included.

Patients who cannot be included in any of the above mentioned analysis populations will be excluded from the statistical analyses for the respective population. Any recorded data of these patients will be listed in the appendix to the Clinical Study Report.

8.4 Methods of Statistical Analysis

8.4.1 General Statistical Considerations

The statistical evaluation will be carried out by ClinAssess GmbH. Statistical analysis is based on the International Conference on Harmonization (ICH) Guidelines “Structure and Content of Clinical Study Reports” and “Statistical Principles for Clinical Trials”.

Data will be analyzed using the SAS software package version 9.4 or higher on the Windows platform.

Study-specific SAS programs will be written for individual patient data listings and for aggregated analyses of adverse events and laboratory values. The biometrical analysis will be accompanied by the appropriate internal quality control according to ClinAssess’s current SOPs. The data analysis will be performed according to the statistical analysis plan (SAP) which will be finalised prior to database lock and prior to any statistical analysis. Any changes to the statistical analysis planned in the protocol are to be justified and documented in the SAP if they were decided before database lock. Any changes made to the SAP after the database lock will be documented in the clinical study report.

All data recorded in the case report forms describing the sample, the efficacy and the safety will be analyzed descriptively. Categorical data will be presented in contingency tables with frequencies and percentages. Continuous data will be summarized with at least the following: frequency (n), median, quartiles, mean, standard deviation (standard error), minimum and maximum. Number of patients with protocol deviations during the study and listings describing the deviations will be provided.

In general, chi-square tests will be used to compare percentages in a two-by-two contingency table, replaced by Fisher´s exact test if the expected frequency in at least one cell of the associated table is less than 5. Stratified two-by-two contingency tables will be analyzed using Cochran-Mantel-Haenszel tests. Comparison of ordinal variables between treatment arms will be performed using the asymptotic Wilcoxon-Mann-Whitney test, replaced by its exact version in case of ordinal categories with small number of categories and/or sparse data within categories. Any shift in location of quantitative variables between study groups will be performed with Wilcoxon-Mann-Whitney tests as well.

Time-to-event data will be analyzed by Kaplan-Meier methods, when merely non-informative censoring occurs. For statistical comparison, the logrank-test will be provided supplemented by multivariate Cox proportional hazards models.

The primary endpoint of this trial will be subjected to confirmatory statistical hypothesis testing applying the one-sided significance level of 10%. All other statistical tests are to be interpreted on an exploratory perspective. Consequently, no adjustment for multiple testing is foreseen.

Confirmatory statistical analysis of the primary efficacy parameter will be performed within the Full Analysis Population (Intent-to-Treat analysis). The Per Protocol Population merely serves for sensitivity analysis. All safety analyses will be performed within the Safety Population.

8.4.2 Demographics and Baseline Characteristics

All demographic and clinical characteristics recorded at baseline will be submitted to descriptive analyses using descriptive statistics by means of listing, tables and figures, if applicable. This analysis will be performed for all patients enrolled to the trial and subsequently for all patients randomized. The latter analysis will be stratified by treatment arms to assess homogeneity.

8.4.3 Efficacy Evaluation

Efficacy of induction phase

To describe the efficacy of induction phase all patients enrolled to the trial with documented start of induction therapy will be subjected to descriptive analysis.

- *Objective response after 12 weeks of induction chemotherapy*

  Objective response after 12 weeks is defined as the objective response, i.e. CR or PR, measured at the tumor assessment 12 weeks after start of induction. Patients without any documented tumor assessment are considered to have experienced a progressive disease, in case of therapy discontinuation due to progressive disease or early death (defined as death prior to first scheduled tumor assessment after start of treatment). For statistical analysis, the distribution of best response categories will be provided based on all patients who have started induction phase.

- *Overall Survival*

  For exploratory purposes, the overall survival experience between start of induction therapy and date of randomization will also be subjected to statistical analysis. This analysis will comprise all patients who have started induction phase.

Primary efficacy parameter (Progression-free survival)

The primary efficacy parameter of the trial is progression-free survival (PFS). PFS will be defined as the time length between the date of randomization and the date of first disease

progression (according to RECIST criteria V1.1, see appendix 4) or death (whichever occurs first).

Patients alive without first objective disease progression will be censored at the last time known to be alive and without known objective disease progression

First of all, the distribution of progression-free survival time will be described using Kaplan-Meier methods. These analyses will be presented by treatment arm and stratified by prognostic risk groups used at randomization. Estimates of median progression-free survival and estimates of progression-free survival rates for specific time points will be extracted from the Kaplan-Meier analyses together with the associated 80% confidence limits.

For confirmatory testing, the difference in progression-free survival between the two treatment arms according to the hypothesis system defined in section 8.1, a stratified logrank test will be applied. The stratification factors of response to induction therapy (CR/PR vs. SD), prior oxaliplatin-containing adjuvant therapy (yes vs. no) and planned dosage of maintenance therapy (full dosage of panitumumab vs. reduced dosage of panitumumab) will be applied. The one-sided significance level is predefined as 10%. Furthermore, stratified Cox regression model will be used to estimate the hazard ratio. For sensitivity analysis, a multivariate Cox proportional hazards model will also be fitted using the stratification factors at randomization as covariates in addition to the treatment parameter.

Secondary efficacy parameter (Time from randomization until failure of treatment strategy)

Time from randomization until failure of treatment strategy is defined as the time from randomisation to second objective disease progression, or death from any cause, whichever first. Patients alive and for whom a second objective disease progression has not been observed will be censored at the last time known to be alive and without known second objective disease progression. A sensitivity analysis for this efficacy parameter will be performed to account for those patients who do start re-induction therapy despite of lack of first objective progression and for patients who do not start re-induction despite of being alive and having a first objective progression during maintenance. These patients will be censored at the time of start of re-induction and the date of first objective progression, respectively.

The distribution of the time to failure of treatment strategy will be described using Kaplan-Meier methods. These analyses will be stratified by treatment arm and prognostic risk groups used for randomization. Estimates of median time to failure of treatment strategy and estimates of failure rates for specific time points will be extracted from the Kaplan-Meier analyses together with the associated 80% confidence limits.

The difference in time to failure of treatment strategy between the two treatment arms will be tested using a stratified logrank test as already described for progression-free survival above.

Furthermore, Cox proportional hazards models as already described for progression-free survival will be applied to yield adjusted estimates of the associated hazard ratios.

Secondary efficacy parameter (Progression-free survival of re-induction)

Progression-free survival of re-induction will be defined as the time length between the date of objective disease progression during maintenance and the date of first disease progression (according to RECIST criteria 1.1, see appendix 4) or death after start of re-induction (whichever occurs first). The Subgroup of patients with progression during/following maintenance and start of re-induction will be considered for this analysis. Patients alive with no documented progression during re-induction will be censored at the last documented visit without known progression of disease. Exploratory statistical analysis will consist of Kaplan-Meier analyses and Cox regression modelling.

Secondary efficacy parameter (Overall survival)

Overall survival will be defined as the time length between date of randomization and the date of death from any cause or the date of last follow-up in case of no documentation of death. All reported deaths occurring after randomization will be included, regardless whether they occur in maintenance, re-induction or following treatment discontinuation. Statistical analysis of overall survival will be conducted in analogy to the statistical analysis of the primary efficacy parameter.

Overall survival will also be analysed as the time length between date of registration (enrolment) and the date of death from any cause or the date of last follow-up in case of no documentation of death. All reported deaths occurring after registration/enrolment will be included, regardless whether they occur in induction, maintenance, re-induction or following treatment discontinuation. Statistical analysis of overall survival will be conducted in analogy to the statistical analysis of the primary efficacy parameter.

Secondary efficacy parameters (objective best response during maintenance and re-induction)

Best objective response within maintenance (comparison randomization image to assessements during maintenance) and re-induction phase (comparison of progression image during maintenance to images during re-induction phase) is defined as the best response documented within each specific therapy phase. Patients without any documented tumor assessment within the respective therapy phase are considered to have experienced a progressive disease, in case of therapy discontinuation due to progressive disease or early death (defined as death prior to first scheduled tumor assessment within each treatment phase). The distribution of best response categories within different treatment phases will be described.

For statistical comparison of treatment arms with respect to best response during maintenance and best response during re-induction, patients experiencing PR or CR will be considered as

responders. Point estimates of responder rates, the estimated difference in response rates and the associated 95% confidence intervals will be provided. The Cochran-Mantel-Haenszel chi-square test stratified by the same factors as for the analysis of progression-free survival will be used to compare treatment arms. An unadjusted Chi-square test result will also be provided.

Secondary parameter (Quality of Life)

Quality of life will be evaluated using the validated EORTC QLQ-C30, FACT-EGFRI 18, Skindex-16 and DLQI questionnaires. Data will be scored according to the algorithm described in the respective scoring manuals. For all quality of life domains and items, descriptive analyses will be presented stratified by visit and treatment arm.

Statistical comparison of quality of life between treatment arms may be distorted due to large quantities of missing questionnaires. Thus, the extent and process of missing data will be investigated. In case that data are missing completely at random, complete case analyses will be applied without biasing the results; otherwise treatment arms will be compared using longitudinal data modeling techniques.

For descriptive statistical analysis, summary tables will be provided showing measures of location and dispersion (minimum, quartiles, median, maximum, mean and standard deviation) stratified by visit and treatment arm. Furthermore, individual score items will be subjected to statistical analysis. Absolute changes of QoL-scores from baseline will be tabulated stratified by treatment group and visit. For graphical illustrations, Box-Whisker diagrams will be presented across visits for each treatment group.

For each domain, the proportion of patients in each arm with ≥ 10 points deterioration at each visit will be compared. It has been shown that this degree of change is perceptible and clinically significant on the 100 point scale. The numbers and percentages of patients with deterioration (≥10 points deterioration), no change (less than 10 points deterioration/improvement) and improvements (≥10 points improvement) will also be presented. Nonparametric (exact) Wilcoxon-Mann-Whitney tests will be applied for exploratory comparison purposes. In order to account for non-ignorable missing values due to death, progression or lost to follow-up, supplementary time profile analyses relative to baseline will be performed consisting of conditional descriptive analyses, given QoL-data up to a certain visit x, i.e. QoL tables at a specific visit x will be presented for patients with EOT-visit at or after visit x only.

In addition, time-to-deterioration-analyses will be conducted for assessing any long term differences between treatment arms. Patients will be considered to have deteriorated for a given domain if a deterioration of at least 10 points on the 100 point scale is observed after randomization. Patients without deterioration will be censored at the time of the last QoL assessment. Kaplan-Meier methods will be used for associated data descriptions. For

comparison purposes, logrank tests will be applied. Additional details of the QoL analysis will be described in the SAP.

Exploratory efficacy parameter (Translational Research)

Tumor tissues, circulation tumor cells, skin biopsies and blood cells and other blood based markers such as cell-free DNA and proteins or immune-associated molecules will be characterized. The biomarker expressions are planned to be correlated with tumor staging and response to therapy in both treatment arms. Available details of the translational analysis will be laid down in the SAP prior to performing the analysis.

Translational research analysis will be conducted by members of the translational research committee outside of ClinAssess.

8.4.4 Safety Evaluation

Adverse events

Adverse event reported terms (as documented by the study investigator) and coded terms will be reviewed and re-coded if necessary. All information recorded such as onset date, stop date, duration, maximum intensity, seriousness, relationship to study drug and outcome will be listed.

Adverse event tables will be created. These tables will present the total number of patients reporting at least one specific event and the maximum CTCAE grade. Thus, patients reporting more than one episode of the same event will be counted only once by the worst CTCAE grade per patient.

Detailed adverse event tabulations consist of the number and percentages of patients involved per CTCAE Category and CTCAE Term, the highest relation to study drug, and the maximum severity.

Special tables will be displayed for CTCAE Grade III/IV/V adverse events. Additionally, analysis will be restricted to drug related adverse events and drug related CTCAE Grade III/IV/V events.

Furthermore, summary tables with patient identifications will be presented. These tables provide the numbers and percentages of patients with adverse events stratified by CTCAE category, CTCAE term and CTCAE grade, and also include the subject identification in the table.

Laboratory parameters

Quantitative laboratory parameters will be evaluated stratified by treatment arm and visit. For each parameter, the distribution over time as well as the mean values at baseline and changes from baseline will be computed for each treatment arm and reported with descriptive statistics.

Values outside normal ranges will be listed. Any statistical test will be interpreted on an exploratory perspective only. Laboratory parameters to be included in this analysis will be detailed in the statistical analysis plan.

Safety analyses will be conducted for the Safety Population. However, additional tables showing the incidence of adverse events during induction therapy will be presented for all patients enrolled with documented start of induction therapy.

8.5 Interim and Final Analysis

No confirmatory interim analyses for efficacy with the aim to stop the trial prematurely due to proof of efficacy or futility are foreseen within the study protocol.

The primary confirmatory statistical analysis of this trial is performed whenever at least 218 events (first progressions/deaths) are documented within the Full Analysis population. The final analysis will be performed with a minimum follow-up of 2 years after randomization of the last subject available for each patient randomized to the trial.

9 Data Quality Assurance

The overall procedures for quality assurance of clinical study data are described in the Standard Operational Procedures of ClinAssess. Accurate and reliable data collection will be assured by verification and cross–check of the electronic CRFs against the investigator's records by the study monitor (source document verification), and the maintenance of a drug–dispensing log by the investigator. Data for this study will be recorded via electronic Case Report Forms. It will be transcribed by the site from the source documents onto the electronic CRF. Data are reviewed and checked for omissions, apparent errors, and values requiring further clarifications using computerized and manual procedures. Data queries requiring clarification are communicated to the site for resolution. Only authorized personnel will make corrections to the clinical database and an audit trail will document all corrections.

10 Study Committees – Data Safety Monitoring Board

A Data Safety and Monitoring Board will be established, consisting of three experts in medical oncology, gastrointestinal cancer surgery and/or biostatistics.

The DSMB will receive regular information on safety results of the trial, namely a list of reported SAEs/SUSARs. Details on the work of the board will be described in a specific DSMB charter, to be jointly agreed upon by the board and the sponsor.

PART II – ETHICS AND GENERAL STUDY ADMINISTRATION

11 Ethical Aspects

11.1 Declaration of Helsinki / Good Clinical Practice

The Declaration of Helsinki is the accepted basis for clinical study ethics, and must be fully followed and respected by all those engaged in research on human beings. Any exceptions must be justified and stated in the protocol. The latest version of the Declaration of Helsinki is available at www.wma.net.

Additionally it is the responsibility of all those engaged in research on human beings to ensure that the study is performed in accordance with the international standards of Good Clinical Practice and according to all local laws and regulations concerning clinical studies.

11.2 Patient Information and Informed Consent

It is the responsibility of the investigator to obtain written informed consent from each patient participating in this study, after an adequate explanation of the aims, importance, anticipated benefits, potential hazards and consequences of the study according to applicable local laws. Written informed consent must be obtained before any study-specific procedures are performed. It must be also explained to the patient that they are completely free to refuse to enter the study or to withdraw from it at any time, for any reason, without incurring any penalty or withholding of treatment on the part of the investigator.

With the declaration of consent, the patient agrees to data about his/her disease being recorded within the context of the clinical trial and that it may be transferred to the sponsor in pseudonymized form.

The subject/patient also agrees to allow the monitor/auditor/health authorities to verify the patient data collected against the subject's/patient's original medical records for the purpose of source data verification.

The informed consent form personally signed and dated by the patient must be kept on file by the investigator(s) and documented in the CRF and the subject's medical records. The investigator must confirm with the sponsor that he/she has obtained written informed consent.

If new safety information results in significant changes to the risk/benefit assessment, the consent form should be reviewed and updated if necessary. All patients (including those already being treated) should be informed of the new information and must give their written informed consent to continue in the study.

If family doctors are to be informed of their patients' participation in the clinical study, this should be mentioned in the consent form.

11.3 Independent Ethics Committees and Regulatory Authorities

11.3.1 Approval of the Study by the Regulatory Authority and Independent Ethics Committees

It is the responsibility of the sponsor to obtain and maintain independent approval from the applicable regulatory authority and a positive opinion from the competent ethics committees to conduct the study in accordance with local legal requirements, statutes and the European Clinical Trial Directive.

Indemnity insurance will be arranged for the trial subjects in accordance with the applicable local law. The sponsor has provided insurance with HDI Gerling Industrie Versicherung AG, Niederlassung Düsseldorf, Am Schönenkamp 45, 40599 Düsseldorf; certificate No.:48 157572 03010

For Germany, the sponsor names the “Leiter der klinischen Prüfung” (LKP), who must be a physician with at least two years’ experience in the conduct of clinical trials of drugs according to § 4 (25) and § 40 (1) No. 5 of the AMG.

11.3.2 Notification of the Study

The sponsor is responsible for notifying the competent regional authority about the study and all principal investigators at the participating investigational sites, if applicable by local law.

11.3.3 Obligation to Report and Document

The sponsor and the investigator are responsible for complying with the requirements for reporting and documentation in accordance with local legal requirements, statutes and the European Clinical Trial Directive.

12 Conditions for Modifying the Protocol

Protocol modifications to ongoing studies must be made via amendment. The sponsor is responsible for obtaining independent approval for substantial amendments from the applicable regulatory authority and a positive opinion from the competent ethics committees in accordance with local legal requirements, statutes and the European Clinical Trial Directive. Approval must be obtained before any changes can be implemented, except for changes necessary in order to eliminate an immediate hazard to trial subjects or when the changes are non-substantial and involve only logistical or administrative aspects of the trial (e.g. change of telephone numbers).

13 Study Documentation, CRFs and Record-Keeping

13.1 Investigator's Files / Retention of Documents

The investigator must maintain adequate and accurate records to enable the conduct of the study to be fully documented and the study data to be subsequently verified. These documents should be classified into two separate categories: investigator's study file and subject/ patient data.

The investigator's study file will contain all essential documents such as the protocol/ amendments, patient information and informed consent form, ethics committee and regulatory authority approval, notification of the federal regulatory authority and competent regional authorities (if applicable), drug records, staff curriculum vitae and authorization forms, and other appropriate documents/correspondence, etc.

After completion of the study, the EDC vendor provides all data collected within eCRF including the audit trail to the investigators (data of the respective site) and the sponsor.

Patient data includes patient hospital/ clinic records (e.g. medical reports, surgery reports appointment book, medical records, pathology and laboratory reports, ECG, EEG, X-ray, etc.), signed informed consent forms and patient screening and eligibility screening forms.

The investigator must keep these two categories of documents on file for at least 15 years (or longer, as legally required) after completion or discontinuation of the study. The documents must be archived in a secure place and treated as confidential material.

Should the investigator wish to assign the study records to another party or move them to another location, the sponsor must be notified in advance.

If the investigator cannot guarantee this archiving requirement at the investigational site for any or all of the documents, special arrangements must be made between the investigator and the sponsor to store these in sealed containers outside of the site so that they can be returned sealed to the investigator in the event of a regulatory audit. Where source documents are required for the continued care of the patient, appropriate copies should be made for storing outside of the site.

All documents must be archived in a secure place and treated as confidential material.

13.2 Source Documents and Background Data

The investigator shall supply the sponsor on request with any required background data from the study documentation or clinic records. This is particularly important when case report forms are illegible or when errors in data transcription are suspected. In the event of special problems and/or governmental queries or requests for audit inspections, it is also necessary to have access to the complete study records, provided that patient confidentiality is protected.

According to the standards of the data protection law, all data obtained in the course of a clinical study must be treated with discretion in order to guarantee the rights of the patient's privacy.

13.3 Audits and Inspections

This study may be audited by the sponsor, any person authorized by the sponsor, or the competent health authority in order to determine the authenticity of the recorded data and compliance with the study protocol.

The investigator must be aware that source documents for this trial should be made available to appropriately qualified personnel working on behalf of the sponsor/monitor/auditor/health authority inspectors after appropriate notification for the purposes of source data verification and proper review of the study progress. The verification of the case report form data must be done via direct inspection of the source documents. The investigator agrees to comply with the sponsor and regulatory authority requirements regarding the auditing of the study.

All materials used in clinical studies are subjected to quality control.

13.4 Electronic Case Report Forms (eCRF)

A case report form will be completed for each study patient. It is the responsibility of the investigator to ensure the accuracy, completeness, legibility and timeliness of the data reported in the patient's eCRF which have been designed by the sponsor or his designee to record all observations and other pertinent data to the clinical investigation. This also applies to records for those patients who fail to complete the study. Source documentation supporting the eCRF data should indicate the patient's participation in the study and should document the dates and details of study procedures, adverse events and patient status. If a patient withdraws from the study, the reason must be noted in the eCRF. If a patient is withdrawn from the study because of a treatment-limiting adverse event, thorough efforts should be made to document the outcome clearly. The investigator or designated representative should complete the eCRF pages as soon as possible after information is collected, preferably on the same day that a study patient is seen for an examination, treatment, or any other study procedure. Any outstanding entries must be completed immediately after the final examination. All data entry and corrections are recorded in the audit trail.

Edit Checks will be programmed within the eCRF according to the Data Validation Plan (DVP) approved by the sponsor or his designee. There are Edit Checks which will arise during the data entry and other Edit Checks which will come up after completion of the eCRF. Edit Checks not programmed within the eCRF will be listed by using SAS.

14 Monitoring the Study

The monitor is responsible for familiarizing the investigator(s) and the entire center staff involved in the study with all study procedures, including the administration of the study drug.

The monitor will assist the investigator(s) in conducting the clinical study. The monitor must visit the clinical study center before the first patient has been enrolled (initiation visit), at least one routine monitoring visit during the course of the study if patients have been enrolled at the study center, and at study completion. The monitor is responsible for reviewing the ongoing study with the investigator(s) to verify adherence to the protocol and to deal with any problems that arise. The monitor must maintain the confidentiality of the study documents at all times. It is the responsibility of the study monitor to verify the study documents against the patient's original medical records.

The investigator (or his/her deputy) agrees to cooperate with the monitor to ensure that any problems detected in the course of these monitoring visits are resolved.

15 Confidentiality of Trial Documents and Patient Records

The investigator and the sponsor (or designated person) must ensure that all data obtained in the course of a clinical study is treated with discretion in order to guarantee the rights of the patient's privacy, according to the standards of the data protection law. CRFs or other documents should be submitted to the sponsor in pseudonymized form. The investigator should keep a patient identification log showing codes and names. The investigator should maintain documents not intended for submission to the sponsor, e.g. patients' written consent forms, in the strictest confidence.

16 Study Report and Publication Policy

This study will be entered into a clinical trial protocol registry and clinical results database. The sponsor is responsible for the timely reporting of study data. An integrated clinical study report (CSR) has to be completed one year after the end of the study (whether completed or prematurely terminated). The report has to be approved by the responsible specialist chosen by the sponsor, the project manager of the CRO, the statistician and the coordinating investigator/LKP by provision of their signatures. In this multi-center study, the main publication will be a full publication of all data from all sites. The following rules apply for the publication policies:

- Any publication of the results, either in part or in whole (abstracts in journals, oral presentations, etc.) by investigators or their representatives will require a pre-submission review by the sponsor and the coordinating investigator/LKP.
- For all publications the authors and the order of those will be a joint agreement between the coordinating investigator/LKP and the sponsor. The coordinating investigator will be given the choice to be the first or the last author for the main publication. The author positions (except for first and last author) will be based on recruitment, good data quality and scientific/infrastructural input to the study.
- For all publications including translational research data, the order of the authors will be determined according to the scientific input regarding the specific project and will be a joint agreement within the involved researchers, the sponsor and the coordinating investigator

17 Appendices

Appendix 1:	Adverse Event Categories for Determining Relationship to Test Drug
Appendix 2:	Definitions According to AMG and GCP-V, ICH Guidelines for Clinical Safety Data Management, Definitions and Standards for Expedited Reporting, Topic E2
Appendix 3:	ECOG Performance Status
Appendix 4:	RECIST 1.1
Appendix 5:	Cockroft-Gault Formula
Appendix 6:	NCI-CTCAE Version 4.03
Appendix 7:	QoL C30
Appendix 8:	DLQI
Appendix 9:	Skindex-16
Appendix 10:	FACT-EGFRI 18

Appendix 1 - Adverse Event Categories for Determining Relationship to Test Drug

(a) Related (must have one of them)

This category applies to those adverse events that are considered to be related to the test drug. An adverse event may be considered related if:

1. It follows a reasonable temporal sequence from administration of the drug
2. It cannot be reasonably explained by the known characteristics of the subject's clinical state, environmental or toxic factors or other modes of therapy administered to the subject
3. It disappears or decreases on cessation or reduction in dose. (There are important exceptions when an adverse event does not disappear upon discontinuation of the drug, yet drug-relatedness clearly exists: e.g. (1) bone marrow depression, (2) tardive dyskinesias.)
4. It follows a known pattern of response to the suspected drug
5. It reappears upon rechallenge

(b) Unrelated

This category is applicable to those adverse events which are judged to be clearly and incontrovertibly due only to extraneous causes (disease, environment, etc.) and do not meet the criteria for drug relationship listed under related.

Appendix 2 - Definitions According to AMG and GCP-V, ICH Guidelines for Clinical Safety Data Management, Definitions and Standards for Expedited Reporting, Topic E2

An adverse event is any untoward medical occurrence in a patient or clinical investigation subject who has been administered a pharmaceutical product, and which does not necessarily have to have a causal relationship with this treatment.

Adverse reactions are defined as all untoward and unintended responses to an investigational medicinal product related to any dose administered.

A serious adverse event or serious adverse reaction is any experience that suggests a significant hazard, contraindication, side effect or precaution. It is any adverse event that at any dose fulfills at least one of the following criteria:

- is fatal (results in death) *(NOTE: Death is an outcome, not an event)*
- is life-threatening *(NOTE: The term "life-threatening" refers to an event in which the patient was at immediate risk of death at the time of the event; it does not refer to an event which could hypothetically have caused a death had it been more severe)*
- required in-patient hospitalization or prolongation of existing hospitalization
- results in persistent or significant disability/incapacity
- is a congenital anomaly/birth defect
- is medically significant or requires intervention to prevent one or other of the outcomes listed above

Medical and scientific judgment should be exercised in deciding whether expedited reporting to the sponsor is appropriate in other situations, such as important medical events that may not be immediately life-threatening or result in death or hospitalization but may jeopardize the patient or may require intervention to prevent one of the outcomes listed in the definitions above. These situations should also usually be considered serious.

Examples of such events are intensive treatment in A&E or at home for allergic bronchospasm; blood dyscrasias or convulsions that do not result in hospitalization, or development of drug dependency or drug abuse.

An unexpected adverse event is one where the nature or severity is not consistent with the applicable product information.

Causality is initially assessed by the investigator. With respect to the obligation to report and document (regulatory authorities, ethics committees and other investigators) serious adverse events, causality can be one of two possibilities:

- No (unrelated; equals not drug-related)
- Yes (remotely, possibly or probably drug-related)

All adverse events not assessed as definitively “not drug-related” by the investigator will be considered as adverse drug reactions.

A suspected unexpected serious adverse reaction (SUSAR) is a serious adverse reaction whose nature or severity is not consistent with the applicable product information.

It is important that the severity of an adverse event is not confused with the seriousness of the event. For example, vomiting which persists for many hours may be severe, but is not necessarily a serious adverse event. On the other hand, stroke which results in only a limited degree of disability may be considered a mild stroke, but would be a serious adverse event.

A serious adverse event occurring during the study or which comes to the attention of the investigator within three weeks of stopping the treatment or during the protocol-defined follow-up period, if this is longer, must be reported, whether considered treatment-related or not. In addition, serious adverse events occurring after this time should be reported if considered related to test “drug”.

Such preliminary reports will be followed by detailed descriptions later that will include copies of hospital case reports, autopsy reports and other documents.

For serious adverse events, the following must be assessed and recorded on the adverse events page of the case report form: intensity, relationship to test substance, action taken, and outcome to date.

The obligation to document and report must be adhered to according to the national and international laws and regulations.

For contact details and fax no. for SAE and pregnancy reporting, please refer to page 10.

Appendix 3 - ECOG Performance Status

GRADE	SCALE
0	Fully active, able to carry out all pre-disease performance without restriction
1	Restricted in physically strenuous activity but ambulatory and able to carry out work of a light or sedentary nature, e.g., light housework, office work
2	Ambulatory and capable of all self-care, but unable to carry out work activities. Up and about more than 50% of waking hours.
3	Capable of only limited self-care, confined to bed or chair more than 50% of waking hours
4	Completely disabled. Cannot carry out any self-care. Totally confined to bed or chair.
5	Dead

Appendix 4 - RECIST 1.1

According to:

New response evaluation criteria in solid tumours:
revised RECIST guideline (version 1.1)

Eisenhauer EA, Therasse P, Bogaerts J et al., European Journal of Cancer 45 (2009): 228–247[48]

RECISTGuidelines 1.1.pdf

Appendix 5 - Cockroft-Gault Formula

$$\text{Calculated } CL_{CR} \text{ (ml/min)} = \frac{[(140 - \text{subject's age in years}) \times \text{subject's actual body weight in kilograms}]\ ^{*}}{72 \times \text{subject's serum creatinine (in mg/dL)}}$$

*: x 0.85 for females

$$\text{Calculated } CL_{CR} \text{ (ml/min)} = \frac{[(140 - \text{subject's age in years}) \times \text{subject's actual body weight in kilograms}] \times K^{*}}{\text{subject's serum creatinine (in µmol/L)}}$$

K*: 1.23 for males, 1.05 for females

Appendix 6 - NCI-CTCAE version 4.03

NCI-CTC Version 4.03

Appendix 7 - QoL C30

Appendix 8 - DLQI

Appendix 9 - Skindex 16

Appendix 10 - FACT-EGFRI 18

18 References

1. Ferlay J, Autier P, Boniol M, et al: Estimates of the cancer incidence and mortality in Europe in 2006. Ann Oncol 18:581-92, 2007

2. Robert-Koch-Institut: Krebs in Deutschland 2007/2008, 2012

3. Siegel R, Naishadham D, Jemal A: Cancer statistics, 2012. CA Cancer J Clin 62:10-29, 2012

4. Howlader N NA, Krapcho M, Neyman N, Aminou R, Altekruse SF, Kosary CL, Ruhl J, Tatalovich Z, Cho H, Mariotto A, Eisner MP, Lewis DR, Chen HS, Feuer EJ, Cronin KA (eds): SEER Cancer Statistics Review, 1975-2009 (Vintage 2009 Populations), in National Cancer Institute. Bethesda M (ed), 2011

5. Schmiegel W, Reinacher-Schick A, Arnold D, et al: [Update S3-guideline "colorectal cancer" 2008]. Z Gastroenterol 46:799-840, 2008

6. Tournigand C, Andre T, Achille E, et al: FOLFIRI followed by FOLFOX6 or the reverse sequence in advanced colorectal cancer: a randomized GERCOR study. J Clin Oncol 22:229-37, 2004

7. Yarom N, Jonker DJ: The role of the epidermal growth factor receptor in the mechanism and treatment of colorectal cancer. Discov Med 11:95-105, 2011

8. Yokota T: Are KRAS/BRAF mutations potent prognostic and/or predictive biomarkers in colorectal cancer. Anti-Cancer Agents in Medicinal Chemistry:163-171, 2012

9. Bokemeyer C, Bondarenko I, Makhson A, et al: Fluorouracil, leucovorin, and oxaliplatin with and without cetuximab in the first-line treatment of metastatic colorectal cancer. J Clin Oncol 27:663-71, 2009

10. Douillard JY, Siena S, Cassidy J, et al: Randomized, phase III trial of panitumumab with infusional fluorouracil, leucovorin, and oxaliplatin (FOLFOX4) versus FOLFOX4 alone as first-line treatment in patients with previously untreated metastatic colorectal cancer: the PRIME study. J Clin Oncol 28:4697-705, 2010

11. Peeters M, Price TJ, Cervantes A, et al: Randomized phase III study of panitumumab with fluorouracil, leucovorin, and irinotecan (FOLFIRI) compared with FOLFIRI alone as second-line treatment in patients with metastatic colorectal cancer. J Clin Oncol 28:4706-13, 2010

12. Van Cutsem E, Kohne CH, Hitre E, et al: Cetuximab and chemotherapy as initial treatment for metastatic colorectal cancer. N Engl J Med 360:1408-17, 2009

13. Van Cutsem E, Peeters M, Siena S, et al: Open-label phase III trial of panitumumab plus best supportive care compared with best supportive care alone in patients with chemotherapy-refractory metastatic colorectal cancer. J Clin Oncol 25:1658-64, 2007

14. Amado RG, Wolf M, Peeters M, et al: Wild-type KRAS is required for panitumumab efficacy in patients with metastatic colorectal cancer. J Clin Oncol 26:1626-34, 2008

15. Lievre A, Bachet JB, Boige V, et al: KRAS mutations as an independent prognostic factor in patients with advanced colorectal cancer treated with cetuximab. J Clin Oncol 26:374-9, 2008

16. Van Cutsem E, Kohne CH, Lang I, et al: Cetuximab plus irinotecan, fluorouracil, and leucovorin as first-line treatment for metastatic colorectal cancer: updated analysis of overall survival according to tumor KRAS and BRAF mutation status. J Clin Oncol 29:2011-9, 2011

17. Jimeno A, Messersmith WA, Hirsch FR, et al: KRAS mutations and sensitivity to epidermal growth factor receptor inhibitors in colorectal cancer: practical application of patient selection. J Clin Oncol 27:1130-6, 2009

18. Wong R, Cunningham D: Using predictive biomarkers to select patients with advanced colorectal cancer for treatment with epidermal growth factor receptor antibodies. J Clin Oncol 26:5668-70, 2008

19. De Roock W, Claes B, Bernasconi D, et al: Effects of KRAS, BRAF, NRAS, and PIK3CA mutations on the efficacy of cetuximab plus chemotherapy in chemotherapy-refractory metastatic colorectal cancer: a retrospective consortium analysis. Lancet Oncol 11:753-62, 2010

20. Di Nicolantonio F, Martini M, Molinari F, et al: Wild-type BRAF is required for response to panitumumab or cetuximab in metastatic colorectal cancer. J Clin Oncol 26:5705-12, 2008

21. Loupakis F, Pollina L, Stasi I, et al: PTEN expression and KRAS mutations on primary tumors and metastases in the prediction of benefit from cetuximab plus irinotecan for patients with metastatic colorectal cancer. J Clin Oncol 27:2622-9, 2009

22. Peeters M, Oliner KS, Parker A, et al: Massively parallel tumor multigene sequencing to evaluate response to panitumumab in a randomized phase III study of metastatic colorectal cancer. Clin Cancer Res 19: 1902 - 12, 2013

23. Oliner KS, Douillard J-Y, Siena S; et al: Analysis of *KRAS//NRAS* and *BRAF* mutations in the phase III PRIME study of panitumumab (pmab) plus FOLFOX as first-line treatment (tx) for metastatic colorectal cancer (mCRC). 2013 Annual ASCO Meeting. Abstract 3511

24. Chibaudel B, Maindrault-Goebel F, Lledo G, et al: Can chemotherapy be discontinued in unresectable metastatic colorectal cancer? The GERCOR OPTIMOX2 Study. J Clin Oncol 27:5727-33, 2009

25. Adams RA, Meade AM, Seymour MT, et al: Intermittent versus continuous oxaliplatin and fluoropyrimidine combination chemotherapy for first-line treatment of advanced colorectal cancer: results of the randomised phase 3 MRC COIN trial. Lancet Oncol 12:642-53, 2011

26. Tournigand C, Cervantes A, Figer A, et al: OPTIMOX1: a randomized study of FOLFOX4 or FOLFOX7 with oxaliplatin in a stop-and-Go fashion in advanced colorectal cancer--a GERCOR study. J Clin Oncol 24:394-400, 2006

27. Tournigand C, Samson B, Scheithauer W, et al: Bevacizumab (Bev) with or without erlotinib as maintenance therapy, following induction first-line chemotherapy plus Bev, in patients (pts) with metastatic colorectal cancer (mCRC): Efficacy and safety results of the International GERCOR DREAM phase III trial. J Clin Oncol 30:LBA3500, 2012

28. Potthoff K, Hofheinz R, Hassel JC, et al: Interdisciplinary management of EGFR-inhibitor-induced skin reactions: a German expert opinion. Ann Oncol 22:524-35, 2011

29. Lacouture ME, Anadkat MJ, Bensadoun RJ, et al: Clinical practice guidelines for the prevention and treatment of EGFR inhibitor-associated dermatologic toxicities. Support Care Cancer 19:1079-95, 2011

30. Loprinzi CL: Minocycline for preventing EGFR inhibitor-induced rash. Curr Oncol Rep 10:303, 2008

31. Bernier J, Bonner J, Vermorken JB, et al: Consensus guidelines for the management of radiation dermatitis and coexisting acne-like rash in patients receiving radiotherapy plus EGFR inhibitors for the treatment of squamous cell carcinoma of the head and neck. Ann Oncol 19:142-9, 2008

32. Perez-Soler R, Delord JP, Halpern A, et al: HER1/EGFR inhibitor-associated rash: future directions for management and investigation outcomes from the HER1/EGFR inhibitor rash management forum. Oncologist 10:345-56, 2005

33. Rhee J, Oishi K, Garey J, et al: Management of rash and other toxicities in patients treated with epidermal growth factor receptor-targeted agents. Clin Colorectal Cancer 5 Suppl 2:S101-6, 2005

34. Segaert S, Van Cutsem E: Clinical signs, pathophysiology and management of skin toxicity during therapy with epidermal growth factor receptor inhibitors. Ann Oncol 16:1425-33, 2005

35. Sipples R: Common side effects of anti-EGFR therapy: acneform rash. Semin Oncol Nurs 22:28-34, 2006

36. Pizzo B: New directions in oncology nursing care: focus on gefitinib in patients with lung cancer. Clin J Oncol Nurs 8:385-92, 2004

37. Jatoi A, Thrower A, Sloan JA, et al: Does sunscreen prevent epidermal growth factor receptor (EGFR) inhibitor-induced rash? Results of a placebo-controlled trial from the North Central Cancer Treatment Group (N05C4). Oncologist 15:1016-22, 2010

38. Lacouture ME, Mitchell EP, Piperdi B, et al: Skin toxicity evaluation protocol with panitumumab (STEPP), a phase II, open-label, randomized trial evaluating the impact of a pre-Emptive Skin treatment regimen on skin toxicities and quality of life in patients with metastatic colorectal cancer. J Clin Oncol 28:1351-7, 2010

39. Segaert S, Van Cutsem E: Clinical management of EGFRI dermatologic toxicities: the European perspective. Oncology (Williston Park) 21:22-6, 2007

40. Eilers RE, Jr., Gandhi M, Patel JD, et al: Dermatologic infections in cancer patients treated with epidermal growth factor receptor inhibitor therapy. J Natl Cancer Inst 102:47-53, 2010

41. Monroe EW: Relative efficacy and safety of loratadine, hydroxyzine, and placebo in chronic idiopathic urticaria and atopic dermatitis. Clin Ther 14:17-21, 1992

42. Monroe EW, Bernstein DI, Fox RW, et al: Relative efficacy and safety of loratadine, hydroxyzine, and placebo in chronic idiopathic urticaria. Arzneimittelforschung 42:1119-21, 1992

43. Ocvirk J, Cencelj S: Management of cutaneous side-effects of cetuximab therapy in patients with metastatic colorectal cancer. J Eur Acad Dermatol Venereol 24:453-9, 2010

44. Shohrati M, Davoudi SM, Keshavarz S, et al: Cetirizine, doxepine, and hydroxyzine in the treatment of pruritus due to sulfur mustard: a randomized clinical trial. Cutan Ocul Toxicol 26:249-55, 2007

45. Gutzmer R, Werfel T, Kapp A, et al: [Cutaneous side effects of EGF-receptor inhibition and their management]. Hautarzt 57:509-13, 2006

46. Burtness B, Anadkat M, Basti S, et al: NCCN Task Force Report: Management of dermatologic and other toxicities associated with EGFR inhibition in patients with cancer. J Natl Compr Canc Netw 7 Suppl 1:S5-21; quiz S22-4, 2009

47. Hu JC, Sadeghi P, Pinter-Brown LC, et al: Cutaneous side effects of epidermal growth factor receptor inhibitors: clinical presentation, pathogenesis, and management. J Am Acad Dermatol 56:317-26, 2007

48. Eisenhauer EA, Therasse P, Bogaerts J, et al: New response evaluation criteria in solid tumours: revised RECIST guideline (version 1.1). Eur J Cancer 45:228-47, 2009
