# Supplementary material for: Panitumumab plus 5-fluorouracil and folinic acid or 5-fluorouracil and folinic acid alone as maintenance therapy in RAS wild-type metastatic colorectal cancer (PanaMa, AIO KRK 0212): final efficacy analysis of a randomised, open-label, phase 2 trial
Source: eClinicalMedicine. 2024 Dec 16;79:103004. doi: 10.1016/j.eclinm.2024.103004 (PMC11719858; doi:10.1016/j.eclinm.2024.103004)
Supplement: Supplementary Data [file mmc2.docx]

**Supplementary Data**

**Panitumumab plus 5-fluorouracil and folinic acid or 5-fluorouracil and folinic acid alone as maintenance therapy in RAS wild-type metastatic colorectal cancer (PanaMa, AIO KRK 0212): final efficacy analysis of a randomised, open-label, phase 2 trial.**

Brief title: FU/FA +/- panitumumab maintenance: final results

**Arndt Stahler,**^a^ Meinolf Karthaus,^b^ Stefan Fruehauf,^c^ Ullrich Graeven,^d^ Lothar Müller,^e^ Ludwig Fischer von Weikersthal,^f^ Karel Caca,^g^ Eray Goekkurt,^h,i^ Alexej Ballhausen,^a^ Greta Sommerhäuser,^a^ Annabel H. S. Alig,^a^ Swantje Held,^j^ Armin Jarosch,^k^ David Horst,^k,l^ Anke Reinacher-Schick,^m^ Stefan Kasper,^l,n^ Volker Heinemann,^l,o^ Sebastian Stintzing,^a,l^ Tanja Trarbach,^n,p^ Dominik P. Modest,^a,l^

^a^Department of Hematology, Oncology and Tumorimmunology, Charité-Universitätsmedizin Berlin, Freie Universität Berlin, Humboldt-Universität zu Berlin, and Berlin Institute of Health, Berlin, Germany.

^b^Department of Hematology and Oncology, Munich Hospital Neuperlach, Munich, Germany.

^c^Dr Hancken Hospital, Stade, Germany.

^d^Kliniken Maria Hilf GmbH, Mönchengladbach, Germany.

^e^Oncology Practice UnterEms, Leer, Germany.

^f^Gesundheitszentrum St Marien, Amberg, Germany.

^g^Department of Gastroenterology, Hematology and Oncology, Hospital Ludwigsburg, Ludwigsburg, Germany.

^h^Practice of Hematology and Oncology (HOPE), Hamburg, Germany.

^i^University Cancer Center Hamburg (UCCH), Hamburg, Germany.

^j^ClinAssess GmbH, Leverkusen, Germany.

^k^Charité Universitätsmedizin, Freie Universität Berlin, Humboldt-Universität zu Berlin, and Berlin Institute of Health, Institute of Pathology, Berlin, Germany.

^l^German Cancer Consortium (DKTK), German Cancer Research Centre (DKFZ), Heidelberg, Germany.

^m^St Joseph Hospital, Bochum, Germany.

^n^Department of Medical Oncology, West German Cancer Center, Westdeutsches Tumorzentrum, University Hospital of Essen, Essen, Germany.

^o^Department of Medicine 2I and Comprehensive Cancer Center, University Hospital (LMU), Munich, Germany.

^p^Reha-Zentrum am Meer, Bad Zwischenahn, Niedersachsen, Germany


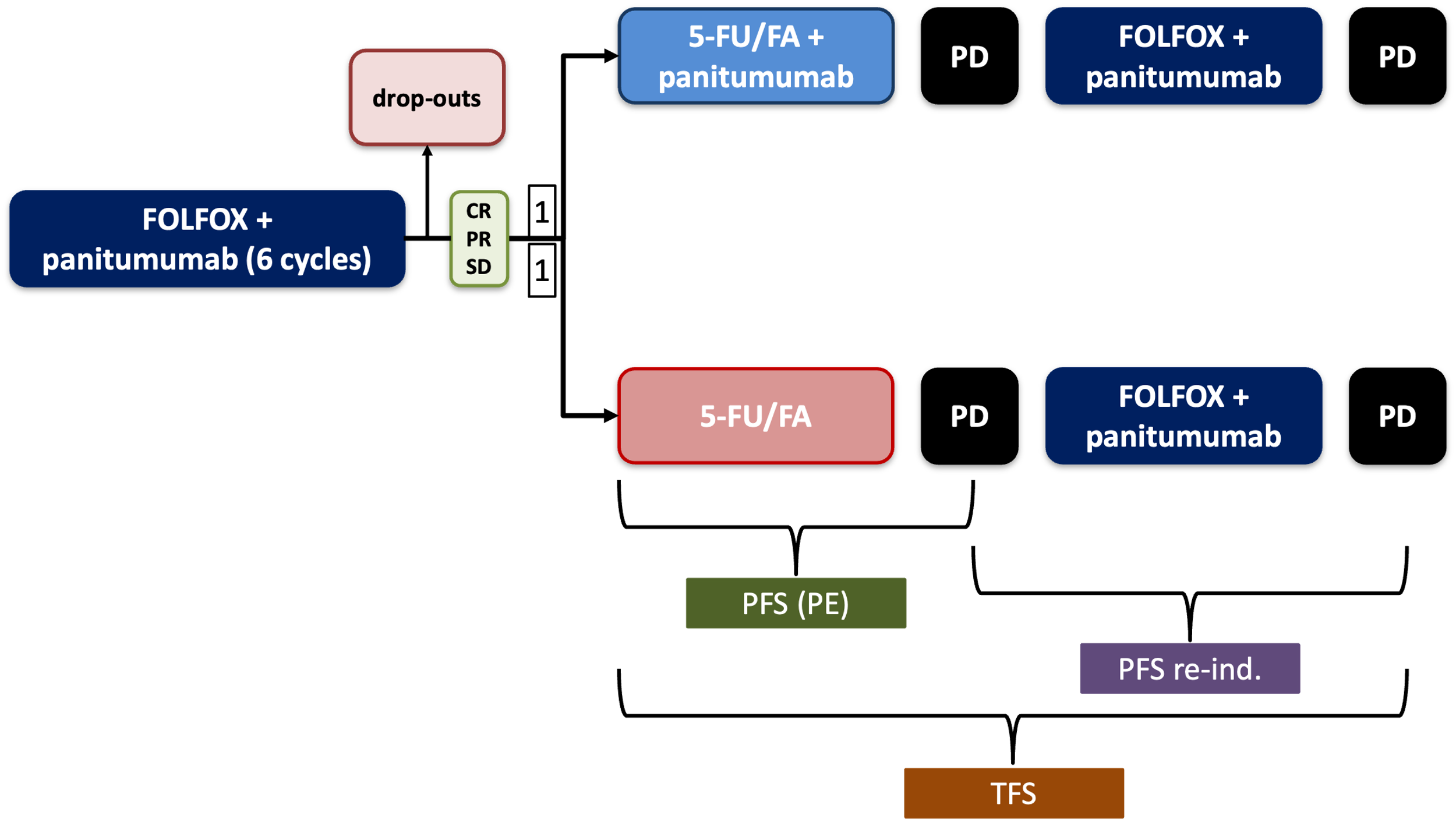


**Supplementary Figure 1:** Study design and endpoints of the randomized phase 2 open-label PanaMa trial. FU/FA: 5-fluorouracil and folinic acid; FOLFOX: 5-fluorouracil, folinic acid and oxaliplatin; CR: complete remission; PR: partial remission; SD: stable disease; PD: progressive disease, PFS: progression-free survival; PE: primary endpoint; PFS re-ind.: progression-free survival of re-induction; TFS: time to failure of strategy.

Definitions: PFS (PE): time from randomization to first disease progression according to RECIST 1.1 criteria (locally assessed) or death from any cause; PFS re-ind.: time length between the date of objective disease progression during maintenance and the date of first disease progression (according to RECIST criteria 1.1) or death after start of re-induction (whichever occurs first); TFS: defined as the time from randomisation to second objective disease progression, or death from any cause, whichever first, patients without re-induction therapy were censored after maintenance therapy; overall survival (OS, not displayed): time from randomization to death from any cause.

| **Supplementary Table 1:** Baseline characteristics of randomized patients receiving re-induction treatment in the PanaMa trial. | | | |
| --- | --- | --- | --- |
| **Variable** | **Overall**  **(*N*=128)** | **FU/FA plus pmab**  **(*N*=50)** | **FU/FA**  **(*N*=78)** |
| **Sex, *N* (%)**  Female  Male | 40 (31·3)  88 (68·8) | 14 (28·0)  36 (72·0) | 26 (33·3)  52 (66·7) |
| **Age**  Median in years (range) | 65 (30-84) | 65 (44-84) | 65 (30-82) |
| **ECOG, *N* (%)**  0  1 | 75 (58·6)  53 (41·4) | 27 (54·0)  23 (46·0) | 48 (61·5)  30 (38·5) |
| **Ethnicity, *N* (%)**  Caucasian  Asian | 127 (99·2)  1 (0·8) | 50 (100·0)  0 (0·0) | 77 (98·7)  1 (1·3) |
| **Body mass index**  Median (range) | 24·9 (16·5-43·3) | 24·8 (17·3-33·9) | 24·9 (16·5-43·3) |
| **Baseline CEA level**  Median (ng/ml) | 47·3 | 44·0 | 50·4 |
| **Previous resection of primary tumor, *N* (%)**  Yes | 84 (65·6) | 33 (66·0) | 51 (65·4) |
| **Previous radiation, *N* (%)**  **Yes** | 19 (14·8) | 7 (14·0) | 12 (15·4) |
| **Prior adjuvant therapy, *N* (%)**  All therapies  Oxaliplatin-based | 14 (10·9)  4 (3·1) | 3 (6·0)  2 (4·0) | 11 (14·1)  2 (2·6) |
| **One prior cycle of FOLFOX, *N* (%)**  Given | 19 (14·8) | 4 (8·0) | 15 (19·2) |
| **Primary tumor location, *N* (%)**  Left-sided  Right-sided  Both | 102 (79·7)  21 (16·4)  5 (3·9) | 37 (74·0)  11 (22·0)  2 (4·0) | 65 (83·3)  10 (12·8)  3 (3·8) |
| **Metastatic sites, *N* (%)**  Liver  Liver-limited  Lung  Lymph nodes  Peritoneum  Other | 109 (85·2)  47 (36·7)  38 (29·7)  43 (33·6)  22 (17·2)  19 (14·8) | 42 (84·0)  17 (34·0)  14 (28·0)  23 (46·0)  5 (10·0)  6 (12·0) | 67 (85·9)  30 (38·5)  24 (30·8)  20 (25·6)  17 (21·8)  13 (16·7) |
| **No· of organs involved, *N* (%)**  1  >1 | 58 (45·3)  70 (54·7) | 21 (42·0)  29 (58·0) | 37 (47·4)  41 (52·6) |
| **Onset of metastatic disease, *N* (%)**  Synchronous  Metachronous | 104 (81·3)  24 (18·8) | 44 (88·0)  6 (12·0) | 60 (76·9)  18 (23·1) |
| Legend: FU/FA = 5-fluorouracil and folinic acid, ECOG = Eastern Cooperative Oncology Group, CEA = carcinoembryonic antigen, FOLFOX = 5-fluorouracil, folinic acid, oxaliplatin, no. = number. | | | |

| **Supplementary Table 2:** Baseline characteristics of randomized patients who did not receive re-induction treatment in the PanaMa trial. | | | |
| --- | --- | --- | --- |
| **Variable** | **Overall**  **(*N*=120)** | **FU/FA plus pmab**  **(*N*=75)** | **FU/FA**  **(*N*=45)** |
| **Sex, *N* (%)**  Female  Male | 43 (35·8)  77 (64·2) | 24 (32·0)  51 (68·0) | 19 (42·2)  26 (57·8) |
| **Age**  Median in years (range) | 66 (45-86) | 68 (45-83) | 65 (48-86) |
| **ECOG, *N* (%)**  0  1 | 72 (60·0)  48 (40·0) | 43 (57·3)  32 (42·7) | 29 (64·4)  16 (35·6) |
| **Ethnicity, *N* (%)**  Caucasian | 120 (100·0) | 75 (100·0) | 45 (100·0) |
| **Body mass index**  Median (range) | 25·9 (17·6-46·8) | 26·0 (19·2-46·8) | 25·7 (17·6-40·1) |
| **Previous resection of primary tumor, *N* (%)**  Yes | 92 (76·7) | 61 (81·3) | 31 (68·9) |
| **Previous radiation, *N* (%)**  **Yes** | 9 (7·5) | 7 (9·3) | 2 (4·4) |
| **Prior adjuvant therapy, *N* (%)**  All therapies  Oxaliplatin-based | 12 (10·0)  8 (6·7) | 9 (12·0)  6 (8·0) | 3 (6·7)  2 (4·4) |
| **One prior cycle of FOLFOX, *N* (%)**  Given | 10 (8·3) | 8 (10·7) | 2 (4·4) |
| **Primary tumor location, *N* (%)**  Left-sided  Right-sided  Both  Unknown | 97 (80·8)  17 (14·2)  5 (4·2)  1 (0.8) | 62 (82·7)  8 (10·7)  4 (5·3)  1 (1.3) | 35 (77·8)  9 (20·0)  1 (2·2  0 (0.0)) |
| **Metastatic sites, *N* (%)**  Liver  Liver-limited  Lung  Lymph nodes  Peritoneum  Other | 96 (80·0)  55 (45·8)  24 (20·0)  37 (30·8)  16 (13·3)  13 (10·8) | 58 (77·3)  36 (48·0)  14 (18·7)  22 (29·3)  8 (10·7)  4 (5·3) | 38 (84·4)  19 (42·2)  10 (22·2)  15 (33·3)  8 (17·8)  9 (20·0) |
| **No·of organs involved, *N* (%)**  1  >1 | 74 (61·7)  46 (38·3) | 49 (65·3)  26 (34·7) | 25 (55·6)  20 (44·4) |
| **Onset of metastatic disease, *N* (%)**  Synchronous  Metachronous | 96 (80·0)  24 (20·0) | 57 (76·0)  18 (24·0) | 39 (86·7)  6 (13·3) |
| **Subsequent Treatment, *N* (%)**  Documented subsequent treatment  Treatment lines, median (range)  Radiation (incl. RITA, SIRT)  Surgery incl. ablation | 80 (66·6)  2 (1-8)  19 (15·8)  16 (13·3) | 53 (70·7)  2 (1-7)  10 (13·3)  8 (10·7) | 27 (60·0)  2 (1-8)  9 (20·0)  8 (17·8) |
| **Justification of the investigator not to perform re-induction*, *N* (%)**  No justification given by investigator  Toxicity / deterioration of general condition  Secondary intervention planned  No evidence of disease  Disease progression during maintenance  Patient’s wish, N.O.S.  Investigator’s decision, N.O.S.  Regulatory aspects  Delayed detection of second malignancy  Car accident  Non-compliance | 54 (45·0)  20 (16·7)  29 (24·2)  4 (3·3)  2 (1·7)  2 (1·7)  3 (2·5)  2 (1·7)  2 (1·7)  1 (0·8)  1 (0·8) | 32 (42·7)  15 (20·0)  17 (22·7)  3 (4·0)  1 (1·3)  1 (1·3)  2 (2·7)  2 (2·7)  1 (1·3)  0 (0·0)  1 (1·3) | 22 (48·9)  5 (11·1)  12 (26·7)  1 (2·2)  1 (2·2)  1 (2·2)  1 (2·2)  0 (0·0)  1 (2·2)  1 (2·2)  0 (0·0) |
| Legend: FU/FA = 5-fluorouracil and folinic acid, ECOG = Eastern Cooperative Oncology Group, CEA = carcinoembryonic antigen, FOLFOX = 5-fluorouracil, folinic acid, oxaliplatin, no. = number, RITA = radiofrequency interstitial thermal ablation; SIRT = selective interarterial radiotherapy, incl. = inclusive, N.O.S. = not otherwisely specified; *free text response declared exclusively by the investigator | | | |


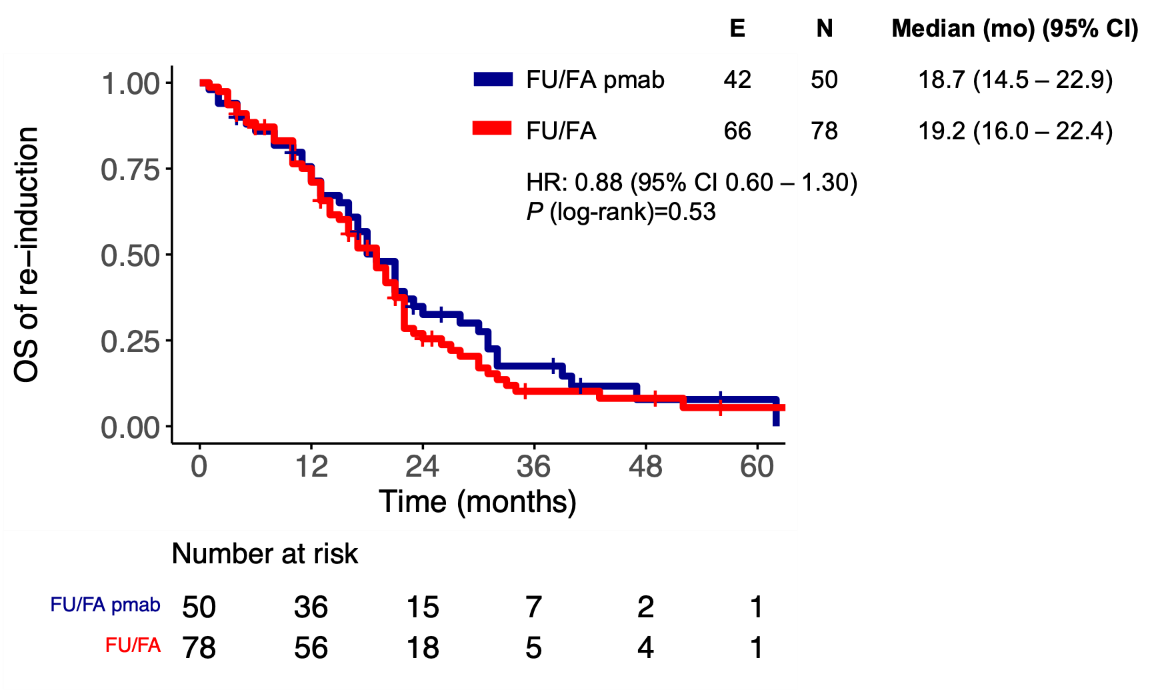


**Supplementary Figure 2:** Overall survival (OS) of re-induction treatment in the PanaMa trial. FU/FA = 5-fluorouracil and folinic acid, pmab = panitumumab, FOLFOX = 5-fluorouracil, folinic acid, oxaliplatin, mo = months, HR = hazard ratio, CI = confidence interval.

| **Supplementary Table 3:** Multivariate Cox regression analyses of prognostically relevant clinical and molecular subgroups in the PanaMa trial. | | | | |
| --- | --- | --- | --- | --- |
| **Variable** | **PFS** | | **OS** | |
|  | **HR**  **(95% CI)** | ***P*** | **HR**  **(95% CI)** | ***P*** |
| **Clinical** | | | | |
| Age | 0.88  (0.67 – 1.15) | 0.33 | 0.62  (0.47 – 0.84) | **0.002** |
| Sex | 1.01  (0.76 – 1.34) | 0.96 | 1.16  (0.86 – 1.58) | 0.34 |
| ECOG | 1.08  (0.82 – 1.43) | 0.59 | 1.25  (0.92 – 1.68) | 0.15 |
| Liver-limited disease | 0.91  (0.63 – 1.31) | 0.60 | 1.11  (0.74 – 1.65) | 0.63 |
| No of organs (1 vs. >1) | 1.23  (1.01 – 1.50) | **0.04** | 1.29  (1.05 – 1.60) | **0.02** |
| Syn- vs. Metachronous | 1.16  (0.75 – 1.81) | 0.50 | 1.20  (0.75 – 1.93) | 0.45 |
| Primary tumor side | 0.92  (0.78 – 1.09) | 0.32 | 1.13  (0.97 – 1.32) | 0.11 |
| Adj. therapy | 1.05  (0.60 – 1.82) | 0.87 | 0.86  (0.46 – 1.61) | 0.64 |
| Surgery of primary tumor | 0.79  (0.58 – 1.07) | 0.12 | 0.49  (0.35 – 0.68) | **<0.001** |
| **Molecular** | | | | |
| Hyperselection | 1.29  (0.89 – 1.86) | 0.18 | 1.83  (1.25 – 2.68) | **0.002** |
| CMS | 1.02  (0.75 – 1.39) | 0.91 | 0.89  (0.63 – 1.24) | 0.47 |
| Legend: PFS = progression-free survival; OS = overall survival; HR = hazard ratio; CI = confidence interval; no. = number, adj. = adjuvant, CMS = consensus molecular subtypes. *P* values considered significant are displayed **bold.** | | | | |

| **Supplementary Table 4:** Subgroup analysis of prognostic impact of combined hyperselection status (WT vs. MUT) and primary tumor sidedness (left vs. right) on PFS, PFS re-ind., TFS and OS | | | | | | | | | | | | |
| --- | --- | --- | --- | --- | --- | --- | --- | --- | --- | --- | --- | --- |
| Subgroup | **PFS** | | | **PFS re-ind.** | | | **TFS** | | | **OS** | | |
|  | **Months**  **(median, 95% CI)** | **HR**  **(95% CI)** | ***P***  **(interact.)** | **Months**  **(median, 95% CI)** | **HR**  **(95% CI)** | ***P***  **(interact.)** | **Months**  **(median, 95% CI)** | **HR**  **(95% CI)** | ***P***  **(interact.)** | **Months**  **(median, 95% CI)** | **HR**  **(95% CI)** | ***P***  **(interact.)** |
| WT – left  *N*=141 | 7.5  (6.0 – 9.0) | 0.89  (0.52 – 1.53) | 0.31 | 5.9  (4.0 – 7.7) | 0.37  (0.17 – 0.81) | **0.05** | 16.6  (14.3 – 18.9) | 0.81  (0.37 – 1.78) | 0.56 | 29.9  (25.0 – 34.8) | 0.42  (0.24 – 0.73) | **0.005** |
| MUT – left  *N*=21 | 5.0  (2.8 – 7.2) | 1.36  (0.70 – 2.64) |  | 4.1  (3.9 – 4.3) | 0.51  (0.19 – 1.36) |  | 12.9  (6.3 – 19.5) | 1.16  (0.43 – 3.13) |  | 23.8  (19.1 – 28.4) | 0.72  (0.37 – 1.43) |  |
| WT – right  *N*=15 | 8.0  (3.8 – 12.3) | 0.79  (0.38 – 1.64) |  | 6.3  (2.6 – 10.1) | 0.60  (0.23 – 1.56) |  | 13.9  (8.9 – 19.0) | 1.18  (0.45 – 3.07) |  | 26.0  (18.6 – 33.4) | 0.44  (0.21 – 0.93) |  |
| MUT – right  *N*=16 | 5.3  (1.8 – 8.7) | Reference |  | 2.0  (1.7 – 2.4) | Reference |  | 16.4  (6.7 – 26.1) | Reference |  | 13.5  (2.1 – 24.9) | Reference |  |
| Legend: PFS = progression-free survival; PFS re-ind. = progression-free survival of re-induction; TFS = time to failure of strategy; OS = overall survival; CI = confidence interval; HR = hazard ratio; interact.: interaction; WT = hyperselection wild-type; MUT = hyperselection mutated. *P* values considered significant are displayed **bold.** | | | | | | | | | | | | |

| **Supplementary Table 5:** Subgroup analysis of predictive impact of combined hyperselection status (WT vs. MUT) and primary tumor sidedness (left vs. right) on treatment arm efficacy with regard to PFS, PFS re-ind., TFS and OS | | | | | | | | |
| --- | --- | --- | --- | --- | --- | --- | --- | --- |
| **Endpoints** | **WT – left**  ***N*=141**  ***N*(re-ind.)=75** | | **MUT – left**  ***N*=21**  ***N*(re-ind.)=9** | | **WT – right**  ***N*=15**  ***N*(re-ind.)=11** | | **MUT – right**  ***N*=16**  ***N*(re-ind.)=7** | |
|  | **FU/FA+pmab**  ***N*=71**  ***N*(re-ind.)=27** | **FU/FA**  ***N*=70**  ***N*(re-ind.)=48** | **FU/FA+pmab**  ***N*=8**  ***N*(re-ind.)=4** | **FU/FA**  ***N*=13**  ***N*(re-ind.)=5** | **FU/FA+pmab**  ***N*=10**  ***N*(re-ind.)=6** | **FU/FA**  ***N*=5**  ***N*(re-ind.)=5** | **FU/FA+pmab**  ***N*=6**  ***N*(re-ind.)=3** | **FU/FA**  ***N*=10**  ***N*(re-ind.)=4** |
| PFS |  | | | | | | | |
| Months | 8.8 | 6.0 | 3.6 | 5.0 | 9.2 | 5.8 | 5.3 | 3.7 |
| HR  (95% CI) | 0.72  (0.51 – 1.02) | | 1.14  (0.45 – 2.87) | | 0.61  (0.19 – 1.93) | | 0.99  (0.35 – 2.79) | |
| *P* | 0.06 | | 0.79 | | 0.40 | | 0.98 | |
| PFS re-ind |  | | | | | | | |
| Months | 4.2 | 7.4 | 2.1 | 7.4 | 5.1 | 6.3 | 4.8 | 1.9 |
| HR  (95% CI) | 2.34  (1.41 – 3.88) | | 1.04  (0.24 – 4.44) | | 0.62  (0.16 – 2.32) | | 0.16  (0.02 – 1.48) | |
| *P* | **0.001** | | 0.95 | | 0.48 | | 0.11 | |
| TFS |  | | | | | | | |
| Months | 20.0 | 16.4 | 22.2 | 12.9 | 17.1 | 13.8 | 16.4 | 9.2 |
| HR  (95% CI) | 0.96  (0.59 – 1.56) | | 1.09  (0.26 – 4.59) | | 0.75  (0.21 – 2.70) | | 0.74  (0.13 – 4.12) | |
| *P* | 0.87 | | 0.91 | | 0.66 | | 0.73 | |
| OS |  | | | | | | | |
| Months | 37.0 | 26.9 | 15.1 | 26.1 | 26.0 | 21.6 | 10.3 | 13.5 |
| HR  (95% CI) | 0.90  (0.61 – 1.33) | | 1.84  (0.72 – 4.72) | | 0.43  (0.12 – 1.50) | | 0.97  (0.34 – 2.75) | |
| *P* | 0.58 | | 0.21 | | 0.18 | | 0.95 | |
| Legend: FU/FA = 5-fluorouracil and folinic acid; pmab = panitumumab; PFS = progression-free survival; PFS re-ind. = progression-free survival of re-induction; TFS = time to failure of strategy; OS = overall survival; CI = confidence interval; HR = hazard ratio; WT = hyperselection wild-type; MUT = hyperselection mutated. *P* values considered significant are displayed **bold.** | | | | | | | | |

| **Supplementary Table 6:** Updated adverse events for maintenance and re-induction treatment in the PanaMa trial. | | | | |
| --- | --- | --- | --- | --- |
| **Variable** | **Maintenance treatment**  **(n=248)** | | **Re-induction treatment**  **(n=128)** | |
|  | **FU/FA+pmab**  **(n=125)** | **FU/FA**  **(n=123)** | **Prior FU/FA+pmab**  **(n=50)** | **Prior FU/FA**  **(n=78)** |
| Any AE, n (%) | 122 (97.6) | 111 (90.2) | 46 (92.0) | 71 (91.0) |
| Grade ≥3 AE, n( %) | 76 (60.8) | 38 (30.9) | 20 (40.0) | 41 (52.6) |
| Grade 5 AE, n (%) | 3 (2.4) | 3 (2.4) | 2 (4.0) | 1 (1.3) |
| AE leading to permanent discontinuation of therapy, n (%) | 19 (15.2) | 2 (1.6) | 5 (10.0) | 11 (14.1) |
| Legend: AE = adverse event; FU/FA = 5-fluorouracil and folinic acid; pmab = panitumumab | | | | |
